# Microglial TDP-43 mediates myelin refinement and represses *Tyrobp* cryptic exon inclusion in mice

**DOI:** 10.1038/s41593-026-02348-3

**Published:** 2026-07-08

**Authors:** Anne-Claire Compagnion, Andranik Ivanov, Anil Rana, Felipe Espinoza, Thomas Sandmann, Fanny S. Martineau, Katia Monsorno, Roberta Facchinetti, Alessandro Matera, Lionel Rougé, Fernando González Ibáñez, Clarissa Catale, Matteo Bizzotto, Sonia Garel, Michela Matteoli, Yutaro Kashiwagi, Ryuta Koyama, Christian Haass, Marie-Eve Tremblay, Dieter Beule, Ileana Jelescu, Valerio Zerbi, Gilbert Di Paolo, Rosa C. Paolicelli

**Affiliations:** 1 Department of Biomedical Sciences, University of Lausanne, Lausanne, Switzerland; 2 Core Unit Bioinformatics, Berlin Institute of Health, Charité—Universitätsmedizin Berlin, Berlin, Germany; 3 Denali Therapeutics, South San Francisco, CA USA; 4 Department of Life Sciences, Health and Health Professions, Link Campus University, Rome, Italy; 5 Département de Médecine Moléculaire, Faculté de Médecine, Université Laval, Quebec City, Quebec Canada; 6 School of Medical Sciences, University of Victoria, Victoria, British Columbia Canada; 7 Institut de Biologie de l’Ecole Normale Supérieure (IBENS), Ecole Normale Supérieure, CNRS, INSERM, Université PSL, Team Brain Development and Plasticity, Paris, France; 8 Collège de France, Université PSL, Paris, France; 9 IRCCS Humanitas Research Hospital, Rozzano, Italy; 10 Humanitas University, Pieve Emanuele, Italy; 11 Department of Cellular Neurobiology, Graduate School of Medicine, The University of Tokyo, Tokyo, Japan; 12 Department of Translational Neurobiology, National Institute of Neuroscience, National Center of Neurology and Psychiatry, Tokyo, Japan; 13 Biomedical Center (BMC), Department of Anatomy and Cell Biology, Faculty of Medicine, Ludwig-Maximilians-Universität München, Planegg-Martinsried, Germany; 14 Munich Cluster of Systems Neurology (SyNergy), Munich, Germany; 15 German Center for Neurodegenerative Diseases (DZNE), Munich, Germany; 16 Department of Radiology, Lausanne University Hospital, Lausanne, Switzerland; 17 Department of Psychiatry, Faculty of Medicine, University of Geneva, Geneva, Switzerland; 18 Department of Basic Neurosciences, Faculty of Medicine, University of Geneva, Geneva, Switzerland

**Keywords:** Microglia, Neurodegeneration

## Abstract

TDP-43 proteinopathy is a hallmark of neurodegenerative disorders such as amyotrophic lateral sclerosis and frontotemporal dementia where mislocalization of TDP-43 has been observed in neurons and glial cells. However, the role of TDP-43 in microglia and the consequences of its loss of function remain unexplored. Combining magnetic resonance imaging, and confocal, and electron microscopy, we uncovered structural changes and myelin abnormalities in the early postnatal brain of mice lacking microglial TDP-43. Spatial transcriptomics further revealed an enriched interferon-responsive signature associated with oligodendrocyte dysfunction. Early depletion of microglial TDP-43 led to motor deficits in adult mice. Mechanistically, knocking out TDP-43 impaired microglial ability to engulf and degrade myelin. It also led to cryptic exon inclusion in the Tyrobp mRNA, resulting in truncated DAP12 protein, thus causing defective TREM2 signaling. Our findings reveal a role for TDP-43 in regulating the TREM2-DAP12 axis in mice, highlighting a previously unrecognized mechanism through which TDP-43 controls microglial function.

## Abstract (web summary)

Compagnion et al. show that TDP-43 loss in microglia causes myelin defects, oligodendrocyte precursor cell imbalance and motor deficits in mice. They identify TDP-43 as a regulator of the TREM2-DAP12 signaling, through the control of Tyrobp mis-splicing.

## Main

The TAR DNA-binding protein 43 (TDP-43), encoded by the *TARDBP* gene, is a highly conserved DNA/RNA-binding protein implicated in RNA processing that includes RNA splicing, mRNA stability and trafficking^1^. It is mainly located in the nucleus and is expressed in all cell types. TDP-43 aggregates, initially described in amyotrophic lateral sclerosis (ALS) and frontotemporal dementia (FTD)^2,3^, have emerged as a component of multiple neurodegenerative conditions, including Alzheimer’s disease (AD)^4,5^. Pathological cytoplasmic inclusions, characterized by hyperphosphorylated and ubiquitinated TDP-43, are associated with the loss of nuclear function, which, together with gain of toxicity, may contribute to pathological mechanisms^6^. TDP-43 proteinopathy in brain diseases has been ascribed primarily to neurons, with growing evidence implicating also glial cells, including astrocytes^7^, oligodendrocytes (OLs)^3^ and microglia^8^. However, the specific role of TDP-43 and the consequences of its loss of function in microglia have been scarcely explored.

TDP-43 regulates RNA splicing and suppresses cryptic exons (CEs) inclusion^9–12^. When TDP-43 is depleted, CEs are spliced into mRNAs, often introducing premature STOP codons and leading to nonsense-mediated decay^9^. The specific exons affected by TDP-43 splicing largely depend on the cellular identity, impacting different molecular pathways based on the cell type in which TDP-43 is dysregulated^10^. While TDP-43-dependent targets have been reported in diverse cell types^9,13–15^, the TDP-43-mediated regulation of CE in microglia remains unexplored.

Microglia, the innate immune cells of the central nervous system, have important functions in both physiological and pathological contexts^16^. Genome-wide association studies support their implication as risk factors in neurodegeneration, suggesting that microglia may have critical roles in modifying disease onset and severity^17^. Despite increasing evidence, their role in brain diseases remains underexplored. It is unclear whether dysfunctional microglial profiles associated with neurodegeneration could begin affecting the brain during early developmental stages. Given that microglia are essential for proper brain maturation and their impairment during critical developmental windows can have lasting effects on brain function^18–21^, investigating the role of microglial TDP-43 in brain development is crucial to understanding its potential early contribution to neurodegenerative processes.

Here combining diffusion magnetic resonance imaging (MRI) and confocal and electron microscopy (EM), we report the presence of microstructural alterations and myelin abnormalities in the early postnatal brains of mice lacking TDP-43 specifically in microglia and macrophages (cKO). These changes were mostly limited to the retrosplenial cortex/motor cortex (RSC/MC) and were associated with significant imbalance in the pool of oligodendrocyte precursor cells (OPCs) and mature OLs, and with aberrant myelination. Spatial transcriptomics revealed the emergence of altered signatures in cKO mice, including an upregulation in interferon (IFN)-responsive genes. Functionally, coculture experiments showed that microglia lacking TDP-43 led to reduced OPCs proliferation and accelerated myelination, consistent with the in vivo phenotype. Furthermore, TDP-43 knockout (KO) microglia displayed defective myelin phagocytosis, both in vitro and in vivo, suggesting their possible contribution to the described myelin abnormalities. In addition, we found that loss of TDP-43 in microglia results in the insertion of CE in the mRNA of the *Tyrobp* gene, leading to a truncated nonfunctional protein. Finally, cKO adult mice showed impaired motor behavior, supporting a key role for microglial TDP-43 in motor function. Notably, the motor behavior phenotype was prominent when microglial TDP-43 was depleted in the early postnatal brain, but not in young adult mice. This study provides evidence that selective microglial TDP-43 loss of function in the developing brain is sufficient to induce long-lasting myelination defects and motor behavior impairment in mice. We also report a mechanism through which TDP-43 regulates the TREM2-DAP12 signaling axis in the mouse brain.

## Results

### Depletion of TDP-43 alters microglial density and morphology in the postnatal brain

**Fig. 1:**
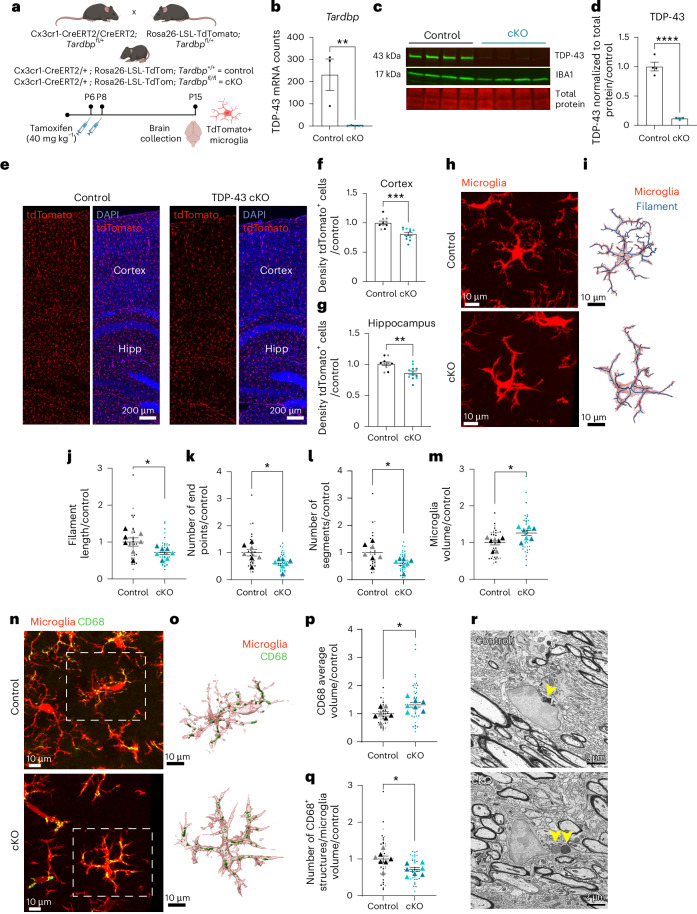
Microglial TDP-43 regulates microglial density and morphology in the postnatal brain. **a**, Scheme of the experimental breeding and timeline. **b**, Quantification of transcripts per million of *Tardbp* (exon 3) assessed by bulk RNA-seq performed on FACS-isolated microglia from control (*n* = 3) and TDP-43 cKO (*n* = 5) mice at P15, **P = 0.0043. **c**,**d**, Western blot of TDP-43 (**c**) and relative quantification in MACS-sorted microglia (**d**) of *n* = 4 control and *n* = 4 cKO mice at P15, ****P < 0.0001. **e**, Representative confocal *z*-stack projections of cortical region from control and cKO mice at P15. Scale bar = 200 µm. **f**,**g**, Quantification of microglial density in retrosplenial/motor/SSC (**f**; *n* = 10 control versus *n* = 11 TDP-43 cKO mice) and hippocampus (**g**; *n* = 10 control versus *n* = 12 TDP-43 cKO mice). **h**,**i**, Confocal acquisition (**h**) and 3D reconstruction (**i**) of tdTomato^+^ cell surface and microglial filaments. Scale bar = 10 µm. **j**, Quantification per cell of microglial filament length, *P = 0.0397. **k**, Number of end points, *P = 0.0179. **l**, Number of segments, *P = 0.0216; data in **j**–**l** are from *n* = 35 control cells and *n* = 41 cKO cells reconstructed from *n* = 7 control versus *n* = 7 cKO mice at P15. **m**, Microglia volume, *P = 0,0154. **n**,**o**, Representative image (**n**) and 3D reconstruction (**o**) of tdTomato^+^ cells and CD68^+^ structures. Scale bar = 10 µm. **p**,**q**, Quantification of average volume of CD68^+^ structures, *P = 0.0170 (**p**), and their number, *P = 0.0133 (**q**); data in **m**, **p** and **q** are from *n* = 41 control cells versus *n* = 37 cKO cells, reconstructed from *n* = 7 control versus *n* = 7 cKO mice at P15. **r**, EM micrographs of microglial cells from control and cKO mouse deep cortical layers at P15. Arrowheads point to lysosomal structures. Scale bars = 2 µm. In all the graphs, females are indicated in light and males in dark colors. For 3D reconstruction analyses, animals are represented with triangles, whereas cells are represented with circles. Statistics in **b**–**q** represent two-tailed unpaired *t* test performed on animals. Data are presented as mean ± s.e.m. Source data

**Extended Data Fig. 1:**
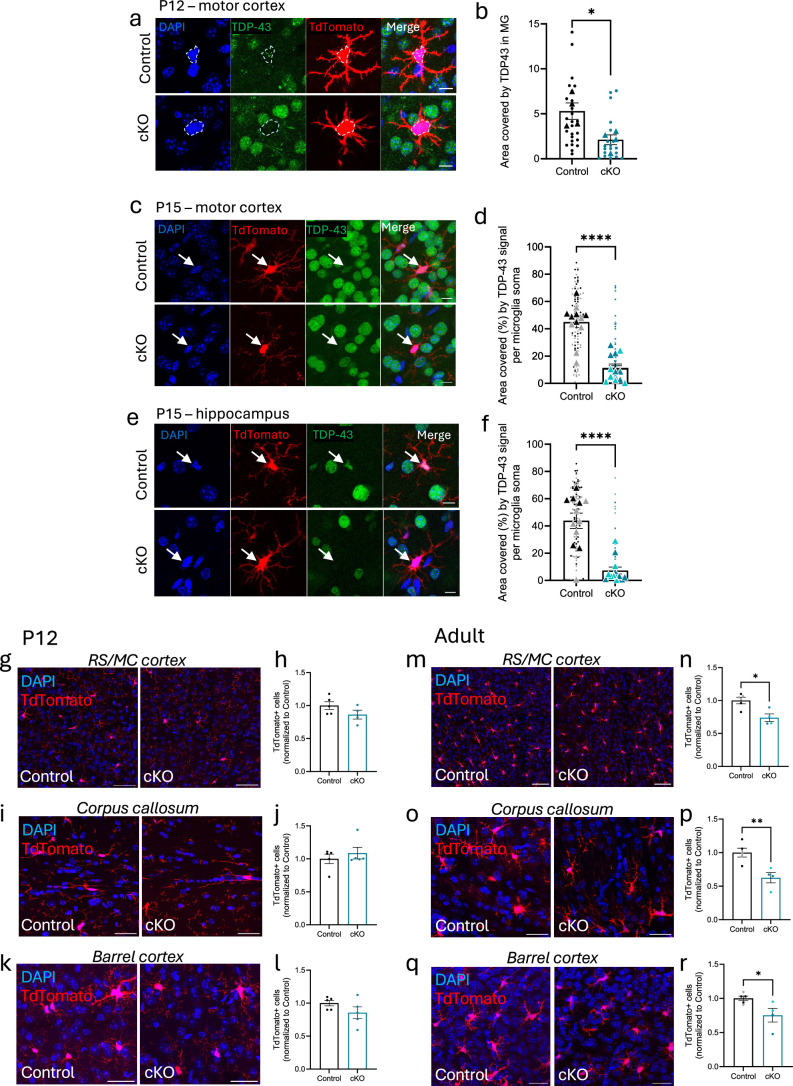
TDP-43 depletion in cKO mice and microglial density at P12 and adulthood. **a**, Representative confocal z-stack projections of TDP-43 immunoreactivity in the motor cortex of control and cKO mice at P12. White areas circle microglia cell soma. Scale bar: 10 µm. **b**, Quantification of the area covered by the TDP-43 signal per microglia soma. N = 25 cells from n = 4 controls and N = 20 cells from n = 4 cKO animals, *p = 0.0051. **c**,**e**, Representative confocal z-stack projections of TDP-43 immunoreactivity in the (**c**) motor cortex and (**e**) the hippocampus of control and cKO mice at P15. White arrowheads point at microglia cell body. scale bar: 10 µm. **d**,**f**, Quantification of the area covered by the TDP-43 signal per microglia soma in (**d**) the motor cortex: N = 104 cells from n = 12 controls, and N = 93 cells from n=12 cKO mice, ****p<0.0001, and in (**f**) the hippocampus: N = 117 cells from n = 12 controls and N = 113 cells from n = 12 cKO, ****p < 0.0001. **g**–**l**, Representative confocal z-stack projections of Tdtomato^+^ signal in the (**g**) motor cortex, (**i**) the corpus callosum, and (**k**) the barrel cortex of control and cKO mice at P12, and relative quantification in **h**, **j** and **l**. n = 5 control and 4 cKO mice. **m**, Representative confocal z-stack projections of confocal acquisitions of Tdtomato^+^ signal in the (**g**) motor cortex, (**i**) the corpus callosum, and (**k**) the barrel cortex of control and cKO mice at P12, and relative quantification in **h**, **j** and **l**. **m**–**r**, Representative confocal z-stack projections of Tdtomato^+^ signal in the (**m**) motor cortex, (**o**) the corpus callosum and (**q**) the barrel cortex in 4/5-month-old control and cKO mice, and relative quantification in (**n**) *p = 0.0105, (**p**) **p = 0.0074, and (**r**) *p = 0.0364. Scale bars: 50 μm, 30 μm, 30 μm for motor cortex, corpus callosum and barrel cortex, respectively. n = 5 control and 4 cKO mice. Statistics in **b**–**r**: two-tailed unpaired t-test, performed on animals. Data are presented as mean ± s.e.m. Females are indicated in light and males in dark colors. Animals are represented with triangles, whereas cells are indicated with circles. Source data

To investigate the role of microglial TDP-43 in brain development, we generated an inducible cKO mouse line by crossing Cx3cr1-CreERT2 and *Tardbp*-floxed strains, which also express Rosa26-LSL-TdTomato^21–23^. Our breeding strategy was designed to obtain all the experimental subjects as littermates, heterozygous for Cre expression and Rosa26-LSL-TdTomato, and either carrying *Tardbp*-floxed alleles or not, named cKO or control, respectively (Fig. 1a). Tamoxifen-mediated deletion of *Tardbp* was induced at postnatal (P) day P6 and P8, resulting in a significant reduction of both mRNA (Fig. 1b) and protein levels (Fig. 1c,d) in microglia, examined upon acute sorting from the brains of P15 mice. Quantification of TDP-43 immunoreactivity in microglia showed that protein reduction was already evident at P12 (Extended Data Fig. 1a,b), and further analyses confirmed depletion in cortical and hippocampal regions at P15 in cKO mice compared to controls (Extended Data Fig. 1c–f). Cre-mediated recombination induced tdTomato expression regardless of their genotype (Fig. 1e and Extended Data Fig. 1a–f).

We first asked whether TDP-43 depletion would affect microglial density and morphology in the early postnatal brain. cKO mice showed fewer microglia in the motor cortex/somatosensory cortex (SSC) and in the hippocampus (Fig. 1e–g), compared to control littermates. This phenotype, not observed yet at P12, was maintained into adulthood (Extended Data Fig. 1g–r). TDP-43 KO microglia also displayed reduced morphological complexity, as shown by shorter filament length, reduced number of end points and segments (Fig. 1h–l). Three-dimensional (3D) reconstruction of the tdTomato signal also revealed an increase in microglial volume (Fig. 1m). In addition, we examined phagocytic structures by measuring the volume and number of CD68^+^ phagolysosomes (Fig. 1n,o). KO cells showed an increased average volume and decreased number of phagocytic structures (Fig. 1p,q), further confirmed by EM (Fig. 1r), suggesting potential alterations in their phagocytic and/or degradative capacity.

### TDP-43 cKO mice show microstructural brain alterations and aberrant myelin structures

**Fig. 2:**
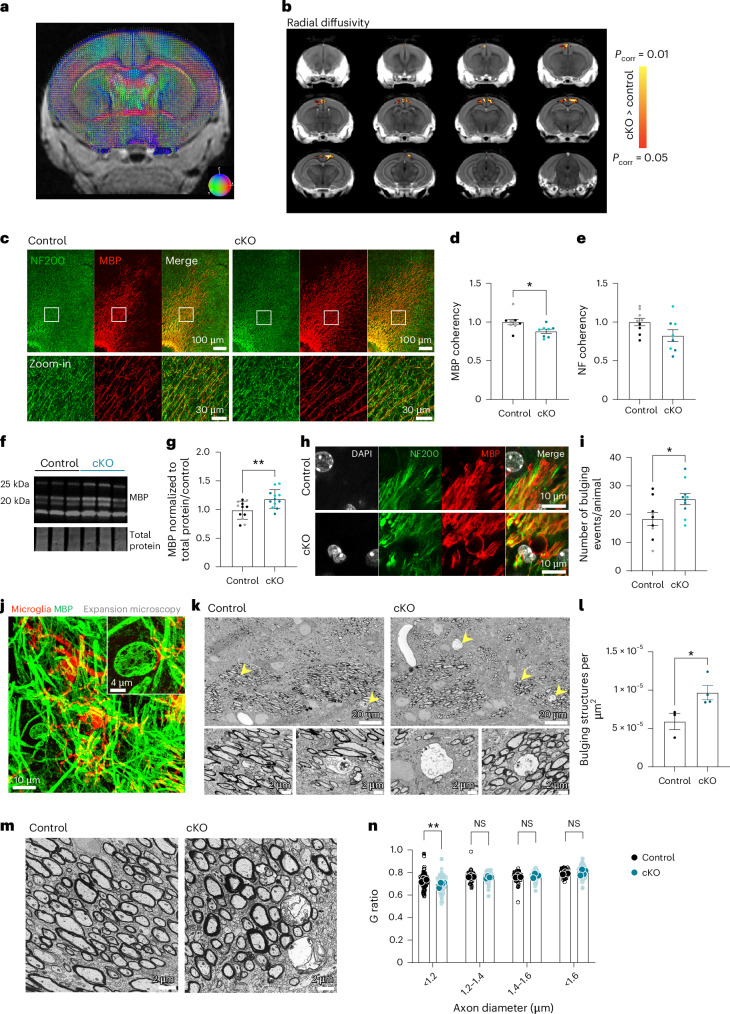
Microglial TDP-43 cKO induces microstructural brain alterations and aberrant myelin structures. **a**, DTI map illustrating voxel-wise preferential water diffusion within a representative coronal brain slice. The orientation map is color coded in HSV, integrating both angular inclination and directional information. The color sphere in the corner serves as a reference legend for orientation. **b**, Whole-brain *z*-score maps of voxel-wise differences in RD between control and cKO mice derived from diffusion MRI. Data include *n* = 6 control and *n* = 10 cKO P15 male mice. Statistical analysis was performed using randomized nonparametric testing with 5,000 permutations, TFCE correction and significance set at *P* < 0.05. **c**, Representative confocal acquisitions of MBP and NF200 immunostaining in the RSC/MC of control and cKO animals at P15. Scale bar = 100 µm; zoom-in, scale bar = 30 µm. **d**,**e**, Coherency analysis of MBP^+^ (**d**) and NF200^+^ (**e**) fibers in RSC/MC; *n* = 9 control versus *n* = 8 cKO animals at P15, *P = 0.0188. **f**, Western blot of MBP. **g**, Relative quantification of enriched motor/SSC homogenate of control and cKO animals at P15; *n* = 12 control versus *n* = 12 cKO animals, two-tailed unpaired *t* test, **P = 0.0065. **h**, Representative confocal acquisitions of MBP^+^ bulging structures in control and cKO P15 mice. Scale bars = 10 µm. **i**, Quantif**i**cation of bulging number from control and cKO animals at P15; *n* = 9 control versus *n* = 10 cKO animals, *P = 0.0332. **j**, Representative confocal acquisitions of MBP^+^ bulging events and tdTomato^+^ microglia using expansion microscopy (5× expansion). Scale bar = 10 µm; zoom-in, scale bar = 4 µm. **k**, Representative EM micrographs of bulging events from the deep cortical layers of control and cKO P15 male mice. Bottom, Zoom-in on bulging structures. Scale bar = 20 µm; zoom-in, scale bar = 2 µm. **l**, Quantification of frequency of bulging events, *n* = 3 control and *n* = 4 cKO animals, *P = 0.0444. Statistics in **d**–**l** represent two-tailed unpaired *t* test. **m**, Representative EM micrographs of myelin thickness. Scale bar = 2 µm. **n**, Mean myelin thickness per axon diameter; *n* = 544 control and *n* = 429 axons from *n* = 3 control and *n* = 4 cKO animals, **P = 0.0082, two-way ANOVA with Sidak’s multiple comparisons test. Females are indicated in light and males in dark colors. Data are presented as mean ± s.e.m. TFCE, threshold-free cluster enhancement; ANOVA, analysis of variance; NF, neurofilament. Source data

**Extended Data Fig. 2:**
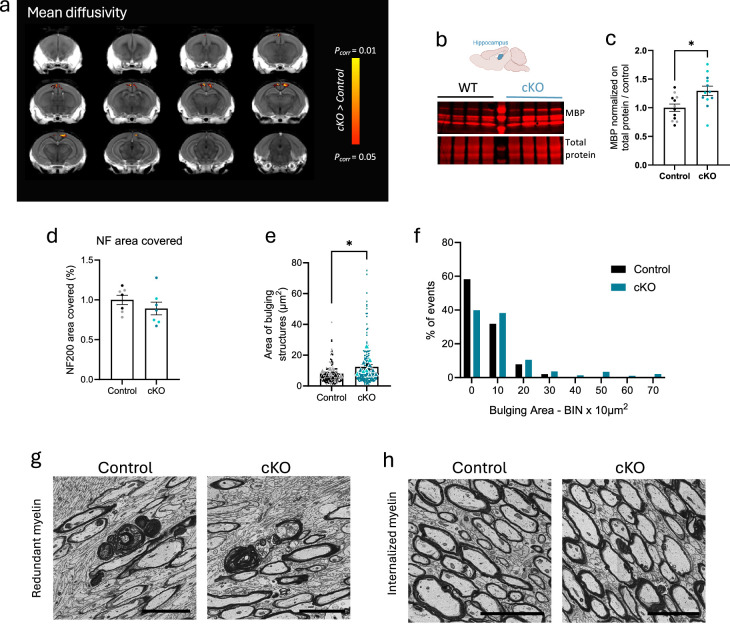
Myelin defects in cKO mice at P15. **a**, Whole-brain z-score maps of voxel-wise differences in mean diffusivity between control and cKO mice derived from diffusion MRI. Data include n = 6 control and n = 10 cKO P15 male mice. Statistical analysis was performed using randomized nonparametric testing with 5000 permutations, TFCE correction, and significance set at p < 0.05. **b**, Western blot of MBP from hippocampal homogenates at P15. **c**, Relative quantification, normalized to total protein stain. n = 12 control and n = 12 cKO mice of both sexes, *p = 0.0103. **d**, Quantification of the area covered by NF200 immunoreactivity, in the RSC/MC at P15 from n = 7 control vs n = 7 cKO mice at P15. **e**, Quantification of bulging size from n = 9 control and n = 10 cKO at P15, *p = 0.0189. Statistics in **c**–**e**: two-tailed unpaired t-test, performed on animals. **f**, Frequency distribution of bulging structures binned by size. N = 165 control vs N = 197 cKO bulging structures, reconstructed from n = 9 control vs n = 10 cKO mice. **g**,**h**, Representative EM micrographs of myelin aberrations in P15 control and cKO animals. Data are presented as mean values ± s.e.m. Source data

To comprehensively examine the large-scale structural consequences of microglial TDP-43 depletion, we conducted high-resolution diffusion MRI on whole fixed brains at P15 on a 7T preclinical MR scanner, followed by diffusion tensor imaging (DTI) analysis. Tensor modeling of the diffusion data (Fig. 2a) revealed a significant increase in radial and mean diffusivity (MD) in cKO mice, specifically in RSC/MC (Fig. 2b and Extended Data Fig. 2a), indicating microstructural alterations. The DTI analysis identified microstructural changes specifically in the direction perpendicular to axons or cellular processes (that is, radial diffusivity (RD); the MD is a weighted average of radial and axial diffusivities, whereby a change in the radial direction will also be reflected in the mean). Notably, alterations in myelin have been shown to translate into RD in both white and gray matter in patients with multiple sclerosis^24^, AD^25^ and FTD^26^, as well as in animal models of demyelination^27^. To explore this further, we performed myelin basic protein (MBP) and neurofilament NF200 immunoreactivity in the deep layers of the RSC/MC. Analysis of myelinated fibers revealed a decrease in their parallel alignment in cKO mice compared to littermate controls (Fig. 2c,d), whereas this was not evident for axons (Fig. 2c,e). These findings support the emergence of a disorganized myelin pattern when TDP-43 is depleted in microglia, and that this pattern is not dependent on axonal fiber organization. Total levels of MBP in neocortical structures and hippocampus of cKO mice were also found to be significantly increased (Fig. 2f,g and Extended Data Fig. 2b,c), whereas the area covered by NF200 remained unchanged (Extended Data Fig. 2d). High magnification confocal microscopy revealed the presence of MBP^+^ swellings of the myelin sheath, previously described in the early postnatal brain and defined as myelin bulging^28^. These structures, mostly localized in the deep layers of the motor cortex, did not contain nuclei or neurofilament proteins (Fig. 2h), and they were significantly larger and more numerous in cKO mice as compared to controls (Fig. 2i and Extended Data Fig. 2e,f). Expansion microscopy further provided examples of these bulging structures in close contact with microglial processes (Fig. 2j and Supplementary Video 1). EM analyses not only confirmed the increased frequency of myelin bulging in cKO mice (Fig. 2k,l) but also showed increased myelin thickness, mostly in small-diameter axons (Fig. 2m,n). In addition, myelin aberrations were observed in both control and cKO mice at P15 (Extended Data Fig. 2g,h), confirming previous reports that myelin abnormalities are highly abundant in the early postnatal brain^28^. Overall, these findings indicate that the loss of TDP-43 in microglia/macrophages exacerbates the emergence of myelin deficits at early postnatal stages.

### Loss of TDP-43 in microglia leads to defective myelin phagocytosis

**Fig. 3:**
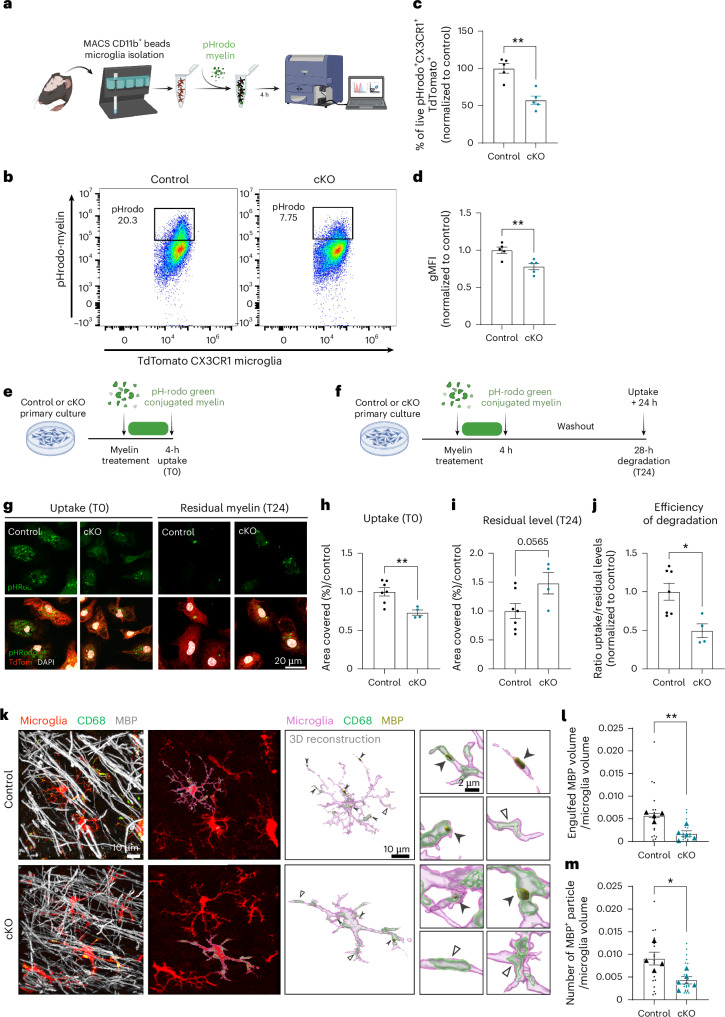
TDP-43 in microglia regulates myelin phagocytosis. **a**, Experimental scheme for myelin engulfment of MACS-isolated microglia from P15 animals assessed by FC. **b**, Representative FC plot for pHrodo-myelin inside tdTomato^+^ microglia, gated on live cells. **c**, Percentage of tdTomato^+^ microglia that phagocytose pHrodo-myelin, **P = 0.0010. **d**, gMFI, **P = 0.0069, *n* = 5 control and 5 cKO mice, over three independent experiments. **e**,**f**, Experimental scheme for myelin (**e**) engulfment and (**f**) degradation assay. **g**, Representative confocal *z*-stack projections of tdTomato^+^ primary control and TDP-43 KO microglia, exposed to pHrodo-conjugated myelin debris. T0 reflects myelin uptake and T24 residual level reflects degradation. Scale bar = 20 µm. **h**–**j**, Relative quantification of area covered by the pHrodo signal per cell at T0 (**h**; *n* = 152 control cells and *n* = 144 KO cells, **P = 0.0068), T24h (**i**; *n* = 190 control cells and *n* = 135 KO cells, *P* = 0565) and efficiency of degradation (**j**), *P = 0.0123. Data are from *n* = 7 control and *n* = 4 KO independent primary microglia preparations obtained from three independent experiments. **k**, Representative confocal images and their 3D reconstruction of microglial tdTomato^+^ cell surface and engulfed MBP inside CD68^+^ structures, in P15 control and TDP-43 cKO mice. Plain dark arrowheads point at MBP^+^ phagolysosomal structures, while empty white arrowheads point at MBP^−^ phagolysosomal structures. Left to right, scale bars = 10 µm, 10 µm, 2 µm. **l**,**m**, Quantification of microglial engulfment, showing volume, **P = 0.0021 (**l**) and number of MBP^+^ particles, *P = 0.0187 (**m**) per microglia volume, *n* = 15 control cells versus *n* = 19 cKO cells, reconstructed from *n* = 4 control versus *n* = 5 cKO mice. Statistics in **c**–**m** represent two-tailed unpaired *t* test, performed on animals. Data are presented as mean ± s.e.m. gMFI, geometric mean fluorescence intensity. Schematics in panels created in BioRender: **a**, Paolicelli, R. https://biorender.com/wuxcg3p (2026); **e**,**f**, Paolicelli, R. https://biorender.com/o1wuaqw (2026). Source data

**Extended Data Fig. 3:**
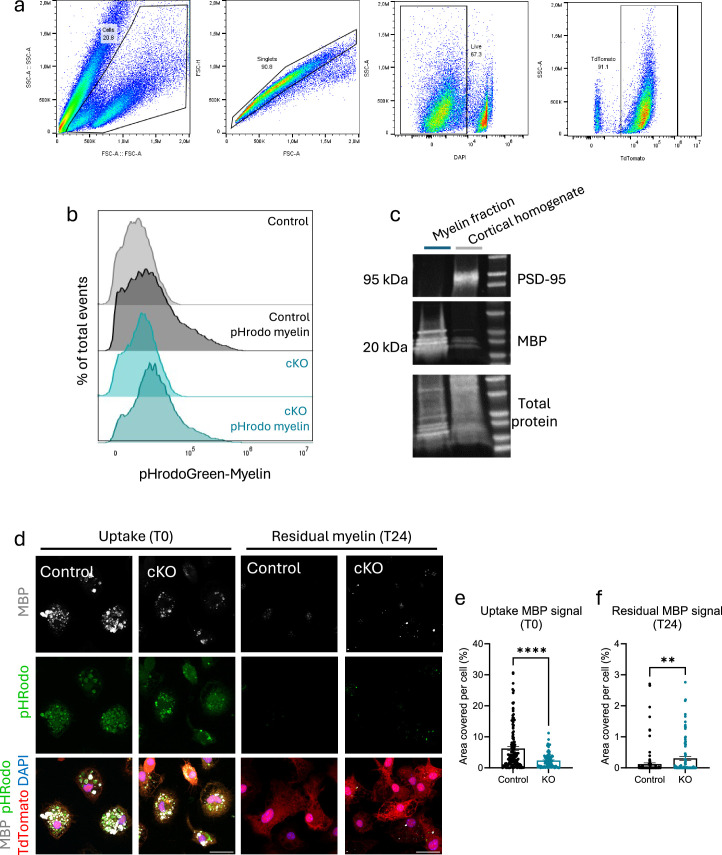
TDP-43 KO microglia have reduced uptake and decreased degradation of MBP^+^ structures in vitro. **a**, Gating strategy of live tdTomato^+^ microglia/macrophages cells for pHrodo-myelin engulfment analysis by FC. **b**, Percentage of total events for pHrodo-myelin engulfment in MACS-isolated microglia control or cKO. **c**, Validation of myelin extraction by western blot showing enrichment in MBP and absence of the synaptic marker PSD-95; compared to control cortex homogenate. **d**, Representative confocal z-stack projections of MBP immunostaining in tdTomato^+^ primary microglia control and TDP-43 KO, exposed to pHrodo-conjugated myelin debris. Myelin uptake (T0) and degradation (T24). Scale bar: 20 µm. **e**,**f**, Quantification of the area covered by MBP signal per cell (**e**) at T0 (N = 138 control cells and N = 97 cKO cells, ****p<0.01), reflecting uptake and (**f**) at T24 (N = 130 control cells and N = 91 cKO cells, **p = 0.0077), reflecting residual level following degradation. Statistics in **e** and **f**: two-tailed unpaired t-test. Data are presented as mean values ± s.e.m. Source data

Microglia have recently been shown to phagocytose and resolve myelin aberrations that form at early postnatal stages^28^. Therefore, considering the abnormal phagolysosomes in TDP-43 KO microglia (Fig. 1n–r) and the emergence of myelin aberrations in cKO mice, we wondered if this phenotype could reflect an altered microglial capacity for engulfing myelin. To address this question, we exposed acutely sorted microglia to myelin debris isolated from adult mice and conjugated to the pH-sensitive dye pHrodo^29^ (Fig. 3a). Flow cytometry (FC) analysis showed that, after 4 h, the number of microglia containing pHrodo-myelin was reduced by half in KO compared to controls, and the signal intensity was also significantly reduced (Fig. 3b–d and Extended Data Fig. 3a–c), indicative of a defect in myelin engulfment. We further confirmed these findings in primary microglia using an in vitro assay to discriminate between engulfment and degradation^30^. After 4 h of incubation with pHrodo-myelin, cells were either fixed (to quantify the amount of engulfed myelin) or washed and kept for a further 24 h to quantify the residual intracellular levels as an index of degradation (Fig. 3e,f). Quantification of both pHrodo and MBP signals within microglia confirmed that microglia from cKO mice engulfed significantly less myelin compared to controls (Fig. 3g,h and Extended Data Fig. 3d,e) and also displayed a reduction in the efficiency of myelin degradation, assessed as a ratio between the initial amount taken up after 4 h and the residual levels quantified after 24 h (Fig. 3g–j and Extended Data Fig. 3d,f). To investigate whether a similar defect in myelin engulfment could be observed in vivo in the early postnatal brain, we performed 3D reconstruction of tdTomato^+^ microglia, CD68^+^ phagolysosomes and MBP^+^ immunostained in the deep layer of the motor cortex of P15 control and cKO mice (Fig. 3k). Quantification of MBP^+^ puncta within CD68^+^ structures confirmed a significant reduction of the engulfed volume and a number of myelin particles within KO microglia as compared to controls (Fig. 3l,m), consistent with an impairment in myelin phagocytosis by microglia lacking TDP-43. Overall, these findings indicate that the loss of TDP-43 impairs the ability of microglia to efficiently phagocytose and degrade myelin, possibly underlying a defect in resolving myelin aberrations in the early postnatal brain.

### Loss of microglial TDP-43 induces a shift in the ratio between OPCs and mature OLs

**Fig. 4:**
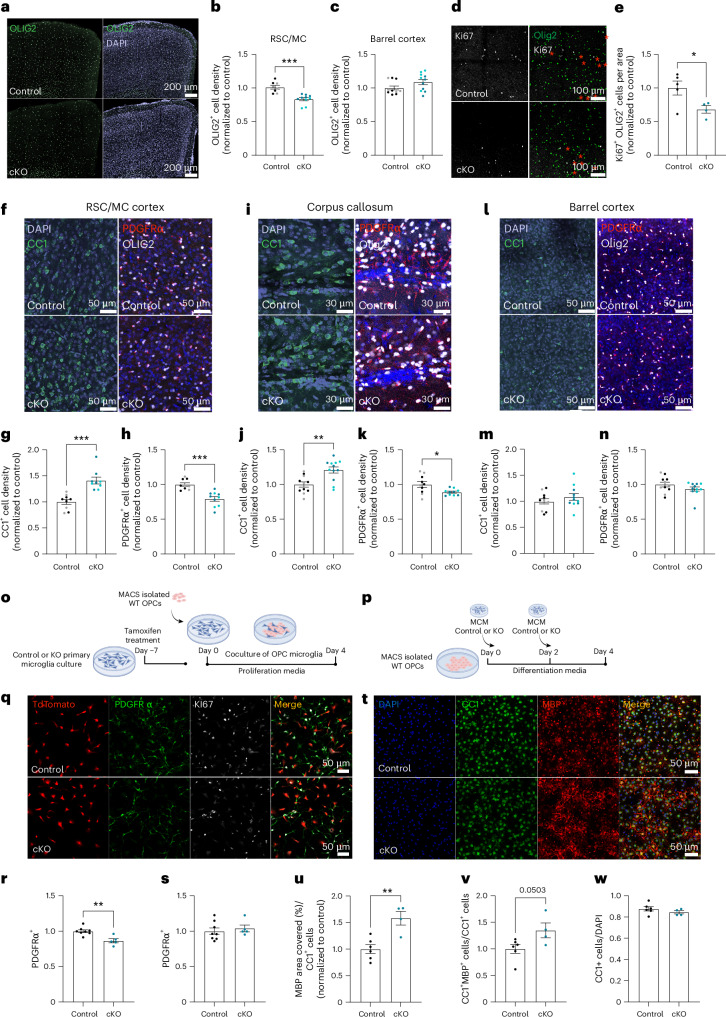
Microglial TDP-43 cKO induces a shift in the ratio of OPC versus mature OLs. **a**, Representative confocal *z*-stack projections of OLIG2 immunostaining in cortical region of control and cKO mice at P15. Scale bar = 200 µm. **b**,**c**, Relative quantification in RSC/MC (**b**; *n* = 8 control versus *n* = 10 cKO animals, ***P = 0.0001), and barrel cortex (**c**; *n* = 11 control versus *n* = 11 cKO animals). **d**, Representative confocal *z*-stack projections of OLIG2 and Ki67 immunostaining in cortical region of control and cKO male mice at P12. Scale bar = 100 µm. Red stars indicate Olig2^+^Ki67^+^ double-positive cells. **e**, Quantification of OLIG2^+^Ki67^+^ cells in control and cKO mice at P15. *n* = 5 control versus *n* = 4 cKO animals, *P = 0.042. **f**–**h**, Representative confocal *z*-stack projections of CC1 and OLIG2/PDGFRα immunostaining at P15 in the deep layers of RSC/MC (**f**), and relative quantification of CC1^+^ cell density (**g**; *n* = 9 control versus *n* = 9 cKO animals, ***P = 0.0001) and PDGFRα^+^ cell density (**h**; *n* = 9 control versus *n* = 10 cKO animals, ***P = 0.0001). Scale bar = 50 µm. **i**–**k**, The corpus callosum (**i**) and relative quantification of CC1^+^ cell density (**j**; *n* = 11 control versus *n* = 12 cKO animals, **P = 0.0019) and PDGFRα^+^ cell density (**k**; *n* = 10 control versus *n* = 10 cKO animals, *P = 0.0222). Scale bar = 30 µm. **l**–**n**, The barrel cortex (**l**) and relative quantification of CC1^+^ cell density (**m**; *n* = 10 control versus *n* = 10 cKO animals) and PDGFRα^+^ cell density (**n**; *n* = 10 control versus *n* = 10 cKO animals). Scale bar = 50 µm. **o**,**p**, Experimental scheme of OPC-microglia coculture experiments in proliferation media (**o**) and OPCs in differentiation media cultured with microglia conditioned media (**p**). **q**, Representative confocal acquisitions of Ki67 and PDGFRα immunostaining in WT OPCs cocultured with control or KO TdTomato^+^ microglia. Scale bar = 50 µm. **r**,**s**, Relative quantification of double-positive Ki67^+^PDGFRa^+^ cell density, **P = 0.0019 (**r**), and PDGFRα^+^ cell density (**s**), from *n* = 7 control versus *n* = 5 cKO animals over two independent technical replicates. **t**, Representative confocal acquisitions of CC1^+^ and MBP^+^ immunostaining in differentiating OPCs treated with control or KO microglia conditioned media. Scale bar = 50 µm. **u**–**w**, Relative quantification of percentage of MBP area covered normalized to total CC1^+^ cells, **P = 0.0039 (**u**), double-positive MBP^+^CC1^+^ cells normalized to total CC1^+^ cells, *P* = 0.0503 (**v**) and CC1^+^ cell density (**w**; two-tailed unpaired *t* test). From *n* = 6 control versus *n* = 4 cKO animals over two independent technical replicates. Statistics in **b**–**w** represent two-tailed unpaired *t* test. Data are presented as mean ± s.e.m. Schematics in panels created in BioRender: **o**,**p**, Paolicelli, R. https://biorender.com/p4w6d99 (2026). Source data

**Extended Data Fig. 4:**
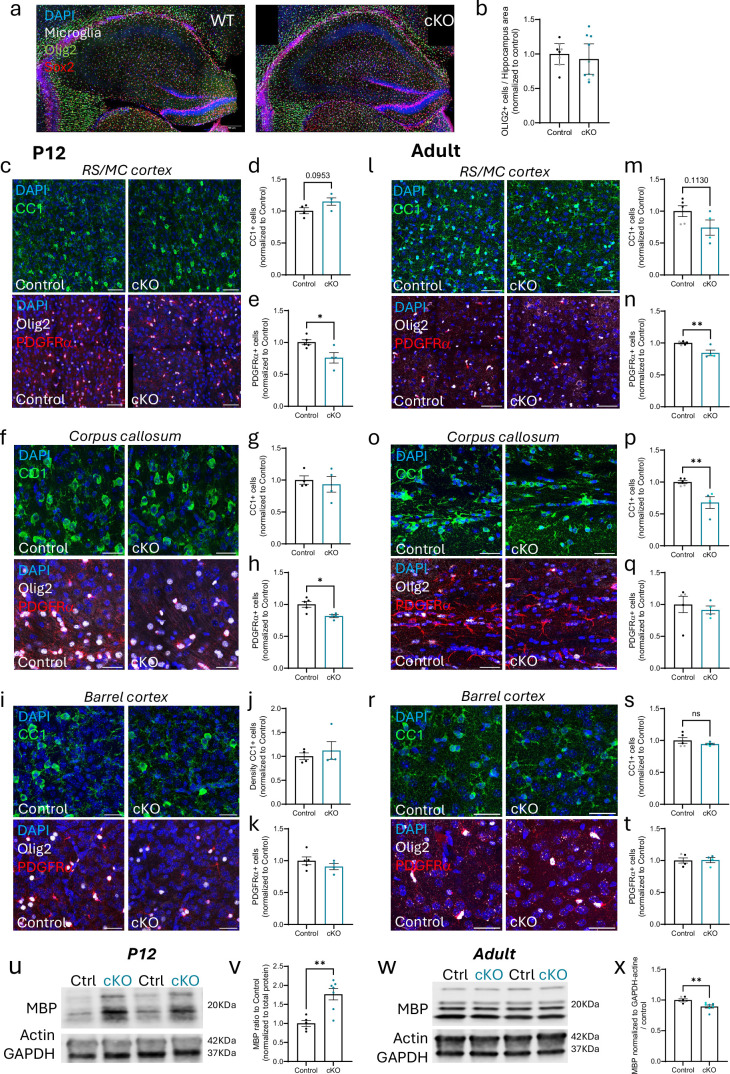
Oligodendrocyte density in the hippocampus is comparable between control and cKO mice at P15. **a**, Representative confocal z-stack projections of OLIG2 and SOX2 immunostaining in the hippocampus of control and cKO mice at P15. **b**, Quantification of OLIG2^+^ cell density in the hippocampus of control and cKO mice. n = 8 control and n = 10 cKO mice. **c**–**k**, Representative confocal z-stack projections of OLIG2, PDGFRα and CC1 immunostaining in (**c**) the motor cortex, (**f**) the corpus callosum, and (**i**) the barrel cortex of control and cKO mice at P12, and relative quantification of CC1^+^ in **d**, **g** and **j** and of PDGFRα in (**e**) *p = 0.0237, (**h**) *p = 0.0134 and (**k**); n = 4–5 controls and n = 4 cKO mice. **l**–**s**, Representative confocal z-stack projections of OLIG2, PDGFRα and CC1 immunostaining in (**l**) the motor cortex, (**o**) the corpus callosum, and (**r**) the barrel cortex of control and cKO 4–5 months old mice, and relative quantification of CC1^+^ in **m** and **p**, **p = 0.0072 and **s**, and PDGFRα in (**n**) **p=0.0079, and (**q**) and (**t**); n = 5 controls and n = 4 cKO mice. Scale bars: 50 μm, 30 μm, 30 μm for: motor cortex, corpus callosum and barrel cortex, respectively. Females are indicated in light and males in dark colors. **u**–**x**, Western blot for MBP in the enriched motor cortex of (**u**) P12 and (**w**) 4–5 months old control and cKO mice, and relative quantification in (**v**) **p = 0.0024 and (**x**) **p = 0.0064, respectively. P12: n = 5 controls and n = 7 cKO; Adults: n = 6 controls and n = 7 cKO. Statistics in **b**–**x**: two-tailed unpaired t-test, performed on animals. Females are indicated in light and males in dark colors. Stats are performed on animals. Data are presented as mean values ± s.e.m. Source data

Given the myelin phenotype and the increase in RD in the gray matter of cKO mice, we suspected that the pool of OLs may also be affected. Immunoreactivity for the pan-OL marker OLIG2 at P15 revealed indeed a significant reduction in cKO mice compared to controls, specifically in the RSC/MC (Fig. 4a,b), while no differences were observed in the adjacent barrel field cortex nor in the hippocampus (Fig. 4c and Extended Data Fig. 4a,b). Such a decrease could be at least in part explained by the reduced number of proliferating OLIG2^+^/Ki67^+^ cells in the brains of cKO mice (Fig. 4d,e). To investigate whether the decrease in OLIG2 was due to alterations in OPCs or in the mature OL pools, we quantified the density of CC1 and PDGFRα-immunoreactive cells, as proxies for mature OL and OPCs, respectively. In the deeper layers of the RSC/MC, where most of the OL are located, we found that, while the number of CC1^+^ mature OL was increased (Fig. 4f,g), the number of PDGFRα^+^ OPCs was decreased in cKO mice (Fig. 4f,h). These findings reveal an imbalance in the pool of mature compared to immature OLs in the myelinated cortical areas of cKO mice. A similar imbalance was observed in the corpus callosum (Fig. 4i–k), but not in the barrel field cortex (Fig. 4l–n), further highlighting the region specificity of the phenotype. The reduction in OPC density was already evident at P12, both in the motor cortex and in the corpus callosum (Extended Data Fig. 4c–h), and it persisted into adulthood (Extended Data Fig. 4l–q), whereas no differences were measured in the barrel cortex at any time points (Extended Data Fig. 4i–k and Extended Data Fig. 4r–t). In contrast, the increase in CC1^+^ cells observed at P12 and P15 was reversed in adulthood, with a prominent decrease in the corpus callosum, with no changes in the barrel cortex at any time point (Extended Data Fig. 4l–q). The age-dependent CC1^+^ profile is in accordance with the myelin levels, consistently increased at P12 and P15 (Extended Data Fig. 4u,v and Fig. 2f,g), but significantly decreased in adulthood (Extended Data Fig. 4w,x).

To investigate the direct contribution of microglial TDP-43 to the OPCs/OL imbalance, we established cocultures of either control or KO microglia with wild-type (WT) OPCs, maintained in proliferation or differentiation media (Fig. 4o,p). When cocultured with TDP-43 KO microglia, OPCs showed reduced Ki67 immunoreactivity, indicative of reduced proliferation, while no changes in the total number of PDGFRa^+^ cells were observed (Fig. 4p–r). On the other hand, CC1^+^ OLs showed increased myelin production when exposed to medium conditioned by KO microglia, as supported by the increase in both the total levels of MBP, and the fraction of CC1^+^ cells positive for MBP (Fig. 4t–v), with no changes in the density of CC1^+^ cultured cells (Fig. 4w). Altogether, these experiments provide evidence that microglial dysfunction, induced by the loss of TDP-43, is sufficient to alter the fine balance between OPCs proliferation and OL maturation.

### IFN-responsive signatures emerge in cKO mice

**Fig. 5:**
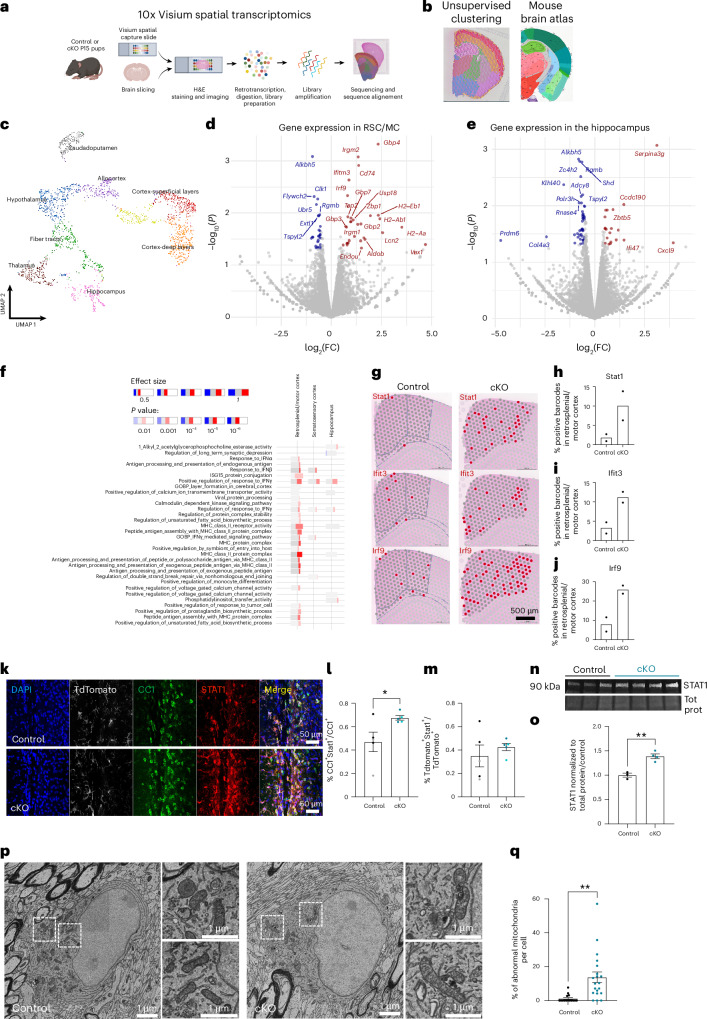
IFN-responsive signatures emerge in cKO animals. **a**, Scheme of the experimental spatial transcriptomics timeline. **b**, Representative graph-based cluster identification from spot level on brain coronal section (left) and corresponding section of the Allen Mouse Brain Reference Atlas (right). **c**, UMAP plot based on the transcriptional signature of each spot with their respective brain region attribution. **d**,**e**, Volcano plot of DEGs in RSC/MC (**d**) and SSC region (**e**; *n* = 2 control versus *n* = 2 TDP-43 cKO male mice). Genes that significantly increased with a log_2_(FC) > 0.5 are shown in red, whereas the ones decreased are in blue (cutoff, *P* < 0.05); Wald’s test, DESeq2 procedure. No *P*-value correction. **f**, Gene set enrichment analyses from RSC/MC, SSC and hippocampus. The size of the rectangle shows the effect size. The color-coded part of the rectangle represents the fraction of significantly upregulated (red) or downregulated (blue) genes. *P* values were calculated with the CERNO test from the tmod package. Multiple corrections were made using BH methods. **g**–**j**, Representative micrographs (**g**) and relative spot counts of Stat1 (**h**), Ifit3 (**i**) and Irf9 (**j**) across the RSC/MC spots of control and cKO mice (*n* = 2 control and *n* = 2 cKO). **k**–**m**, Representative confocal acquisitions of tdTomato, CC1 and STAT1 immunoreactivity (**k**), and relative quantification of the percentage of STAT1^+^CC1^+^ OLs, *P = 0,0442 (**l**), and tdTomato^+^ microglia (**m**). **n**,**o**, Western blot of STAT1 (**n**) and relative quantification in O4^+^ cells sorted from enriched MC/SSC of control and cKO male mice at P15, **P = 0.0020 (**o**), *n* = 3 control versus *n* = 4 cKO animals. **p**, Representative EM micrographs of OLs from the deep cortical layers of control and cKO P15 male mice. In the zoom-in, mitochondria are highlighted within a white-dotted box, showing mitochondrial abnormalities. Scale bar = 1 µm. **q**, Quantification of the percentage of abnormal mitochondria per cell, **P = 0.0016, *n* = 15 control and *n* = 21 cKO OLs cells from *n* = 3 control and *n* = 4 cKO animals. Statistics in **l**–**q** represent two-tailed unpaired *t* test. Data are presented as mean ± s.e.m. H&E, Hematoxylin and Eosine; DEGs, differentially expressed genes; UMAP, Uniform Manifold Approximation and Projection; BH, Benjamini–Hochberg. Schematic in **a** created in BioRender; Paolicelli, R. https://biorender.com/tfw0ld2 (2026). Source data

**Extended Data Fig. 5:**
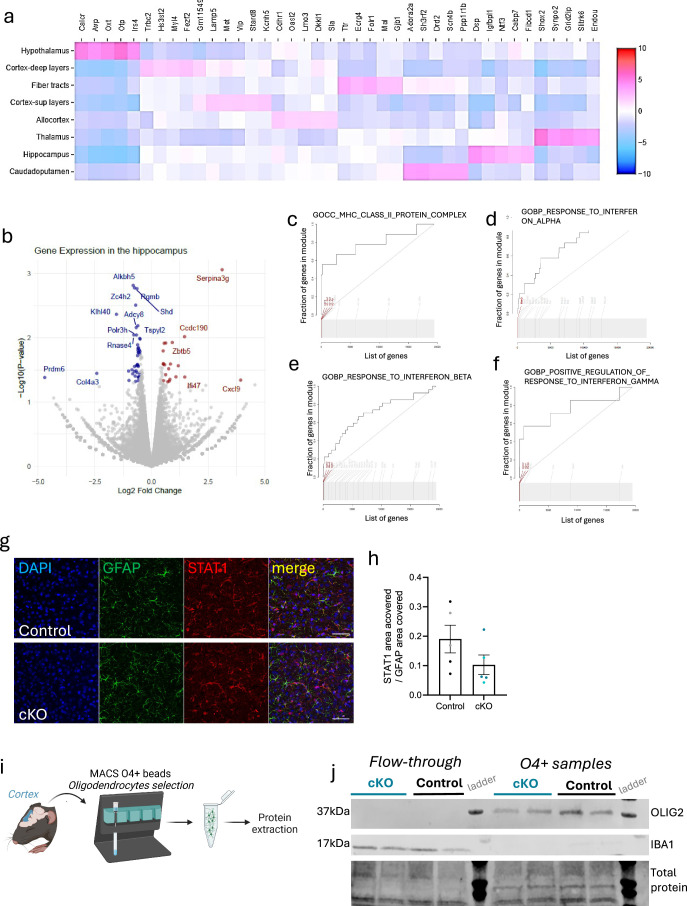
Validation of the specificity of spatial transcriptomic clustering and oligodendrocytes isolation strategy. **a**, Heatmap of canonical markers of unsupervised clustering of brain regions from spatial RNA sequencing data. **b**, Volcano plot of DEGs (differentially expressed genes) in the hippocampus (N = 2 control male vs N = 2 TDP-43 cKO male mice). Genes that significantly increased with a log_2_ fold change > 0.5 are shown in red, whereas the ones decreased are in blue (cutoff: adj. p-value < 0.05). **c**–**f**, Tmod evidence plots of (**c**) MHC-II, and (**d**–**f**) IFN-related pathways. **g**,**h**, Representative confocal acquisitions of (**g**) GFAP and STAT1 immunoreactivity, and relative quantification of (**h**) area covered by STAT1 signal within GFAP signal. **i**, Scheme of the O4^+^ cells magnetic sorting experiments (MACS). **j**, OLIG2 and IBA1 western blot on MACS O4^+^ isolated oligodendrocytes (O4^*+*^ samples) and O4^−^ (flow-through) isolated fraction, confirming the enrichment in oligodendrocyte markers. Data are presented as mean values ± s.e.m. Source data

To shed light on the molecular mechanisms underlying the observed cortical-related defects, we performed spatial transcriptomics on brain slices from control and cKO mice at P15 (Fig. 5a). Using unsupervised clustering, eight major regions were identified and validated by canonical markers (Fig. 5b,c and Extended Data Fig. 5a). We focused our comparison on three regions—RSC/MC (where the DTI differences were evident and where we specifically report OL alterations; Fig. 4), SSC and hippocampus (HP). Differential gene expression analyses between cKO and controls revealed genes significantly deregulated (*P* < 0.05; |log_2_(FC)|> 2) in different brain regions—70 in the RMC, 88 in SSC and 97 in HP (Fig. 5d,e, Extended Data Fig. 5b and Supplementary Table 1). Gene set enrichment analyses highlighted several pathways significantly dysregulated in cKO, many of which converged on antigen processing and presentation through major histocompatibility (MHC) protein complex and response to IFN, that were predominantly enriched in the RSC/MC region (Fig. 5f and Extended Data Fig. 5c–f). We found increased expression of several IFN-responsive genes such as *Ifit3*, *Irgm1*, *Irgm2*, *Stat1*, *Usp18*, *Zbp1*, *Ifi27l2a* and genes associated with MHC complex, such as *H2-Aa*, *H2-Ab1*, *H2-Eb1* (Fig. 5d,e). Counts of barcoded spots positive for IFN-responsive genes in the RSC/MC confirmed an enriched set size in cKO compared to controls (Fig. 5g–j). To identify which cell type was mostly driving the IFN response, we co-immunostained microglia, astrocytes and OLs with STAT1, a major mediator of the IFN cellular response. The percentage of CC1^+^ OLs expressing STAT1 was significantly higher in cKO mice (Fig. 5k,l), with no changes in microglia and astrocytes (Fig. 5k,m and Extended Data Fig. 5g,h). The increase in STAT1 was additionally validated by western blot in O4^+^ MACS-sorted OLs (Fig. 5n,o and Extended Data Fig. 5i,j). In addition, EM analysis of OLs revealed the presence of morphological abnormalities of mitochondria^31^, further supporting a state of cellular stress commonly associated with the IFN response^32^ (Fig. 5p,q).

### Microglial TDP-43 represses the expression of a CE in Tyrobp

**Fig. 6:**
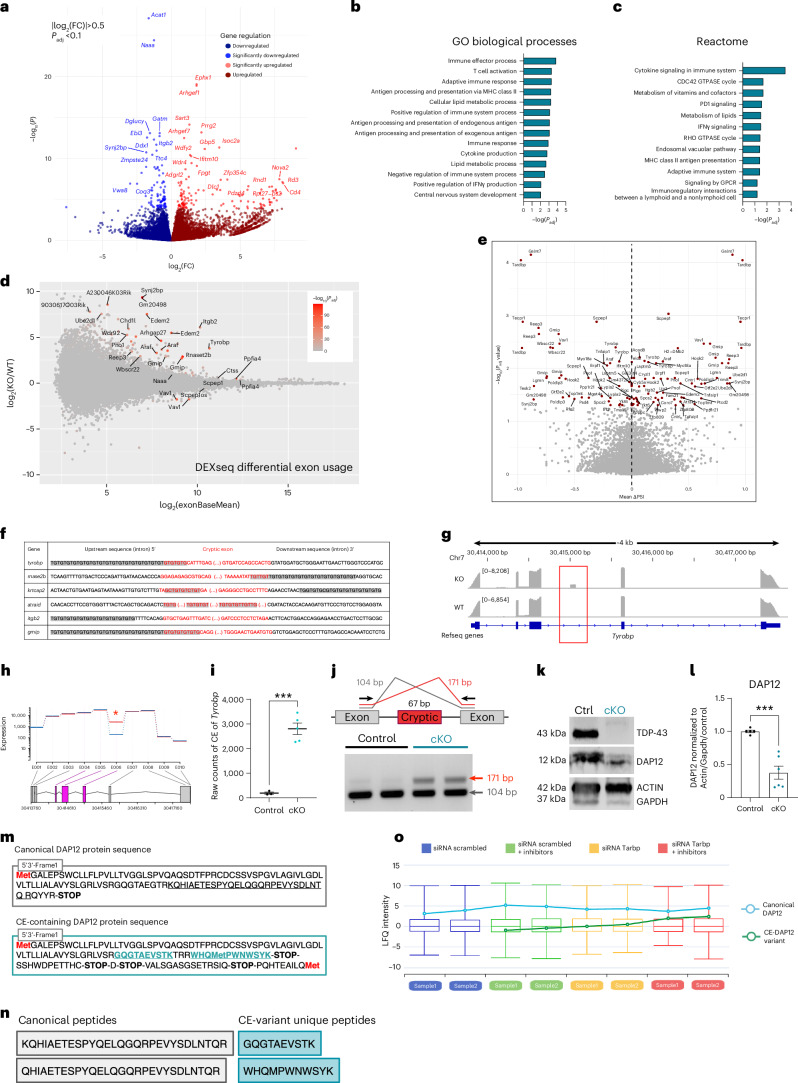
Microglial TDP-43 represses the expression of a CE in Tyrobp. **a**, Volcano plot of DEGs identified by bulk RNA-seq analysis (*n* = 3 control versus *n* = 5 TDP-43 cKO female mice). Genes significantly modulated with a|log_2_(FC)|> 0.5 are shown in red and blue, respectively (cutoff, *P*_adj_ < 0.1). *P* values were calculated with Wald’s test from the DESeq2 package. Multiple corrections were made using BH methods. **b**,**c**, GO biological processes (**b**) and significantly enriched pathways (**c**) in TDP-43 KO microglia identified by tmod analysis with Msig-DB reactome gene sets. Adjusted *P* values are initially calculated using hypergeometric test. **d**, MA plot of DEU in KO microglia. On the *y* axis is the average expression of the exon and on the *x* axis the log_2_(FC) between the KO and controls. *P* values were calculated with the likelihood-ratio test from the DEXseq package. Multiple corrections were made using BH methods. **e**, MAJIQ results for the differential splice site usage. *X* axis is the mean ΔPSI; *y* axis is −log_10_(adj *P* value), *t* test. *P* values were calculated with a two-sided Welch’s *t* test on posterior mean PSI values from the MAJIQ package. Multiple corrections were made using BH methods. **f**, Tables showing the TG (UG on mRNA) tandem repeats that exist upstream, downstream or within CEs. **g**, IGV browser view of the read coverage of the *Tyrobp* gene locus. **h**, Sashimi plot for *Tyrobp* mRNAs. Red star shows the specific CE inclusion. **i**, Quantification of transcripts per million of *Tyrobp* CE from *n* = 3 control versus *n* = 5 cKO mice, ***P = 0.0002. Data are presented as mean ± s.e.m. **j**, Representative scheme of the expected amplicon containing or not the CE (top) and RT–PCR results (bottom) using a forward primer on exon 3 and a reverse primer on exon 4. The amplicon of canonical *Tyrobp* (lower band, gray arrow) is 104 bp, while the amplicon containing the CE (upper band, red arrow) is 171 bp. **k**, Western blot of TDP-43 and DAP12. **l**, DAP12 relative quantification in MACS-isolated microglia from *n* = 4 control and *n* = 6 cKO mice at P15, ***P = 0.0003. Statistics in **i**–**l** represent two-tailed unpaired *t* test; data are presented as mean ± s.e.m. **m**, DAP12 predicted protein sequence from canonical and CE version of *Tyrobp* sequence (Expasy database), showing the predicted frameshift and insertion of STOP codons. **n**, Peptides identified through MaxQuant for univocal identification of canonical and CE variant of DAP12, as underlined in **m**. **o**, Relative LFQ intensity of canonical and CE-DAP12 peptides through MaxLFQ algorithm in BV2 microglia-like cells, control (siRNA scrambled) or TDP-43 KD (siRNA Tardbp) treated or not with protein degradation inhibitors (100 µM Bafilomycin A and 1 µM MG132 for 6 h). Data are displayed per sample, prepared over two independent biological replicates. Box plots of log_2_ LFQ intensities of total protein per sample. The centerline represents the median; the box bounds the IQR (25th–75th percentiles); whiskers extend to 1.5× IQR. IGV, Integrative Genomics Viewer; GO, Gene Ontology; LFQ, label-free quantification; IQR, interquartile range; KD, knockdown. Source data

**Extended Data Fig. 6:**
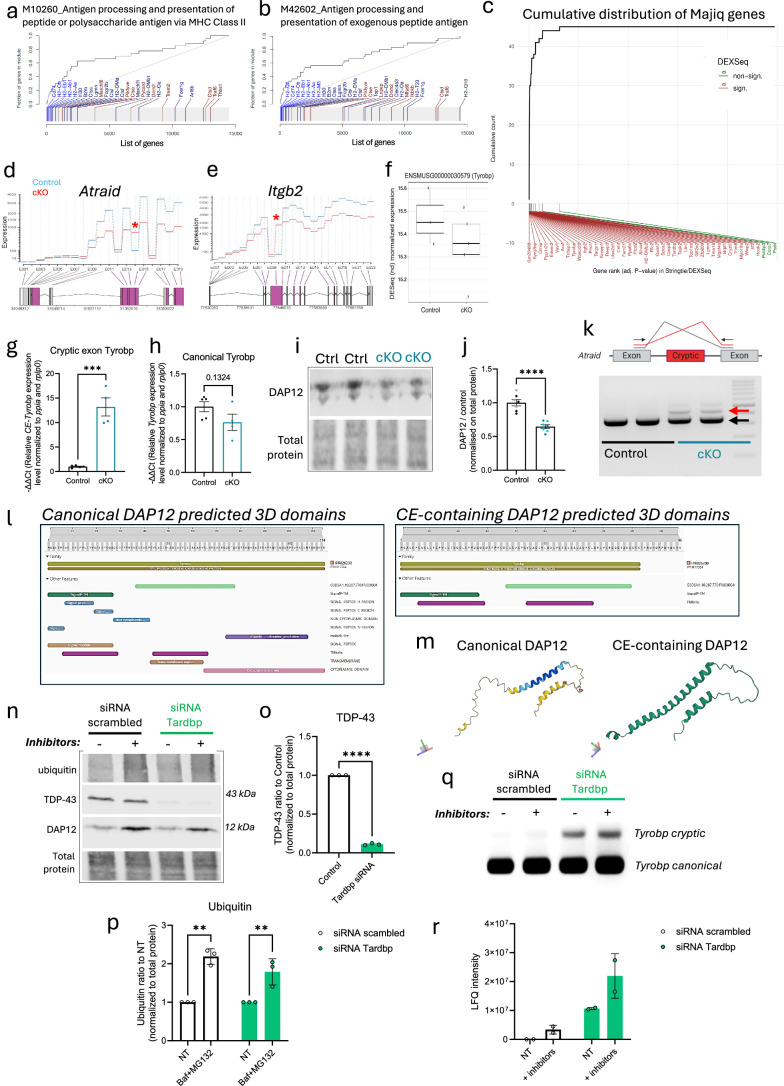
TDP-43 KO in microglia induces cryptic exons insertion and leads to predicted alterations of DAP12 protein sequence. **a**,**b**, Evidence plots in **a** and **b** showing enriched 24 gene sets in TDP-43 KO microglia (the name of the gene set is reported on top of the graph). X axis represents the full list of genes sorted by their p-values. Y axis is the cumulative fraction of genes from that gene set. Genes in blue are downregulated and red are upregulated in the TDP-43 KO. Light blue and light red correspond to significant deregulation (adj. P-value <0.1). **c**, Comparison of DEXSeq and MAJIQ results. X axis represents all genes sorted by the DEXSeq adjusted P-value; Y axis is the cumulative count of MAJIQ significant gene set. **d**,**e**, DEXSeq sashimi plot for (**d**) *Atraid* and (**e**) *Itgb2* mRNAs. Red stars show the specific cryptic exon inclusions. **f**, Regularized log-transformed counts of *Tyrobp* gene in KO and control RNA-seq. **g**,**h**, Quantification of (**g**) CE-*Tyrobp* sequence, ***p = 0.0001 and (**h**) canonical *Tyrobp* mRNA by qRT-PCR, from control (n = 5) and cKO (n = 4) primary microglia cultures. **i**, Western blot for DAP12 in brain cortical homogenate at P15. **j**, Relative quantification, from n = 7 controls and n = 7 cKO mice, ****p < 0.0001. **k**, Representative strategy of PCR amplification of *Atraid* cryptic exon (upper part) and RT-PCR results (bottom part) of amplicon of canonical *Atraid* (lower band, black arrow) or containing the cryptic exon (upper band, red arrow). **l**, DAP12 predicted protein sequence from canonical and cryptic exon version of *Tyrobp* sequence (Expasy database). **m**, Predicted 3D protein structures of canonical and CE-DAP12 based on amino acid sequence produced with AlphaFold modeling. **n**, Western blot of ubiquitin, TDP-43 and DAP12 in BV2 microglia-like cells transfected with scrambled siRNA or Tardbp siRNA, and treated or not with protein degradation inhibitors (100 µM Bafilomycin A and 1 µ MG132 for 6 h). **o**, Validation of TDP-43 knockdown, ****p < 0.0001. **p**, Ubiquitin levels (siRNA scrambled NT vs. GC Baf+MG132: **p = 0.0018, and siRNA Tardbp NT vs. TDP-43 KD Baf+MG132: **p = 0.0081) in BV2 cells compared to scrambled controls. N = 3 per condition. **q**, PCR amplification of the canonical *Tyrobp* (lower band) and the amplicon containing the cryptic exon (upper band) in BV2 cells control (siRNA scrambled) or TDP-43 knockdown (siRNA Tardbp) treated or not with protein degradation inhibitors. **r**, Label-free quantification (LFQ) intensity of DAP12 cryptic variant peptides using MAxQuant. Statistics in **g**–**o**: two-tailed unpaired t-test, and in **p**: RM-two-way ANOVA. Data are presented as mean values ± s.e.m. Source data

Next, we performed bulk RNA sequencing (RNA-seq) analysis of microglia acutely isolated from the RSC/MC to gain a deeper understanding of their transcriptomic profile, as microglial genes were under-represented in the spatial RNA-seq data. Differential expression analysis revealed that 405 genes were significantly (*P*_adj_ < 0.1) deregulated (|log_2_(FC)|> 0.5) in KO compared to control microglia (Fig. 6a and Supplementary Table 2). Gene Ontology analysis highlighted the enrichment of pathways implicated in immune response, lipid metabolism and antigen processing and presentation through MHC complex (Fig. 6b,c). Several MHC-related genes (*Cd74*, *H2-Aa*, *H2-Ab1*, *H2-Eb1*) were significantly downregulated (Extended Data Fig. 6a,b), indicating an impairment in microglial immune function. One of the roles of TDP-43 is to repress CE inclusion during RNA splicing^9^. To investigate whether and how TDP-43 depletion in microglia affects RNA splicing, we performed de novo exon annotation followed by differential exon usage (DEU) analysis. TDP-43 depletion in microglia led to a substantial deregulation in exon expression, with DEXSeq analysis identifying 4,346 differential splicing events in over 2,000 genes, including CEs, 3′UTR elongations, and alternative 3′ and 5′ splice sites (Fig. 6d and Supplementary Table 3). To further corroborate these findings, we also used MAJIQ V3 (ref. ^33^), an algorithm that incorporates read-mapping-based guided de novo genome reference annotation and a statistical framework to identify significant changes in splice site usage across conditions. MAJIQ identified 88 significant (*t* test (Benjamini–Hochberg *P*_adj_ < 0.05, |ΔPSI| > 0.1)) changes in splice site usage in 45 different genes between cKO and controls (Fig. 6e and Supplementary Table 4). Notably, almost all MAJIQ results were included within the significant list of DEXSeq, including *Atraid*, *Itgb2*, *Synj2bp*, *Ltn1*, *Tecpr1* and others (Extended Data Fig. 6c–e). For several of these genes, we confirmed the presence of the UG-rich TDP-43-binding motif in the intronic sequences flanking the CE (Fig. 6f). Notably, we discovered that TDP-43 suppressed the expression of a 67-nucleotide-long CE between the third and fourth constitutive exons of the *Tyrobp* gene, with a percent spliced-in index (PSI) of 11–14% (Fig. 6g,h and Supplementary Table 3). *Tyrobp* encodes DAP12, a key immunoreceptor tyrosine-based activation motif-bearing transmembrane adaptor protein, required for the signal transduction of surface receptors such as TREM2 (ref. ^34^). The raw counts of the CE sequence confirmed the increase in the microglia from cKO mice compared to controls (Fig. 6i), while the overall *Tyrobp* gene counts were unchanged (Extended Data Fig. 6f). To validate the inclusion of the putative CE within the mRNA of *Tyrobp*, we designed two primers annealing at the end of exon 3 and at the beginning of exon 4, respectively. PCR amplification using the cDNA retrotranscribed from the RNA of acutely isolated microglia led to the detection of one single band in control cells, but a longer amplicon in KO cells, confirming the inclusion of the CE in the absence of TDP-43 (Fig. 6j). RT–qPCR confirmed the significant increase in CE-*Tyrobp*, while the overall mRNA was unchanged (Extended Data Fig. 6g,h). CE insertion often leads to premature STOP codon and protein degradation. Western blot quantification of DAP12 protein in microglia acutely isolated at P15 confirmed a significant decrease in TDP-43 KO cells compared to control (Fig. 6k,l), and similar results were obtained from whole-brain homogenates at P15 (Extended Data Fig. 6i,j).

Additional validation was performed for *Atraid*, another identified target gene, for which we confirmed the presence of CE inclusion (Extended Data Fig. 6k).

Consistent with this, bioinformatic analyses predicted that the CE inclusion in *Tyrobp* would lead to a frameshift introducing a premature STOP codon and consequent loss of the protein C-terminal domain (Fig. 6m and Extended Data Fig. 6l). Prediction of the 3D protein structure was further supported using AlphaFold modeling^35^ (Extended Data Fig. 6m). Finally, we performed targeted proteomics for the detection of both the canonical and the CE-containing DAP12 protein, in the presence or absence of TDP-43 (Fig. 6n). We used BV2 microglial cell lines in which TDP-43 was efficiently knocked down as compared to scrambled siRNA control (Extended Data Fig. 6n,o), and in which we could also confirm the presence of CE-*Tyrobp* (Extended Data Fig. 6p). Proteomics was performed on cell lysate (<15 KDa fraction) from cells either incubated with a cocktail of proteasomal and lysosomal inhibitors for 6 h, or left untreated. Efficiency of protein degradation inhibition was confirmed by significant accumulation of ubiquitin in the treated cells (Extended Data Fig. 6n,q). Label-free quantification intensity analysis was performed using MaxQuant^36^ and manually validated using Skyline^37^ (Supplementary Table 5). Notably, CE-DAP12 was undetectable in control cells under basal conditions, and it was detected only upon inhibition of the protein degradation machinery. In contrast, CE-DAP12 was already detectable at baseline in TDP-43 knockdown cells, and its levels further increased after inhibition of protein degradation (Fig. 6o and Extended Data Fig. 6r). Overall, these findings demonstrate that TDP-43 controls the repression of CEs in microglia and identify DAP12 signaling as one of its targets.

### CE-containing Tyrobp mRNA contributes to defective DAP12 intracellular signaling

**Fig. 7:**
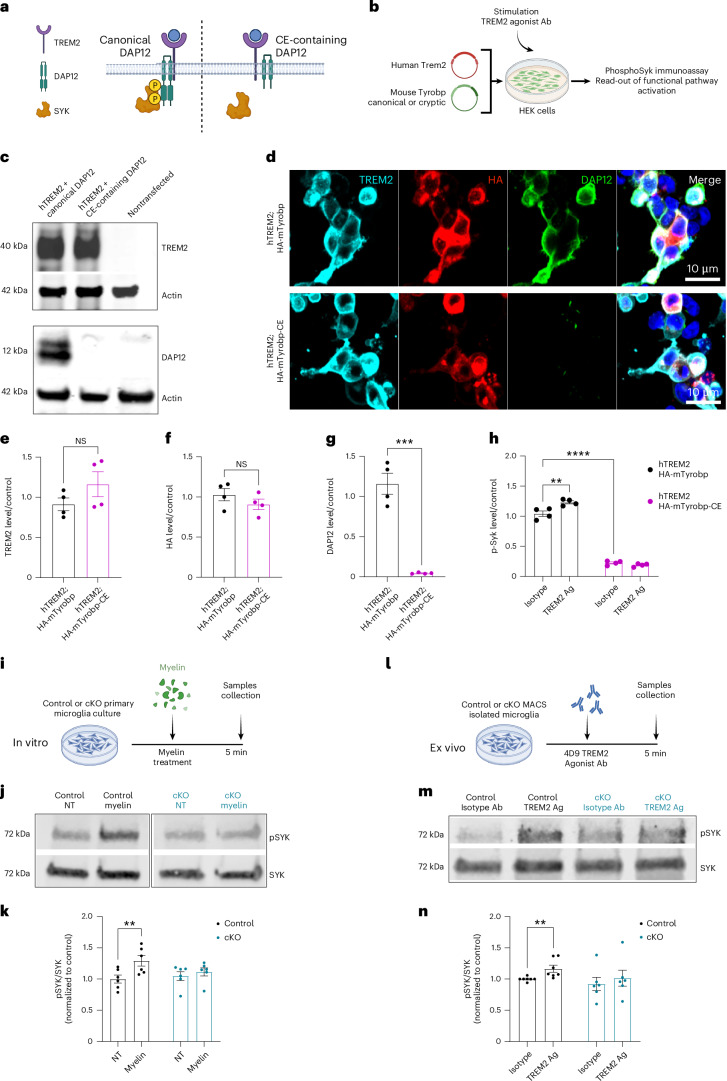
TDP-43 KO in microglia contributes to defective intracellular signaling in CE-containing Tyrobp. **a**, Scheme of the predicted loss of function of DAP12 containing the CE. **b**, Experimental design to overexpress human TREM2 and canonical or CE-containing DAP12 in HEK293 cells. **c**, Western blot of DAP12 and TREM2 on cell lysates from HEK293 cells transfected with canonical or CE-containing *Tyrobp* sequence. **d**–**g**, Representative confocal images of TREM2, HA and DAP12 immunostaining of HEK293 cells transfected with HA-tagged canonical or CE-containing *Tyrobp* sequence (**d**), and relative quantification of TREM2 (**e**), HA (**f**) and DAP12 signal (**g**), ***P < 0.0001. Statistics in **e**–**g** represent two-tailed unpaired *t* test. **h**, SYK phosphorylation quantified in HEK293 cells from experiment in **d** after TREM2 agonist antibody or isotype control antibody stimulation using phosphorylation immunoassay. Two-way ANOVA, Tukey’s multiple comparison test, **P = 0.0026, ****P < 0.0001, *n* = 4 independent experiments. **i**, Experimental design for myelin-induced pSYK assay in primary control and TDP-43 KO microglia. **j**, Western blot of pSYK and SYK. **k**, Relative quantification of the signal, 5 min after myelin treatment. Repeated-measures two-way ANOVA, **P = 0.0023, *n* = 6 control and *n* = 6 cKO over two independent experiments. **l**, Experimental design for 4D9 TREM2 agonist antibody-induced pSYK assay in microglia MACS sorted from control and TDP-43 cKO P15 mice. Isotype antibody is used as control. **m**, Western blot of pSYK and SYK. **n**, Relative quantification of the signal, 5 min after antibody treatment. RM two-way ANOVA, **P = 0.0086, *n* = 7 control and *n* = 6 cKO over three independent experiments. Data are presented as mean ± s.e.m. RM, repeated measures. Schematics in **a**, **b**, **i** and **l** created in BioRender: **a**,**b**, Paolicelli, R. https://biorender.com/c6rpd39 (2026); **i**,**l**, Paolicelli, R. https://biorender.com/jrm5x8d (2026). Source data

**Extended Data Fig. 7:**
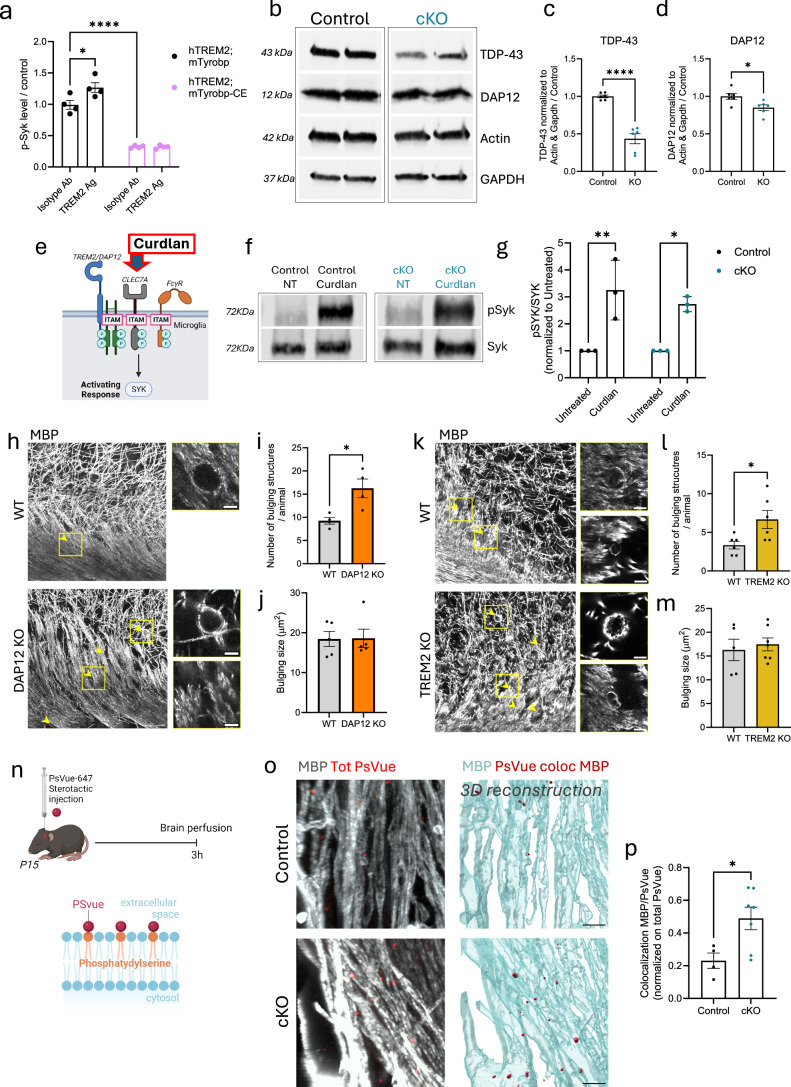
Myelin-bulging phenotype is partially recapitulated in TREM2 and DAP12 KO mice. **a**, SYK phosphorylation quantified in HEK293 cells from experiment after TREM2 agonist antibody stimulation using phosphorylation immunoassay. Two-way ANOVA, Tukey’s multiple comparison test *p = 0.0162, ****p < 0.0001, n=4 independent experiments. **b**–**d**, Western blot of (**b**) TDP-43 and DAP12 and relative quantification of (**c**) TDP-43, ****p < 0.0001 and (**d**) DAP12, *p = 0.0187, in control and TDP-43 KO primary microglia cells. n = 6 control and n = 6 KO cultures (from 2 independent experiments). **e**, Scheme of the mode of action and pathway activated by the CLEC7A agonist Curdlan. **f**, Western blot of pSYK and SYK. **g**, Relative quantification of the signal, 30 min after Curdlan treatment. *p = 0.0119; **p = 0.0026, n = 3 control and n = 3 cKO over three independent experiments. **h**–**j**, Representative confocal images (**h**) and quantification of (**i**) number, *p = 0.0170 and (**j**) size of bulging structures in control and DAP12 KO male mice; n = 5 control and n = 5 KO mice at P15. Arrowheads point to bulging structures. Scale bar: 20 µm, zoom-in: 5 µm. **k**–**m**, Representative confocal images (**k**) and quantification of (**l**) number, *p = 0.0257 and (**m**) size of bulging structures in control and TREM2 KO male mice. n = 5 control and n = 5 KO animals at P16. Arrowheads point to bulging structures. Scale bar: 20 μm, zoom-in: 5μm. **n**, Experimental design of PSVue intracerebroventricular (ICV) stereotactic injections in P15 mice. **o**, Representative z-stack projections of confocal acquisitions of MBP immunostaining and PSVue in the motor cortex (left) and 3D reconstruction (right) of control and cKO mice at P15, 3 h postinjection. Scale bar: 1 µm. **p**, Volume of colocalization between double PSVue^+^ and MBP^+^ puncta quantified in the deep cortical layers of control and TDP-43 cKO mice at P15, 3 h postinjection, *p = 0.0280. n = 4 control and n = 7 cKO animals. Statistics in **c** and **d** and **i**–**p**: two-tailed unpaired t-test; in **g**: two-way ANOVA, Sidak’s multiple comparison test. Data are presented as mean values ± s.e.m. Source data

Upon ligand binding, the TREM2 receptor recruits the adaptor protein DAP12 and induces rapid phosphorylation of the kinase SYK^38^. We hypothesized that the TREM2-DAP12 signaling pathway would be compromised in TDP-43 KO microglia, resulting in defective SYK activation (Fig. 7a). To gain mechanistic insights, we cloned the coding sequence of the mouse *Tyrobp* gene tagged with HA, with or without the CE, and overexpressed it in HEK293 cells along with human TREM2 (Fig. 7b). The levels of TREM2 and HA immunoreactivity were comparable across conditions, confirming a similar expression regardless the presence of the CE (Fig. 7c–f). However, while the DAP12 protein was detected in HEK293 cells transfected with the canonical *Tyrobp* sequence, this was not the case when cells were transfected with CE-containing *Tyrobp* (Fig. 7c,d,g). Taken together, these findings demonstrate that the CE inclusion does not affect overall DAP12 expression but leads to a truncated protein, consistent with our targeted proteomics data (Fig. 6m–o), and that this CE variant is not recognized by antibodies generated against the C-terminal domain (Fig. 7d,g). Given that the DAP12 C terminus is required for intracellular signaling, we then evaluated DAP12-mediated SYK phosphorylation in response to TREM2 ligand activation in transfected HEK cells^29,39^. As expected, while a significant increase in phospho-SYK (pSYK) was observed upon hTREM2 stimulation in cells expressing canonical DAP12, no increase in SYK phosphorylation was measured in cells transfected with CE-*Tyrobp* (Fig. 7h). TREM2 activation assay in cells not expressing HA further confirmed that CE-DAP12 displayed a defective pSYK signaling (Extended Data Fig. 7a), ruling out any potential impact of the HA sequence on DAP12 function. To validate the impaired TREM2-DAP12 signaling in TDP-43 KO microglia, we measured pSYK in primary cultures exposed to myelin and associated lipids, which are known as TREM2 ligands^29^ (Fig. 7i). Consistent with this, we found that, while myelin exposure induced a significant increase in pSYK in controls, this response was abolished in TDP-43 KO microglia (Fig. 7j,k), confirming that the loss of TDP-43 leads to defective SYK signaling. Western blot analysis confirmed a significant reduction in DAP12 in primary microglia lacking TDP-43, as observed in acutely sorted cells (Extended Data Fig. 7b–d and Fig. 6k,l). To further confirm the TREM2-DAP12 axis impairment ex vivo, we acutely isolated microglia from control and cKO mice, and exposed them to the selective TREM2 agonist antibody 4D9 (ref. ^40^) or its isotype control (Fig. 7l). Similarly to what observed in response to myelin, we found that 4D9 treatment induced an increase in pSYK only in control and not in microglia lacking TDP-43 (Fig. 7m,n). However, stimulation of CLEC7A, a receptor that recruits SYK independently of DAP12, led to a significant pSYK response in both control and KO microglia (Extended Data Fig. 7e–g), further demonstrating that the loss of TDP-43 specifically impairs DAP12 function, and not the SYK kinase per se. Altogether, these data demonstrate the specific impairment in the TREM2-DAP12 axis when TDP-43 is depleted in microglia.

Next, we reasoned that if microglial TDP-43 acts upstream of DAP12 and TREM2, causing myelin abnormalities, a similar phenotype would be present in the brains of DAP12-deficient and TREM2-deficient mice. Therefore, we analyzed myelin-bulging events in these mouse strains during early brain development using MBP immunostaining in the deep layer of the motor cortex. In both DAP12 and TREM2-KO animals, we observed a significant increase in the number of MBP^+^ bulging structures (Extended Data Fig. 7h,i,k,l). However, the average size of these myelin abnormalities was not different between KO and control animals (Extended Data Fig. 7h,j,k,m), suggesting that the myelin aberrations observed in TDP-43 cKO mice are not solely due to impaired TREM2-DAP12 signaling, but most likely involve additional mechanisms that have yet to be uncovered.

Phosphatidylserine (PS) has been described as an ‘eat me’ signal for microglia and has been shown to be exposed by aberrant myelin,^28^ as well as an established TREM2 ligand^29,41^. Therefore, we hypothesized that a defective engulfment of myelin due to an impairment in the TREM2-DAP12 signaling would result in the accumulation of externalized PS on myelinated fibers. Injection of PSVue, a fluorescence dye that labels exposed PS, in the brain of P15 control and cKO mice (Extended Data Fig. 7n) revealed an increase in colocalization between PSVue puncta and MBP^+^ structures (Extended Data Fig. 7o,p). Together with the reduced microglial engulfment of myelin observed in cKO mice (Fig. 3), these results support a defective myelin refinement and a consequent accumulation of PS-tagged myelin when microglia lack TDP-43. Overall, these findings reveal a role for TDP-43 in regulating DAP12-mediated signaling, impacting the microglial TREM2-DAP12 axis through CE splicing.

### TDP-43 cKO mice show anxiety-like behavior and motor deficits when depletion is induced early postnatally

**Fig. 8:**
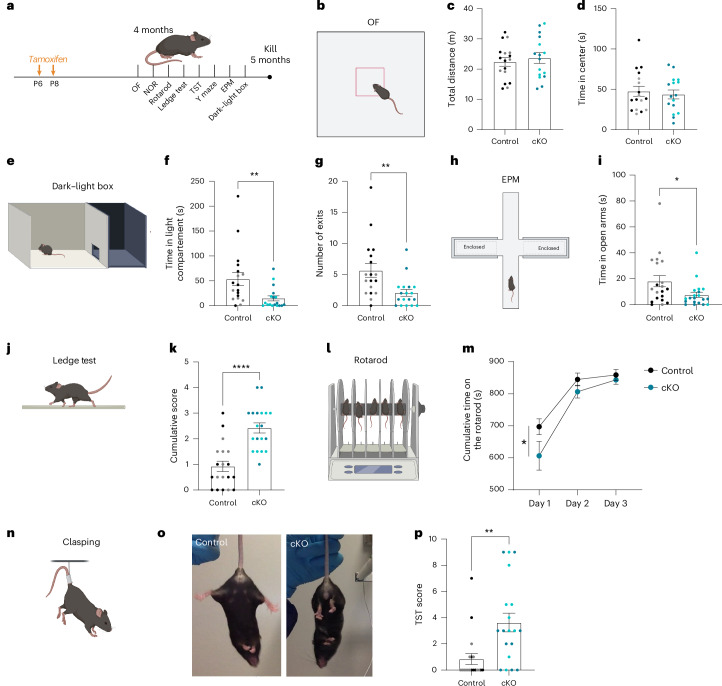
TDP-43 cKO mice show anxiety-like behavior and motor deficits. **a**, Experimental design of behavioral testing on 4-month-old control and TDP-43 cKO mice of both sexes (females in light, males in dark colors). **b**, OF. **c**, Total distance traveled. **d**, Time spent in the center of the arena; *n* = 17 control, *n* = 16 cKO animals. **e**, Dark–light box test. **f**, Total time spent in the light compartment, **P = 0.0090. **g**, Number of exits to the light compartment, **P = 0.0060; *n* = 18 control, *n* = 18 cKO animals. **h**, EPM. **i**, Time spent in the open arms of the apparatus, *P = 0.0329; *n* = 20 control, *n* = 20 cKO animals. **j**, Ledge test. **k**, Cumulative severity score over three trials, ****P < 0.0001; *n* = 19 control, *n* = 19 cKO animals. **l**, Rotarod test. **m**, Cumulative time spent on the rotarod over three trials over 3 days, *P = 0.0379; *n* = 19 control, *n* = 19 cKO animals. **n**, Clasping behavior test. **o**, Representative images of hindlimb reflex in control and cKO animals. **p**, cumulative clasping severity score over three trials, **P = 0.0018; *n* = 19 control, *n* = 19 cKO animals. Statistics in **c**–**k** and **p** represent two-tailed unpaired *t* test; in **m**, two-way ANOVA, Sidak’s multiple comparison test. Data are presented as mean ± s.e.m. EPM, elevated plus maze. Figure created in BioRender; Paolicelli, R. https://biorender.com/cnll417 (2026). Source data

**Extended Data Fig. 8:**
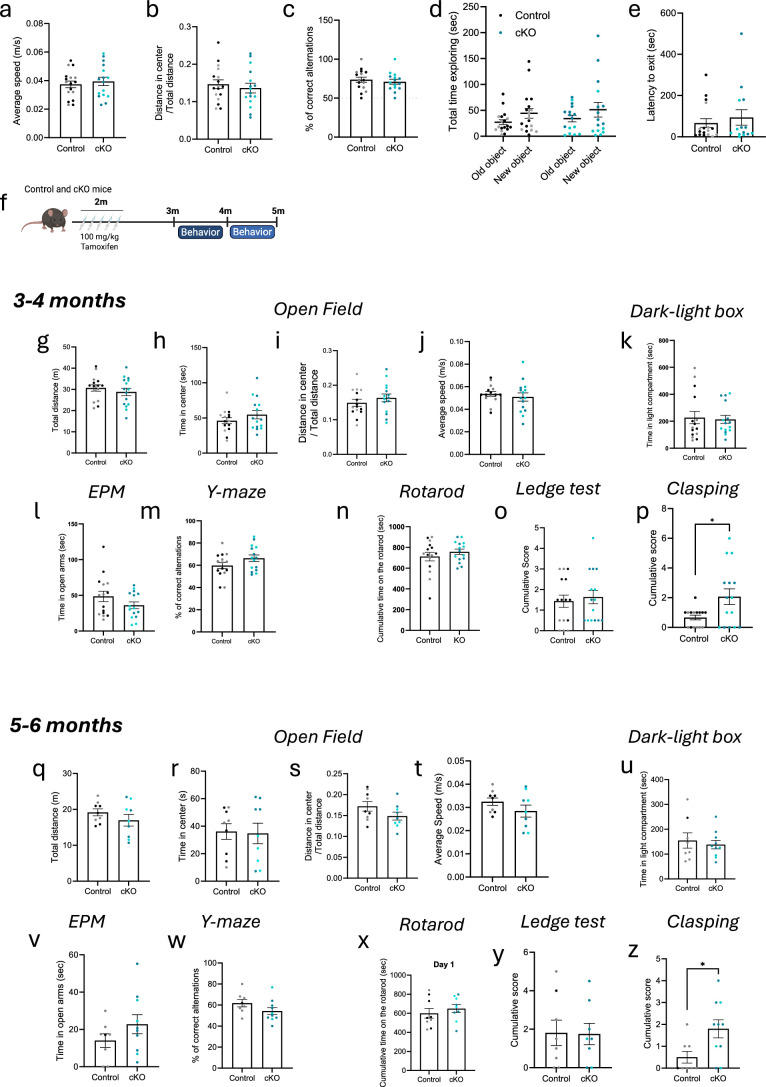
TDP-43 adult cKO displays normal cognitive behavior and late TDP-43 depletion induces mild motor phenotype. **a**,**b**, Quantification of (**a**) average speed and (**b**) distance in the center during the open-field task; n = 17 control and n = 16 cKO mice. **c**, Alternation score in the Y-maze test. n = 17 control and n = 16 cKO mice. **d**, Time spent exploring the objects in the new object recognition task; n = 18 control and n = 16 cKO mice. **e**, Latency to the first exit from the dark to the light compartment in the dark-light test; n = 17 control and n = 14 cKO mice. Females are indicated in light and males in dark colors. **f**, Experimental timeline for late TDP-43 depletion (at 2 months of age) and subsequent behavioral testing. **g**–**z**, Quantification of: (**g**) and (**q**) total distance, (**h**) and (**r**) time in the center, (**i**) and (**s**) distance in the center/tot distance, and (**j**) and (**t**) average speed during the open field task at 3–4 months and 5–6months, respectively. **k**,**u**, Time spent in the light compartment in the dark-light box test. **l**,**v**, Time spent in the open arms during the elevated plus maze test. **m**,**w**, Percentage of correct alternations during the Y-maze. **n**,**x**, Cumulative time spent on the rotarod. **o**,**y**, Cumulative score on the ledge test. **p**, *p = 0.0173, and **z**, *p = 0.0248 cumulative score to quantify hindlimb clasping during tail suspension test. All the test were performed at 3–4 (n =15 control and n = 15 cKO mice) and 5–6 months (n = 8–9 control and n = 9 cKO mice). Statistics in **a**–**z**: two-tailed unpaired t-test. Females are indicated in light and males in dark colors. Stats are performed on animals. Data are presented as mean values ± s.e.m. Source data

Finally, to assess potential behavioral outcomes in TDP-43 cKO mice, we designed a battery of tests covering diverse domains (Fig. 8a). Control and cKO littermates at the age of 4 months displayed no differences in the open-field (OF) test, showing comparable distance traveled, time and distance spent in the center and average speed (Fig. 8b–d and Extended Data Fig. 8a,b), indicating no defects in general locomotion and exploratory behavior. Working and learning memory were assessed by the *Y*-maze test and the new object recognition (NOR) test, respectively, showing no obvious differences between genotypes (Extended Data Fig. 8c,d). However, the dark–light box and elevated plus maze test revealed anxiety-like behavior in cKO mice, evidenced by the decreased time spent in the light compartment and the reduced number of exits (Fig. 8e–g and Extended Data Fig. 8e), and less time spent in the open arms of the maze (Fig. 8h,i). Next, we assessed motor performance using the ledge and rotarod test. cKO mice had significantly impaired ability to walk in the ledge test (Fig. 8j,k and Supplementary Videos 2 and 3), indicating impaired sensorimotor coordination and balance. The rotarod test also revealed a shorter latency to fall for cKO mice, particularly on the first day of testing (Fig. 8l,m). Finally, we evaluated hindlimb clasping behavior as an index of neurological and motor dysfunction and found that cKO mice had higher score compared to controls (Fig. 8n–p). To better dissect the developmental versus constitutive role of microglial TDP-43, we examined a second cohort of mice, in which TDP-43 depletion was induced at 2 months of age, and behavioral testing was performed at 3–4 months and 4–5 months of age (Extended Data Fig. 8f). In contrast to early depletion, late depletion of microglial TDP-43 did not induce any major changes in general locomotion (Extended Data Fig. 8g–j), anxiety-like behavior (Extended Data Fig. 8k–m) and motor behavior (Extended Data Fig. 8o,p). Interestingly, cKO mice show a significant increase in clasping behavior (Extended Data Fig. 8q), suggesting that the loss of microglial TDP-43 is sufficient to induce this neurological phenotype regardless of the age of depletion. These findings show that early loss of microglial TDP-43 induces anxiety-like behavior and motor deficits, while late depletion only induces hindlimb clasping behavior in young adult mice. Overall, loss of microglial TDP-43 is sufficient to induce motor alterations, particularly when the depletion occurs early postnatally.

## Discussion

TDP-43 pathology is a key component of diverse neurodegenerative diseases, including ALS and FTD, and is extending to AD, and limbic predominant age-related TDP-43 encephalopathy^2,3,42^. While there has been a strong focus on TDP-43 in neurons, little attention has been given so far to glial cells. In this study, we have characterized the early consequences of TDP-43 depletion in microglia on brain development. Using an inducible cKO mouse model, we deleted the *Tardbp* gene in CX3CR1^+^ cells and assessed brain-wide macrostructural consequences using diffusion MRI. Remarkably, MRI analysis revealed differences in RD within a localized brain region, including the RSC/MC, with no evident alterations elsewhere in the brain. Diffusion MRI is often used to assess microstructural and myelin damages, and RD has been frequently linked to myelin defects in a range of neurological disorders^27^. These findings prompted us to further investigate the cellular and molecular correlates in this region, focusing on myelin and OLs. Consistent with literature reporting that myelin biogenesis is error prone and subjected to substantial refinements in the early postnatal brain^28^, we found that juvenile mice, at 2 weeks of age, presented a collection of myelin abnormalities. FireΔ/Δ mice and mutant Csf1r zebrafish larvae, which lack microglia, were previously shown to display exacerbation of aberrant myelin structures, indicating that microglia actively participate in resolving these abnormalities^28,43^. We observed similar outcomes, suggesting that microglial TDP-43 can regulate, at least partially, key molecular mechanisms responsible for microglia-mediated myelin refinement. In line with this, functional experiments showed that TDP-43 KO microglia are impaired in both myelin engulfment and digestion. It is also likely that the myelin phenotype is exacerbated by the overall reduction in microglial density induced by TDP-43 depletion.

Microglia have been shown to influence the maturation of the OL lineage^44,45^, with depletion of microglia at early postnatal stages resulting in decrease of PDGFRα^+^ OPCs number at P8 (ref. ^45^). We observed a significant reduction in the pool of PDGFRα^+^ OPCs at P15, suggesting that the selective loss of TDP-43 in microglia may phenocopy the effects of microglia depletion on OPCs. The reduction in the OPC pool may be due to decreased proliferation, as supported by reduced Ki67 staining. On the other hand, we found that the pool of mature CC1^+^ OLs at P15 was significantly increased in cKO mice. It is plausible that the accelerated myelination and the overall increase in myelin contribute to the emergence of bulging structures observed in cKO mice. Notably, in vitro experiments coculturing WT OPCs with either control or KO microglia recapitulated the phenotype observed in vivo, with KO microglia inducing reduced proliferation of OPCs. Furthermore, we found that the conditioned medium from KO microglia was sufficient to increase MBP production in CC1^+^ cells. While additional investigations of the underlying mechanism are required, these findings support an imbalance between mature and immature OLs, consistent with a shift toward mature OLs and concomitant exhaustion of the progenitor pool. The mitochondrial abnormalities in the OLs of cKO mice might be in line with this possibility, further supporting a metabolic stress phenotype. It is important to highlight that the microglia-mediated effects on OLs were only observed in the RSC/MC, whereas the number of OLIG2^+^ cells in the hippocampus and barrel cortex remained comparable between genotypes. Moreover, while the OPCs/OLs imbalance observed in the motor region at P15 was not present yet at P12, it persisted into young adulthood and was particularly prominent in the corpus callosum.

Spatial transcriptomic data indicated enhanced antigen-presenting capacity and the emergence of an IFN-responsive signature. The increase in genes associated with the MHC protein complex, predominantly in the RSC/MC area, was not detected in acutely isolated microglia. On the contrary, the transcriptomic profile of microglia sorted from the motor/SSC revealed a significant decrease in MHC-related proteins. It was recently shown that, in a model of experimental autoimmune encephalopathy, OL lineage populations express genes involved in antigen processing and presentation through MHC-I and MHC-II, perform phagocytosis and efficiently activate T cells^46^. Thus, it is plausible that, in the context of microglial TDP-43 depletion and the loss of antigen-presenting capacity, OLs could take over these alternative functions. It will be important to further validate this hypothesis by specific functional assays. Another prominent feature that emerged from spatial transcriptomics was a general IFN-responsive signature, upregulated specifically in the RSC/MC areas and not in the hippocampus, confirmed to be increased in OLs. This expression pattern appeared similar to a previously described signature of diseased OL/OPCs in a preclinical model of multiple sclerosis^46^ and to an OL signature associated with IFN (IFN-responsive oligodendrocyte) enriched in the aging white matter^47^. In pathological contexts, such as demyelination, IFN has been shown to reduce the number of OPCs and induce MHC protein complexes^48^, and an IFN-responsive oligodendrocyte signature has been described in experimental autoimmune encephalopathy and aging^46,47^.

One of the major roles of TDP-43 is to regulate mRNA splicing and to control the expression of CE^1,9^. The set of exons differentially spliced by TDP-43 is highly dependent on the cellular identity, impacting distinct molecular pathways according to the cell type in which TDP-43 is dysregulated^10^. Previous work has identified specific TDP-43-dependent targets in embryonic stem cells^9^, motor neurons^13^, skeletal myocytes^49^ or brain pericytes^50^. The TDP-43-dependent regulation of CE in microglia, however, has never been explored so far, despite the significant expression of TDP-43 in these cells. Here using DEXSeq^51^ and MAJIQ V3 (ref. ^33^), we identified a converging list of 42 genes as spliced targets in microglia, among which the *Tyrobp* gene. *Tyrobp* encodes DAP12, a short polypeptide of only 113–114 amino acids, consisting of a short extracellular tail, a single transmembrane domain and a cytoplasmic immunoreceptor tyrosine-based activation motif^52^. More than 20 different surface receptors have been described to associate with DAP12 for signal transduction, including TREM2, SIRPb, CLEC5A, SIGLECH and others^52^. Several signaling pathways converge to DAP12 activation and subsequent SYK phosphorylation; therefore, DAP12 dysfunction is likely to result in a complex phenotype. Loss of DAP12 function is disease linked, as homozygous *TYROBP* mutations, similar to *TREM2* mutations, cause Nasu–Hakola disease, associated with white matter anomalies, bone cysts, as well as early-onset dementia^53^. Of note, *Tyrobp* expression was reported to be upregulated presymptomatically and before *Trem2* in a mouse model of ALS, and upregulated in ‘postmortem’ ALS samples, highlighting a dysregulation of the TREM2-DAP12 axis at early stages of the disease^54^. Here we reported that the CE-containing *Tyrobp* mRNA produced when TDP-43 is KO results in a truncated DAP12 protein. We were able to detect the CE-DAP12 by mass spectrometry, particularly when protein degradation was inhibited, confirming that truncated DAP12 is promptly degraded. CE-DAP12 lacks the C-terminal domain, and it is unable to elicit pSYK signaling upon TREM2 activation, as shown both in TREM2-competent HEK cells and in microglia exposed to selective TREM2 agonist antibody and myelin. It is important to note that the PSI of the identified *Tyrobp* CE is relatively low (11–14%; Supplementary Table 3). However, given the high expression of *Tyrobp* in microglia, the resulting percentage of transcripts containing CE could still have a biological relevance. Overall, it remains to be clarified whether CE insertion is sufficient to induce defective TREM2-DAP12 signaling or whether additional mechanisms are involved.

TREM2-mediated recognition of myelin could be impaired in TDP-43 KO microglia when DAP12 is defective in signal transduction. Consistent with this hypothesis, we found that aberrant myelin structures were also significantly more frequent in the motor cortex of TREM2 KO and DAP12 KO mice. However, in these mice, the bulging structures had comparable sizes, suggesting that additional mechanisms contribute to myelin abnormalities in TDP-43 cKO mice.

When subjected to a comprehensive behavioral battery, young adult cKO mice in which depletion was induced at P6–P8 displayed defective motor behavior, showing hindlimb clasping, poor motor balance and coordination. Interestingly, cKO mice also displayed increased anxiety-like behavior. This may be consistent with MRI microstructural alterations in the anterior cortex of cKO mice, a brain structure associated with anxiety^55^. However, when depletion was induced at 2 months of age, only the hindlimb clasping phenotype was evident, supporting a key role of microglial TDP-43 for motor function, as clasping is commonly described in mouse models with deficits in cerebello-cortico-reticular pathways^56^. The anxiety-like behavior observed after early depletion was no longer visible, highlighting the importance of microglial TDP-43 in critical developmental windows. Further experiments are warranted to investigate more in depth the cellular mechanisms underlying these behaviors and the differences due to the timing of depletion.

Overall, here we found that microglial TDP-43 influences the ratio between OPCs and mature OLs, controls myelin refinement and preserves microstructural integrity of the RSC/MC in the early postnatal mouse brain. Early loss of TDP-43 selectively in microglia was sufficient to induce motor dysfunction in young adult mice. Finally, we reported a link between TDP-43 loss of function and DAP12 signaling, proposing a cellular mechanism whereby microglial TDP-43 contributes to the regulation of the TREM2-DAP12 axis.

### Limitations of the study

While our study highlights a role for microglial TDP-43 in controlling microglial function and influencing OLs and myelin maturation, it is important to acknowledge several limitations. First, TDP-43 is a master regulator of gene transcription and splicing, with hundreds of genes differentially regulated in microglia following its depletion. Therefore, it is likely that multiple parallel pathways contribute to TDP-43-mediated microglial functions, and further investigation is needed to explore the implications of those pathways. Moreover, this work only addresses the loss of microglial TDP-43 in the healthy brain, and further studies are necessary to investigate its role in models of motor neuron dysfunction. Second, our study focused on the RSC/MC, guided by unbiased DTI MRI findings that revealed specific microstructural alterations in this region. However, it remains unclear why the observed phenotype is restricted to this localized area, despite effective microglial TDP-43 depletion throughout the brain. Although we have shown that the OPC/OL imbalance is restricted to the RSC/MC and not observed in the barrel cortex or hippocampus, further research is required to examine other regions specifically involved in motor behavior, such as the cerebellum and spinal cord. Third, we limited our functional analysis of CEs to the *Tyrobp* gene due to its central importance in microglial biology. However, other target genes regulated by TDP-43-mediated splicing in microglia may also have critical roles and several of those such as *Synj2bp*^57^, *Fam21* (ref. ^58^), *Tnfaip1* (ref. ^59^) have been previously linked to motor neuron diseases. Therefore, future studies are needed to investigate their potential functional implications.

Finally, and most critically, our findings were derived from work in mice, and further research is necessary to determine whether these mechanisms are conserved in humans. While TDP-43 itself is highly conserved across species^60^, its target genes may not necessarily be, therefore investigating the role of TDP-43 in human microglia is of utmost importance to understand its relevance in disease. Recent studies using monocyte-derived microglia-like cells from patients with sporadic ALS have shown nonphosphorylated and phosphorylated TDP-43-positive inclusions, supporting the need to investigate the consequences of TDP-43 mislocalization in human microglia^61^. Altogether, these limitations underscore the urgency for additional studies to fully understand the complexity of TDP-43 role in regulating microglial function and its broader implications for brain development and disease.

## Methods

### Animals

All animal experiments were authorized by the Service de la consommation et des Affaires vétérinaires of the Canton de Vaud in Switzerland, and by the Direction générale de l’agriculture, de la viticulture et des affaires vétérinaires (DGAV), Affaires vétérinaires Protection des animaux, under the animal licenses VD3868 and VD3491. Mice were bred and maintained in the animal facility of the University of Lausanne. Cx3cr1-CreERT2;*Tardbp*^*fl*^ mice were obtained by crossing the B6.129P2(Cg)-Cx3cr1tm2.1(cre/ERT2)Litt/WganJ mice (Jackson Laboratory, 021160) with and B6(SJL)-Tardbptm1.1Pcw/J (Jackson Laboratory, 017591). The Cx3cr1-CreERT2;*Tardbp*^*fl*^ line was further crossed with B6.Cg-Gt(ROSA)26Sortm14(CAG-tdTomato)Hze/J mice (Jackson Laboratory, 007914). All the experimental subjects were obtained by crossing Cx3cr1-CreERT2/creERT2;*Tardbp*^*fl/WT*^ with *TdTomato*^*fl/fl*^;*Tardbp*^*fl/WT*^ to produce littermates that were all heterozygous for Cx3cr1-CreERT2 and tdTomato, and WT or homozygous (cKO) for the Tardbp-floxed allele. Mice were group housed and kept in a 12-h day/12-h night cycle, with food and water ad libitum, at 20–22 °C. All the experiments were performed during the day cycle. Both male and female mice were analyzed, unless otherwise specified. For in vivo KO induction, tamoxifen (Sigma-Aldrich, T5648) was prepared in 10% ethanol in corn oil at a concentration of 6 mg ml^−1^. Mice were injected intraperitoneally (i.p.) at P6 and P8 (75 mg kg^−1^). Late KO induction was achieved by injecting i.p. 200 μl of tamoxifen (dissolved in 10% EtOH and 90% corn oil at 10 mg ml^−1^) for five consecutive days in 2-month-old mice. For biochemical and histological assessments, mice were terminally anesthetized with sodium pentobarbital diluted in saline (150 mg kg^−1^, i.p.) and perfused with cold HBSS (Life Technologies, 14175129; 3 ml min^−1^). Upon brain collection, the left hemisphere was postfixed in cold PFA 4% for 24 h and stored at 4 °C in 0.02% NaN3 in PBS. The cortex and hippocampus were dissected from the right hemisphere, immediately frozen in dry ice and stored at −80 °C.

### Primary microglia culture

Brains from P3–P5 pups were collected and cerebellum, meninges and olfactory bulbs were removed. Both the hemispheres were mechanically shattered with forceps and put into 3 ml of TrypLE Express Enzyme Solution (Thermo Fisher Scientific, 12-604-021) at 37 °C for 20 min. The tissue was further dissociated by pipetting with a 1 ml tip. Enzymatic reaction was stopped by adding 10 ml of culture medium (DMEM 4.5 g l^−1^ of glucose, 10% FBS and 1% penicillin–streptomycin; Thermo Fisher Scientific, 11995073, 10500064 and 15140122). Cells were pelleted at 400*g* for 4 min at room temperature and the pellet resuspended in fresh culture medium. Cells obtained from each individual brain were seeded into a T75 cell culture flask. Cultures were maintained at 37 °C and 5% CO_2_. After 2 weeks in culture, microglia were collected by smacking the flask. Cells were pelleted at 350*g* for 5 min and the astrocyte-conditioned media (ACM) were collected. Pellet was resuspended in ACM and the cells were seeded (20,000 cells per well of a 96-well plate) on a coating of poly-D-lysine (0.1 mg ml^−1^, 1 h at 37 °C; Sigma-Aldrich, P7886). Microglial cells were kept in ACM and the following treatments were performed in ACM.

### KO induction in vitro

Here 4-hydroxytamoxifen (Sigma-Aldrich, H7904; 500 μM in 2.5% ethanol in PBS) was diluted in ACM to a concentration of 500 nM and was applied onto primary microglia cultures, with medium change after 24 h. Imaging-based assays or cell lysis were performed after 6–7 days.

### In vitro myelin assays: myelin debris preparation and pHrodo conjugation

Two-month-old C57Bl/6 mice were anesthetized and perfused intracardially with ice-cold HBSS. Tissue was processed according to the MACS Neural Tissue Dissociation Kit (P) (Miltenyi Biotec, 130-092-628) instructions. Brains were dissected, and olfactory bulbs and cerebellum were removed. Tissue was grossly cut in smaller pieces and added to Miltenyi gentleMACS C Tubes. Samples were placed in the Miltenyi gentleMACS Dissociator, and the mBrain_01_02_03 protocols were run according to the manufacturer’s instructions. Samples were then briefly spun before being filtered through 70-μm filter. Samples were washed with HBSS and spun to pellet cells. For myelin isolation, cell pellets were resuspended, supplemented with an appropriate volume of Myelin Removal Beads II (Miltenyi Biotec, 130-096-731) according to Miltenyi’s protocol. Samples were resuspended in 1ml and passed through an LS column (Miltenyi Biotec, 130-042-401) attached to a MACS Separator (Miltenyi Biotec, 130-091-051). After washing, the column was removed from the magnetic rack and the labeled myelin was flushed out in 3 ml of buffer. Samples were centrifuged at 4,000*g* for 10 min at 4 °C, further washed with 1 ml ice-cold PBS1X and resuspended in 250 μl of PBS1X.

Protein concentration was determined through BCA Protein Assay (Thermo Fisher Scientific, 23227) following the manufacturer’s instructions. Nine microliters of pHrodo-Green (Thermo Fisher Scientific, P36012) was added to 100 μg of myelin and supplemented with 1/10 of sodium bicarbonate 1M (10 μl for 100 μl). Samples were incubated for 45 min in rotation and covered from light. Samples were further centrifuged at 4,000*g* for 10 min at 4 °C and resuspended in PBS to have a final stock concentration of ≈1.5 μg μl^−1^. Unlabeled and labeled myelin aliquots were kept at −80 °C.

### Phagocytic assay

Primary microglia were incubated with 15 μg ml^−1^ pHrodo-myelin for 4 h. After four PBS1X washes, cells were fixed with 4% PFA (20 min, room temperature) to evaluate the amount of the cargo that was taken up (T0) or incubated with fresh medium for 24 h (T24), before washes and fixation, to assess myelin degradation.

### Myelin engulfment assay by FC

After MACS isolation of microglia from P15 control or cKO pups, microglia were incubated with 15 µg of pHrodo-labeled myelin for 4 h at 37 °C and 5% CO_2_. After incubation, cells were washed and resuspended in MACS buffer with DAPI (1:1,000) as a live/dead marker. TdTomato^+^ microglia were gated, and myelin uptake was quantified by FC based on the geometric mean fluorescence intensity of pHrodo and the number of live TdTomato^+^ pHrodo^+^ cells.

### pSYK assay

Primary microglia were incubated with 15 μg ml^−1^ pHrodo-myelin for 5 min. Cells were washed with 1× PBS and lysed in RIPA buffer supplemented with protease/phosphatase inhibitors (PPI) cocktail (Thermo Fisher Scientific, A32961). After 30 min on ice, samples were stored at −80 °C.

### TREM2 agonist treatment

Acutely MACS-sorted microglia were seeded and treated with 4D9 TREM2 agonist and 4D5 isotype antibodies (20 µg ml^−1^) for 5 min. Cells were rapidly washed with 1× PBS and lysed in RIPA buffer supplemented with PPI cocktail (Thermo Fisher Scientific, A32961). After 30 min on ice, samples were stored at −80 °C.

Curdlan (β-1,3-glucan) powder was suspended in sterile water and heated at 80 °C for 30 min to form a homogeneous aggregate suspension, then cooled and used at a final concentration of 100 µg ml^−1^ for in vitro stimulation experiments. Cells were treated for 30 min to assess SYK phosphorylation.

### Immunofluorescence

#### Brain slices

Thus, 60-µm coronal brain slices were cut using a vibratome VT1200S (Leica Biosystems) and stored in PBS containing 0.02% sodium azide at 4 °C until use. For PSVue injection experiments, sections were treated for 7 min with Sudan Black (Sigma-Aldrich, 199664) at a concentration of 0.1% in 70% ethanol and then thoroughly washed to reduce tissue autofluorescence and background, while preserving the specific fluorescence signal. Slices were permeabilized in 0.5% Triton X-100 in PBS for 90 min at room temperature, followed by incubation with blocking buffer (2% BSA in permeabilization buffer) for 1 h at room temperature. Primary antibodies were diluted in blocking buffer and incubated overnight at 4 °C with mild agitation. Brain slices were then washed in PBS and further incubated with fluorescently labeled secondary antibodies (1:1,000 in blocking buffer) for 2 h at room temperature. After additional washes in PBS, nuclei were stained for 15 min at room temperature with DAPI (1 μg ml^−1^ in PBS; Thermo Fisher Scientific, D1306). Finally, slices were mounted on microscope slides using Mowiol 4-88 (Sigma-Aldrich, 81381).

#### Cells

Cells were washed with PBS, fixed (PFA 4%, room temperature, 20 min) and permeabilized in 0.25% Triton X-100 in PBS for 15 min at room temperature. Blocking was performed with 2% BSA in permeabilization buffer (30 min at room temperature). Primary antibodies were diluted in blocking buffer and incubated overnight at 4 °C. Cells were washed with PBS and incubated with fluorescently labeled secondary antibodies (1:1,000 in blocking buffer) for 1 h at room temperature. Cells were washed thrice with PBS and nuclei were stained for 5 min at room temperature with DAPI (1 μg ml^−1^ in PBS). Coverslides were mounted on microscope slides using Mowiol 4-88.

#### Antibodies

The following antibodies were used: MBP (Proteintech, 10458-1-AP; 1:400), OLIG2 (Merck Millipore, AB9610; 1:400), CC1/APC (anti-APC (Ab-7) mouse mAb (CC1); Merck Millipore, OP80; 1:200), PDGFRα (CD140a; Merck Millipore, CBL1366; 1:400), CD68 (Bio-Rad, MCA1957; 1:400), TDP-43 (Proteintech, 10782-2-AP; 1:100), NF200 (Bio-Techne Sales, NB300-217; 1:80), KI67 (Invitrogen, 14-5698-82; 1:300; Abcam, ab15580; 1:1,000) and Stat1 (Cell Signaling Technology, 14994S; 1:400).

### Confocal microscopy and image analysis

Acquisitions were taken with a Stellaris 5 confocal microscope (Leica Biosystems), with a ×20 dry (*z* stack, step size = 0.68 µm) or ×63 immersion objective (*z* stack, step size = 0.3 μm), with digital zoom. Confocal acquisitions were processed using ImageJ Software or Imaris Software (Fiji, Bitplane).

#### Density analysis

In 10 µm or 20 μm *z*-stack images, DAPI and cell of interest (OLIG2^+^ or tdTomato^+^ cells) were thresholded using identical settings within each experiment. The two channels were then ‘multiplied’ using the Image Calculator tool in Fiji. The result window was then *z* projected, and the number of cells was quantified using the Analyze Particles tool in Fiji. For CC1^+^ and PDGFRα^+^ cells, projected 10 μm *z*-stack images were manually quantified, blinded to the experimenter, using the Cell Counter tool in Fiji.

#### Bulging structures

Ultrastructural myelin aberrations were quantified in *z*-stack confocal acquisitions using the Cell Counter tool in Fiji. Single plan images of the largest bulging section were used to trace the circumference and quantify the area of bulging structures in Fiji.

#### Coherency analysis for MBP^+^ myelinated fibers

Coherency of myelinated axons was assessed using the ‘OrientationJ measure’ function of ImageJ plugin OrientationJ in the selected whole micrograph^62^.

#### Microglial morphometric analysis and myelin engulfment

The 3D reconstruction was performed using Imaris software (Bitplane), with the built-in ‘surface’ module. tdTomato (microglia), CD68 and MBP volumes were quantified by applying 3D surface rendering of confocal stacks in their respective channels, using identical settings per channel (fixed thresholds of intensity and voxel) within each experiment. For quantification of MBP engulfment by microglia, only MBP puncta present within microglial CD68^+^ structures were considered. Using the ‘Mask’ function in Imaris, MBP signal within CD68^+^ structures was reconstructed and quantified as previously described in refs. ^8,63^.

#### Filament tracer

Imaris filament-tracing plugin was used. Starting points were identified, with the largest soma diameter set to 12 µm and the smallest dendrite diameter to 0.6 µm. Disconnected segments were removed. Fixed thresholds between conditions were applied. Analysis of the number of segments, the number of end points and the total filament length per cell was performed.

#### PSVue reconstruction analysis

Furthermore, 3D reconstruction was performed using Imaris Software (Bitplane), with the built-in ‘surface’ module. MBP and PSVue volumes were quantified by applying 3D surface rendering of confocal stacks in their respective channels, using identical settings. Using the ‘Mask’ function in Imaris, we reconstructed PSVue signal within MBP structures.

### Western blot

Cells and brain tissue were lysed in ice-cold 1× RIPA (Merck Millipore, 20-188), supplemented with protease inhibitors (Sigma-Aldrich, 11836170001) or PPI (Thermo Fisher Scientific, A32961) depending on experimental needs. Samples were spun at 12,000*g* for 15 min at 4 °C. The supernatant was collected, and protein concentration was determined through BCA Protein Assay (Thermo Fisher Scientific, 23227) following the manufacturer’s instructions. Next, 5–30 μg of protein were adjusted to equal sample volume using RIPA buffer protease inhibitors or PPI and the same amount of 4× protein loading buffer was added (LI-COR Biosciences, 928-40004). Depending on datasheet indication, samples were boiled or not at 95 °C for 5 min before gel loading. Proteins were separated on precast 4–15% Mini-PROTEAN TGX Gels (Bio-Rad, 4561086 and 4561084) and transferred onto nitrocellulose membranes (Trans-Blot Turbo Midi NC Transfer; Bio-Rad, 170-4159) using a semi-dry transfer protocol. Before blocking, the total amount of protein per lane was determined using REVERT Total Protein Stain Solution (LI-COR Biosciences, 926-11011) and used for normalization. Blocking was performed in 3% BSA in TBST (0.02 M Tris base, 0.15 M NaCl, 0.05% Tween-20 in milliQ water, pH 7.2–7.4) for 1 h at room temperature and incubated overnight at 4 °C with primary antibodies diluted in blocking buffer. Membranes were washed thrice with TBST for 5 min at room temperature and incubated with secondary antibodies (IRDye, 1:15,000 in blocking buffer; LI-COR Biosciences). After washing thrice with TBST, membranes were imaged with the LI-COR Odyssey system. Uncropped blots are provided with this paper as source data.

#### Antibodies

The following antibodies were used: OLIG2 (Merck Millipore, AB9610; 1:3,500), MBP (Proteintech, 10458-1-AP; 1:2,000), TDP-43 (Proteintech, 10782-2-AP; 1:1,000), IBA1 (Sigma-Aldrich, ZMS1024; 1:1,000), GFAP (Sigma-Aldrich, G3893; 1:1,000), SYK (Cell Signaling Technology, 13198; 1:1,000), pSYK (Cell Signaling Technology, 2717; 1:1,000), STAT1 (Cell Signaling Technology, 14994T; 1:1,000), DAP12 (Abcam, ab283679; 1:1,000), actin (Abcam, ab8227; 1:5,000), GAPDH (Proteintech, 60004-1-Ig; 1:5,000) and ubiquitin (Sigma-Aldrich, 662099; 1:1,000).

### Spatial Visium RNA-seq 10x Genomics

#### Tissue processing

P15 pups were perfused with ice-cold 1× HBSS and brains were collected and postfixed in 10% neutrally buffered formalin for 24 h at 4 °C. Brains were rinsed in 1× PBS and embedded in paraffin and placed in formalin-fixed paraffin-embedded (FFPE) blocks. RNA integrity of tissue samples in FFPE blocks was first ensured by extracting RNA with the RNeasy FFPE kit (Qiagen, 73504), and RNA integrity number was evaluated using RNA ScreenTape (Agilent, 5067-5576) on the 4150 TapeStation System (Agilent). The tissues were then processed according to the manufacturer’s instructions. Briefly, 5 µm microtome sections were placed on Visium Spatial Gene Expression slides (10x Genomics, 2000233) after floating on a water bath at 43 °C. After sectioning, the slides were dried vertically at 40 °C in a hybridization oven for 1 h and 45 min. The slides were then placed inside a box with desiccant pearl, sealed with parafilm and left overnight in a refrigerator at 4 °C. The slides were deparaffinized and hematoxylin and eosine stained according to the manufacturer’s instructions. Hematoxylin and eosin images were acquired with a Leica DMi8 inverted microscope equipped with a Leica DFC7000T color camera and a ×10 objective. Sections were then decrosslinked, processed for probe ligation using Visium Mouse Transcriptome Probe Kit, Small PN-1000365 and preparation of cDNA libraries. cDNA libraries were prepared according to the manufacturer’s protocol (https://www.10xgenomics.com/support/spatial-gene-expression-ffpe/documentation/steps/library-construction). To verify the size of PCR-enriched fragments, we checked the template size distribution by running a D1000 ScreenTape (Agilent, 5067-5582) on 4150 TapeStation System (Agilent). Sequencing was done on BGI’s DNBseq platform (BGISEQ-500/DNBSEQ-G50 sequencing), run in paired-end, dual index configuration with a read length of 28 bp for read 1 (spatial barcode and UMI), 10 bp index read (i7 index), 10 bp index read (i5 index) and 50 bp for read 2 (RNA read).

#### Data analysis

Initial processing of the 10x Visium data was done with spaceranger (v1.3.1) using refdata-gex-mm10-2020-A as a transcriptome reference. The downstream analysis was carried out with R Seurat package (v4.1.1). The raw gene expression data were normalized, using sctransform, and then subjected to PCA using the ‘RunPCA‘ function with default parameters. The top 30 principal components were used to identify nearest neighbors among the cells using the function ‘FindNeighbors’. Based on the identified neighbors, cells were clustered using the Louvain algorithm through the ‘FindClusters’ function. The cell-to-cell relationships were visualized in two dimensions using the Uniform Manifold Approximation and Projection algorithm. This was performed using the ‘RunUMAP’ function with the top 30 principal components. For each cluster, the expression values were aggregated to create a pseudobulk count data. Differential gene expression analysis for each cluster was performed using DESeq2 (ref. ^64^).

### Sample preparation and DTI

Mice were anesthetized and subjected to intracardiac perfusion for approximately 5 min (30 ml, 6 ml min^−1^) with PBS containing 10 mM of ProHance. An additional 15 min perfusion (ca. 100 ml) was performed with a solution of PFA (4%) + prohance (1 mM) + sodium azide 0.01%. The mouse head was chopped off and all extra-cranial tissues removed, leaving the brain intact in the skull. The samples were placed into test tubes, filled with Fomblin solution and degassed (lid open) for 30 min to allow air bubbles to emerge. The tube was then filled with fomblin, tightly screwed and stored at 4 °C until MRI acquisition. Data were acquired on a 7 T Bruker BioSpec scanner equipped with a Pharmascan magnet and a high signal-to-noise ratio dedicated mouse brain quadrature T/R cryogenic coil (Bruker BioSpin; gradient—7 T, 16-cm bore size, 9-cm head gradient insert, maximum gradient 770 mT m^−2^, rise time 110 µ s, AVANCE III electronics). Standard adjustments included calibration of the reference frequency, cryogenic coil power and magnetic field homogeneity optimization (shimming) using MAPSHIM (Paravision; v6.1). For anatomical assessment, a T1-weighted image was acquired through a FLASH sequence with an in-plane resolution of 0.05 × 0.02 mm^2^, an echo time (TE) of 3.51 ms and a repetition time (TR) of 522 ms. For the diffusion-weighted MRI, a high-resolution multishot spin-echo echo-planar imaging sequence with four segments was used to acquire diffusion-weighted images along with 90 encoding directions and *b* values of 0, 1,000, 3,000 s mm^−^^2^. Other parameters were TR = 2000 ms, TE = 22 ms, in-plane RES = 0.2 × 0.2 mm^2^, slice thickness = 0.4 mm, for a total scan time of ≈8 h.

After individual realignment of diffusion images, eddy current correction and tensor estimation were applied (see ref. ^65^ for details). Fractional anisotropy, MD, RD and main diffusivity (Y1) maps were then generated. The resulting volumes were spatially normalized to a study-specific template using Advanced Normalization Tools (http://picsl.upenn.edu/software/ants/, RRID:SCR_004757). Statistics were performed using FSL (Analysis Group, FMRIB, RRID:SCR_002823).

### EM, tissue processing and imaging

Brain sections were processed for scanning electron microscopy (SEM). Coronal sections containing the cortex (Bregma −0.94 mm to −1.82 mm, Paxinos) were selected. Sections were washed with PBS five times for 5 min. Samples were postfixed with 3.5% acrolein in PBS for 2 h. Samples were postfixed in osmium-thiocarbohydrazide-osmium. Sections were incubated in a 1:1 solution of 4% aqueous osmium tetroxide (Electron Microscopy Sciences, 19190) and 3% potassium ferrocyanide (Millipore Sigma, P9387) in double distilled (dd)H_2_O for 1 h. Sections were washed thrice with ddH_2_O for 5 min and incubated in 1% thiocarbohydrazide (Millipore Sigma, 223220) and diluted in ddH_2_O for 20 min. After washing the sections thrice for 5 min, they were incubated for 30 min in 2% osmium tetroxide diluted in ddH_2_O and then dehydrated in ascending concentrations of ethanol (twice in 35%, one time in 50%, 70%, 80%, 90%, and thrice in 100%) followed by three incubations of 5 min in propylene oxide. After dehydration, the sections were flat embedded in Durcupan ACM resin (Millipore Sigma, 44610). Briefly, the sections infiltrated the resin at room temperature overnight. They were carefully placed on a fine layer of resin between two sheets of ACLAR embedding films (Electron Microscopy Sciences, 50425) for polymerization at 55 °C for 72 h. After polymerization, a section containing the region of interest was excised and glued to a Durcupan resin block for ultrathin sectioning (Ultracut UC7 ultramicrotome, Leica Biosystems). Ultrathin sections, of ~75-nm thickness, were collected on a silicon nitride chip and placed on specimen mounts for SEM. Images of our cells of interest and myelin structures were imaged at a resolution of 5 nm per pixel using a Zeiss Crossbeam 350 SEM running ATLAS 5 software.

#### Ultrastructural identification

Microglial cell bodies were identified by their dark irregular cytoplasm, heterogeneous chromatin pattern, distinctive long stretches of endoplasmic reticulum and lipidic inclusions (that is, lipofuscin, lipid bodies or droplets, lysosomes), as well as frequent contacts with axon terminals^31^. OLs were identified by their squarish cell body, common presence of two or more Golgi apparatus, having most of the cytoplasm on a single side, and by the common contact with myelinated axons, often being able to see the membrane directly feeding the myelin of the axon^66^. Organelles were identified by their ultrastructural features^31,66^. Mitochondria with signs of cellular stress were considered and analyzed as described in ref. ^31^. In detail, mitochondrial abnormalities were quantified based on electron-dense inclusion/granules (dense deposits within the matrix), fragmented mitochondria, mitochondrial vacuolization (the presence of clear vacuoles within the mitochondrial matrix) and cristae disorganization. Myelin alterations were identified and described—‘redundant myelin’ was considered when myelin is not surrounding an axon and it coils on itself; ‘internalized myelin’ was identified as portions of myelin that appeared to be internalized into the axon.

#### *G*-ratio analysis

Myelin ultrastructure was quantified using MyelTracer, a semi-automated segmentation tool optimized for EM images^67^. Transverse sections of the deep layer of the cortex were imported into MyelTracer, and individual axons were semi-automatically segmented. For each fiber, the inner axonal diameter and total fiber diameter and *g* ratio were extracted. Myelin sheath thickness was calculated as the difference between the total and axonal diameters. Inner tongue thickness was measured as the distance between the axon diameter and the innermost myelin wrap. All segmentations were visually inspected and manually corrected when necessary to ensure accuracy. At least *n* = 65 fibers per animal were analyzed, and the data were exported for statistical analysis. All analyses were performed with the experimenter blinded to genotype and treatment.

### Expansion microscopy

Protein-retained expansion microscopy was performed following Basic Protocol 2 from ref. ^68^ with minor modifications. Briefly, fixed tissue samples were permeabilized and incubated with primary antibodies against TdTomato and MBP (Proteintech, 10458-1-AP; 1:400). After washing, samples were incubated with appropriate fluorophore-conjugated secondary antibodies, followed by anchoring in 0.1–0.2 mg ml^−1^ acryloyl-X, SE in PBS. Gels were polymerized using a monomer solution consisting of 8.6% (wt/vol) sodium acrylate, 2.5% (wt/vol) acrylamide and 0.15% (wt/vol) N,N′-methylenebisacrylamide in PBS with 0.2% (vol/vol) TEMED, and 0.2% (wt/vol) ammonium persulfate added immediately before polymerization. After gelation, samples were digested in a buffer containing 50 mM Tris–HCl pH 8.0, 1 mM EDTA, 0.5% (vol/vol) Triton X-100, 0.5 M NaCl and 8 U ml^−1^ proteinase K to enable isotropic expansion, supplemented with Hoechst. Expanded samples were transferred to diH_2_O for full expansion (~4× to 5×) overnight before imaging. After full expansion, gels were trimmed as needed and placed on glass coverslips precoated with poly-D-lysine for 30 min. During imaging, gels were kept fully submerged in diH_2_O to maintain maximal expansion and prevent distortion. Bulging structures were visualized using an upright confocal microscope with an immersive ×20 objective, and images were processed using IMARIS software.

### MACS isolation (microglia, OLs) at P15–P16

Pups were anesthetized and perfused intracardially with ice-cold 1× HBSS. Tissue was processed according to MACS Neural Tissue Dissociation Kit (P) (Miltenyi Biotec, 130-092-628). Brains were quickly removed from the skull and the cortex dissected on ice. Tissue was grossly cut into smaller pieces and added to Miltenyi gentleMACS C Tubes. Samples were placed in Miltenyi gentleMACS Dissociator (Miltenyi Biotec, 130-093-235) and the mBrain_01_02_03 protocols were run according to the manufacturer’s instructions. Once the program finished, the samples were briefly spun according to the Miltenyi protocol before being filtered through a 70-μm filter. Samples were washed with HBSS and spun to pellet cells. Myelin removal was performed using Percoll (Cytiva, 17544502) gradient as described in ref. ^69^. Cell pellets were resuspended, supplemented with anti-CD11b beads (Miltenyi Biotec, 130-097-142) for microglia isolation or anti-O4 beads (Miltenyi Biotec, 130-096-670) for OLs isolation according to Miltenyi’s protocol. Samples were resuspended in 1 ml and passed through an LS column (Miltenyi Biotec, 130-042-401) attached to a MACS Separator (Miltenyi Biotec, 130-091-051). After washing, the column was removed from the magnetic rack and magnetically labeled cells were eluted in 5 ml of buffer. Samples were centrifuged at 4,000*g* for 10 min at 4 °C, washed with 1 ml ice-cold 1× PBS and resuspend in 50 μl of RIPA buffer +PI. Samples were stored at −80 °C.

### OPC isolation for coculture

P3–P4 pups were quickly decapitated. Tissue was processed according to the MACS Neural Tissue Dissociation Kit (P) (Miltenyi Biotec, 130-092-628). Brains were quickly removed from the skull and the cortex dissected on ice. Tissue was grossly cut into smaller pieces and added to Miltenyi gentleMACS C Tubes and processed on MACS Dissociator following the manufacturer’s protocol. Samples were filtered through 70 μm and 40 μm cell strainers successively and washed with DMEM 1% FBS. Cell pellets were resuspended, supplemented with appropriate volume of FcR Blocking Reagent (10 μl per brain) for 10 min at 4 °C followed by 15 min incubation with 15 μl PDGFRα beads (CD140 (PDGFRα) beads; Miltenyi Biotec, 130-101-50) for OPC isolation. After centrifugation, cells were resuspended in 1 ml, and OPCs were collected through the LS column selection.

For OPC-microglia proliferation culture, OPCs were seeded on top of microglia, preseeded onto poly-D-lysine-coated coverslips, in a ratio of 1:1 in 96-well plates. OPCs and microglia were maintained for 4 days in a 1:1 mix of OPC proliferation medium and microglia-conditioned medium. Proliferation media consisted of DMEM (Gibco, 61965-026), N2 (Gibco, 17502048) 1×, PDGFAA (Sigma-Aldrich, P3076) 10 ng ml^−1^, bFGF (Gibco, 100-18B) 10 ng ml^−1^, penicillin–streptomycin 1%, biotin (Sigma-Aldrich, S-B4639) 10 ng ml^−1^, Hydrocortisone (Sigma-Aldrich, H0888) 20 nM, BSA 1%, FBS 0.5% and NeuroBrew21 (Miltenyi Biotec, 130-093-566) 1×. OPCs were maintained for 4 days, and the media was half changed after 2 days.

For the OPC differentiation assay, OPCs were seeded at a density of 10,000 on a 15 µl drop on poly-D-lysine-precoated coverslips in 96-well plates. After 30 min, 100 µl of media composed of a mix of 1:1 ratio of OPC differentiation medium and microglia conditioned media was added on top. Differentiation medium consisted of DMEM (Gibco, 61965-026), N2 (Gibco, 17502048) 1×, T3 (Sigma-Aldrich, S-T6397) 400 ng ml^−1^, T4 (Sigma-Aldrich, S-T0397) 400 ng ml^−1^, penicillin–streptomycin 1%, 50 μl biotin (Sigma-Aldrich, S-B4639) 10 ng ml^−1^, BSA 1%, FBS 0.5% and NeuroBrew21 (Miltenyi Biotec, 130-093-566) 1×. OPCs were then maintained for 4 days, and the media was half changed after 2 days.

### Bulk RNA-seq

Microglia from cortex were isolated from control (*n* = 3) and cKO (*n* = 5) female mice at P15–P16. Both cortices of the brain were dissected on ice and quickly homogenized in 1 ml cold medium A (HBSS, 0.6% glucose, 15 mM HEPES, 2% BSA; Sigma-Aldrich, G8270; Roth, 9157.1). Ten strokes with a loose pestle, followed by ten strokes with a tight pestle, were done in a 2 ml Dounce homogenizer. Seventy-micrometer cell strainer (pluriSelect, 43-10070-50) was prewet with 100 µl medium A and the homogenized solution was let pass through it, collecting the flow through in 5 ml Eppendorf tube placed on ice. Percoll gradient was prepared in 15 ml plastic FACS tubes (from first to last solution—2.4 ml cold HBSS, 4 ml cold Percoll 22.5% in HBSS 1X + 2% BSA; Sigma-Aldrich, GE17-5445-02). Three milliliters of 90% Percoll in 10× HBSS with phenol red (Thermo Fisher Scientific, 14170138) + 2 BSA% were mixed with the homogenized solution and the total 4 ml were placed at the bottom of the Percoll gradient. Tubes were centrifuged at 800*g* for 25 min at 4 °C without brakes. At the end of the centrifugation, myelin disk was removed, and the ring of enriched microglial cells was collected in 15-ml tubes and washed with 13 ml of 2% BSA in 1× PBS (Thermo Fisher Scientific, 10010-056). Samples were further centrifuged at 500*g* for 10 min at 4 °C and the pellet was resuspended in 200 µl of MACS buffer. Microglial cells were fluorescently sorted by means of FACS, gating for tdTomato^+^/DAPI^−^/RedDot1 Far-Red Nuclear Stain^+^ (Anawa, 40060), directly in RLT lysis buffer from RNeasy Micro Kit (Quiagen, 74004) supplemented with β-mercaptoethanol 1:100 (Sigma-Aldrich, M3148). RNA extraction was performed following the manufacturer’s instructions. RNA DNBSEQ Eukaryotic Transcriptome was later sequenced by BGI Genomics.

#### Bulk RNA-seq analysis

##### Differential gene expression

Bulk RNA-seq reads were mapped to the mouse genome (GRCm38; version M12 (Ensembl 87)) with STAR (v2.7.3a)^70^. Reads were assigned to genes using FeatureCounts SUBREAD (v2.0.0) with the following parameters: -t exon -g gene_id -s 0 -p. For the differential expression analysis, we used DESeq2 (v1.32.0)^64^ with default parameters. We filtered genes that had <5 counts in at least three samples. The gene set enrichment analysis was performed using the CERNO algorithm from the R tmod package (v0.50.07), as described in ref. ^71^.

##### DEU and CEs identification

For more sensitive splice junction discovery, we ran STAR^70^ in the two-pass mapping mode with --sjdbFileChrStartEnd, using the SJ.out.tab files generated in the one-pass mapping mode. The de novo transcript assembly for each sample was performed with StringTie (v2.2.1)^72^. StringTie –merge function was used to generate a unified nonredundant set of transcripts. For genes with undefined strandness, ‘+’ was imputed. DEU analysis was performed using the R DEXSeq package (v1.48.0)^51^. The ‘flattened’ annotation file was prepared with dexseq_prepare_annotation.py script. The exon counts were generated with dexseq_count.py with the following parameters -f bam -r pos -s no -p yes. The DEXSeqDataSet was constructed using the ‘DEXSeqDataSetFromHTSeq’ function. The experimental design was specified to model the effect of both sample and condition on exon usage. Size factors were estimated to normalize the data, and dispersions were calculated to model the biological variability across samples. DEU was assessed using the ‘testForDEU’ function, considering the specified experimental design. The DEU results were extracted using the ‘DEXSeqResults’ function. Alternative splicing events were additionally analyzed using MAJIQ (v3.0.7.dev1 + g7ff3f711). The splice junctions were extracted with ‘magic-v3 sj’. The splice graph was built with ‘majiq-v3 build’. For each RNA-seq sample, PSI values were estimated using MAJIQ’s PSI-coverage module. Differential splicing, splicing variability and statistical measures between KO and WT groups were assessed with ‘majiq-v3 deltapsi’ and ‘majiq-v3 heterogen’ modules.

### PCR-based detection of CEs

RNA from acute microglia isolation was extracted using the RNAeasy Micro Kit (Quiagen, 74004) and TRIzol (Thermo Fisher Scientific, AM9738) and retrotranscribed using the iScript cDNA synthesis kit (Bio-Rad, 1708891). PCR reactions were performed using a thermocycler (Bio-Rad, T100 Thermal Cycler) using Accustart II GelTrack PCR SuperMix (Quantabio, 84228) with the following protocol: initial denaturation—95 °C for 3 min; 40 cycles—95 °C for 15 s; 60 °C for 15 s; 72 °C for 30 s; final extension—72 °C for 5 min; hold—4 °C. PCR primers were designed to contain CE targets. Primers used for TYROBP and ATRAID were as follows: *Tyrobp* forward—CTGATTGCCCTGGCTGTGTA; *Tyrobp* reverse—TAAGGCGACTCAGTCTCAGC; *Atraid* forward—GGCACCATCTTGGGGCTAGA; *Atraid* reverse—AACCATCAGCACAAACGCAC.

### RT–qPCR

RT–qPCR was performed with the SYBR Green dye-emitted fluorescence using the Sensifast Sybr Green lo-Rox kit (Bioline, BIO-94020). Analysis was performed in triplicate in 384-well plates (1 ng cDNA per well) and run on the ViiA 7 Real-Time PCR System (Thermo Fisher Scientific). Primers were designed to amplify CE junctions for Tyrobp. Quantification of gene expression was based on the ΔCt method, normalizing each gene of interest on the geometric mean of PPIA and RPLP0 housekeeping genes. The primer sequences were as follows: *Tyrobp*_Cryptic forward—GGGTGACTTGGTGTTGACTCT; *Tyrobp*_Cryptic reverse—TCCCAGTGGCTGGATCACT; *Ppia* forward—CAAATGCTGGACCAAACACAA; *Ppia* reverse—GTTCATGCCTTCTTTCACCTTC; *Rplp0* forward—TCGTTGGAGTGACATCGTCTT; *Rplp0* reverse—GATCTGCTGCATCTGCTTGG.

### Stereotaxic injection of PSVue

Mice were anesthetized with isoflurane inhalation (induction = 0.8–1.5 l min^−1^, 4–5%; maintenance = 0.8–1.5 l min^−1^, 2–3%) and placed over a heating pad to maintain the body temperature at 37 °C. Buprenorphine (TEMGESIC, 0.1 mg kg^−1^) was injected subcutaneously in the back. Pinch reflexes were continuously monitored during the entire experiment. Local anesthetic with a mix of lidocaine (6 mg kg^−1^) and bupivacaine (2.5 mg kg^−1^) was injected subcutaneously along the line of incision. An electric drill was used to drill holes at the following coordinates: AP = +0.5 mm; ML = +1.1 mm; DV = +2.2 mm, where 2 μl of PSVue solution (1 mM PSVue; Brunschwig, POL25685-1) was unilaterally injected with Hamilton syringe (Sigma-Aldrich, 20697). The skin was then sutured, and mice were warmed on a heating pad to maintain the body temperature at 37 °C. Mice were killed 3 h postinjection with sodium pentobarbital diluted in saline (150 mg kg^−1^, i.p.) and perfused with cold HBSS, followed by PFA 4%. Brains were postfixed overnight at 4 °C and then kept in PBS with 0.02% sodium azide before slicing.

### Cloning, HEK293 cell transfections for TREM2/DAP12 and pSYK assay

A DNA fragment coding for full-length mouse *Tyrobp* (residues Gly2 to Arg114) was synthesized (Genscript), codon optimized for mammalian expression and cloned into a pLVX-EF1a-IRES lentiviral vector (Addgene, puromycin resistance). A similar approach was followed to generate a vector expressing mouse *Tyrobp* containing the CE (residues Gly2 to Lys96). Both constructs were then subcloned into a similar vector backbone containing an N-terminal HA tag. A vector expressing untagged human *TREM2* was also generated using a similar approach.

#### Cell transfections

HEK293 cells were plated at 35,000 cells per well in a 96-well plate (Biocoat, Corning) and incubated overnight at 37 °C. Next day, transfections were performed using PolyJet in vitro DNA transfection reagent’s protocol (SignaGen Labs, SL100688). The PolyJet reagent to DNA ratio was at 3:1 (µl µg^−1^) and serum-free DMEM was used to dilute PolyJet transfection reagent and plasmid DNA. The plasmid DNA was used at 0.05 µg per transfection per well of a 96-well plate. In short, the required plasmid DNA was mixed in serum-free DMEM and then required amount of PolyJet reagent was added. The mixture was vortexed and incubated for 10 min at room temperature. The transfection mix was then added to the cells in serum-containing media and incubated for 48 h. pSYK was measured by AlphaLISA assay following the manufacturer’s protocol (Perkin Elmer, ALSU-PSYK-A10K). Briefly, HEK293 cells transiently cotransfected with human-*TREM2* and mouse-*Tyrobp* (with or without CE) for 48 h were used for the assay. Cells were stimulated with 50 nM TREM2 effectorless monoclonal antibody in an IgG1 format or isotype control antibody in PBS for 5 min at 37 °C as described previously^39^. Cells were then lysed with lysis buffer containing protease and phosphatase inhibitors and stored at −80 °C. AlphaLISA assay was run as recommended in the manufacturer’s protocol and data were collected from Elmer EnVision plate reader.

HEK293 cells were fixed for 10 min with 3.7% formaldehyde in PBS and rinsed twice with a PBS solution containing 0.05% saponin and 5% BSA. Cells were then permeabilized and blocked with the same solution for 1 h at room temperature. Primary antibodies were diluted (1:250) in the same solution and incubated overnight at 4 °C. Samples were rinsed twice with PBS containing 0.05% saponin and 5% BSA and incubated with secondary antibodies (1:250) for 1.5 h at room temperature in the dark. Finally, samples were rinsed twice with 1× PBS before imaging with an Opera Phenix Imager. Primary antibodies used were anti-HA (Genescript, A01244), anti-TREM2 (R&D Systems, AF1828) and anti-Tyrobp (Abcam, ab283679).

### Proteomics

Proteomics was performed by the protein analysis facility at the University of Lausanne. Briefly, BV2 microglial cells were transfected with 20 nM siRNA targeting Tardbp or a scrambled control siRNA using Lipofectamine 3000 (Thermo Fisher Scientific, L3000001) according to the manufacturer’s instructions.

Cells were seeded at 3,000 cells per well in 96-well plates and allowed to reach ~70% confluency. siRNA and Lipofectamine 3000 were diluted separately in serum-free medium, mixed gently, and incubated for 15–20 min. Complexes were then added to the cells and incubated overnight in DMEM 5% FBS. Medium was then replaced with BV2 standard culture media (DMEM low glucose, 10% FBS, penicillin–streptomycin 1%). After 48 h, cells were treated with MG132 1 µM and BafA 100 µM for 6 h. Cells were detached using trypsin 0.05%, centrifuged and dry pellet was stored at −80 °C before use. Cells were lysed in miST lysis buffer, and protein concentration extracts were quantified. Twenty micrograms were loaded on a 15% SDS–PAGE. Low molecular weight region (<15 kDa) was cut and in-gel digested. Proteomics analysis was performed with liquid chromatography–tandem mass spectrometry (LC–MS/MS) using a semitargeted method for DAP12 canonical isoform and variant. Measurement of peptides was performed by label-free quantification of the signal intensity and data were analyzed with MaxQuant. Quantitation of the peptides of both isoforms was validated manually with Skyline.

### Behavioral testing

Behavioral characterization was performed on both males and females at the age of 4 months. Mice were handled by the experimenter for 3 consecutive days before the beginning of the test battery. Mice were habituated to the room for 1 h before experiments. Between test sessions, the apparatuses were cleaned with water, then with 70% ethanol. Light intensity was set at 10–15 lux.

#### OF test and NOR

OF was performed to assess general locomotion and anxiety behavior by placing each animal in a plexiglass box (50 × 50 × 40 cm). Mice were video scored for 10 min. Total distance traveled, time and distance in the center, and average speed were automatically evaluated by video tracking software Any-maze software (v4.99z). OF also served as a habituation for the subsequent new object test.

NOR—this was performed for 24 h after the OF test. On the training day, in the same OF arena, mice were exposed to two identical objects (placed in two opposite corners), defined as familiar objects. Mice were free to explore those objects for 15 min. Twenty-four hours later, one of the two objects was replaced with a new object, and mice were let explore the objects for 15 min (test phase). All the explorations were video recorded and analyzed offline. Time spent by the mice to explore the new object over the familiar one was taken as an indication of object recognition.

##### Y maze

Performed to assess working memory through spontaneous alternations measurement. Mice were placed in the center of a three-arm plexiglass maze with 10 cm walls and were let free to explore the three arms of the maze for 5 min. The performance will be video recorded and analyzed offline. The correct number of alternations in the arms entry during the 5 min test was evaluated and scored.

##### Elevated plus maze

Mice were placed in the central cross of the maze and were let free to explore the four arms of the maze for 5 min. Tests were video recorded, and time spent in the closed and open arms was analyzed offline.

##### Dark–light box test

Mice were placed in the dark compartment and left free to explore the two compartments of the box for 10 min. Their exploration was recorded and the time spent in the light compartment was measured offline.

##### Rotarod

Mice were placed on a rotating apparatus (Ugo Basile), for three trials, during three consecutive days with an intertrial period of 5 min. Mice were placed on the rod at a speed of 4 rpm, which accelerated over the course of 300 s to 40 rpm. The latency to fall (measured as the cumulative seconds the mouse maintains its balance on the rotarod before falling off the rod over the three trial) was scored, with a maximum latency of 300 s per trial.

##### Tail suspension test

Tail suspension test was used for the evaluation of clasping behavior. Mice were held by the tail for 2 min. Hindlimb clasping behavior was manually evaluated with the following standards: 0—no limb clasping, normal escape extension; 1—one hindlimb incomplete splay or loss of mobility; 2—two hindlimbs clasping together or loss of mobility; and 3—hindlimbs clasping together and loss of mobility.

##### Ledge test

Mice were placed onto the long ledge of the cage and their performance while walking along it was recorded. Offline scoring of the hindlimb engagement during forward motion and hind paw placement with respect to top or side of ledge was manually evaluated with the following criteria: 0—the mouse easily navigates the straight ledges and corners; 0.5—wrong paw placement on the edge of the cage but the mouse is able to walk with hindlimbs on top of the ledge for some portion of the traverse; 1—the hindlimbs repeatedly slip off the ledge but the mouse is able to walk with hindlimbs on top of the ledge for some portion of the traverse; and 2—the mouse can move across the ledge, but it does not effectively use its hindlimbs as they are consistently hugging the sides of the ledge, rather than walking on top. The score over three trials was calculated and displayed as cumulative score.

### Statistics and reproducibility

Data representation, statistical testing, and outliers’ identification were performed using GraphPad Prism (v9.3.1). Results are represented as mean ± s.em. Statistical tests used (two-tailed unpaired *t* test, one- or two-way ANOVA) and post hoc comparisons were used as appropriate and exact *p*-value, and number of biological samples were indicated in each figure legend. For multidimensional datasets, differential analyses were performed using the DESeq2, DEXSeq and MAJIQ. Multiple hypothesis testing corrections were applied using the Benjamini–Hochberg FDR procedure, with *P*_adj_ < 0.05 considered statistically significant. Exact *P* values and number of biological replicates, and tests used are reported in each figure legend. No statistical methods were used to predetermine sample sizes but our sample sizes are similar to those reported in previous publications^20,30^. Data distribution was assumed to be normal but this was not formally tested. All data were collected randomly and appropriately blocked. Data points identified as outliers were excluded from the analysis according to the predefined criteria (using the ROUT method, *Q* = 1%). Data collection and analysis were performed blind to the experimental conditions.

### Reporting summary

Further information on research design is available in the Nature Portfolio Reporting Summary linked to this article.

## Online content

Any methods, additional references, Nature Portfolio reporting summaries, source data, extended data, supplementary information, acknowledgements, peer review information; details of author contributions and competing interests; and statements of data and code availability are available at 10.1038/s41593-026-02348-3.

## Supplementary information

Reporting Summary

Supplementary Table 1 List of differentially expressed genes from Visium 10x spatial transcriptomics from P15 control and cKO mice.

Supplementary Table 2 List of differentially expressed genes from bulk RNA-seq of sorted microglia from P15 control and cKO mice.

Supplementary Table 3 DEXseq analysis of DEUs from the bulk RNA-seq of sorted microglia from P15 control and cKO mice.

Supplementary Table 4 MAJIQ splicing analysis from the bulk RNA-seq of sorted microglia from P15 control and cKO mice.

Supplementary Table 5 Proteomics analysis from scrambled and Tardbp knockdown BV2 cells.

Supplementary Video 1 Expansion microscopy showing a microglia contacting a bulging structure in P15 mouse cortex.

Supplementary Video 2 Control mouse performing the ledge test.

Supplementary Video 3 cKO mouse performing the ledge test.

## Source data

Source Data Fig. 1 Statistical source data.

Source Data Fig. 2 Statistical source data.

Source Data Fig. 3 Statistical source data.

Source Data Fig. 4 Statistical source data.

Source Data Fig. 5 Statistical source data.

Source Data Fig. 6 Statistical source data.

Source Data Fig. 7 Statistical source data.

Source Data Fig. 8 Statistical source data.

Source Data Extended Data Fig. 1 Statistical source data.

Source Data Extended Data Fig. 2 Statistical source data.

Source Data Extended Data Fig. 3 Statistical source data.

Source Data Extended Data Fig. 4 Statistical source data.

Source Data Extended Data Fig. 5 Statistical source data.

Source Data Extended Data Fig. 6 Statistical source data.

Source Data Extended Data Fig. 7 Statistical source data.

Source Data Extended Data Fig. 8 Statistical source data.

Source Data Fig. 1, 2, 5–7 and Extended Data Fig. 2–6 Unprocessed western blots and gels.

## Data Availability

10x Visium spatial transcriptomics dataset is publicly available on GEO repository under accession GSE309857. Bulk RNA-seq dataset is publicly available on GEO repository under accession GSE309858. Proteomics dataset is available on PRIDE repository under accession PXD070157. Source data are provided with this paper.

## Code availability

No custom software codes were used in the data analysis for this paper.
