# Supplementary material for: Microglial TDP-43 mediates myelin refinement and represses Tyrobp cryptic exon inclusion in mice
Source: Nat Neurosci. 2026 Jul 8;29(8):1841–57. doi: 10.1038/s41593-026-02348-3 (PMC13433257; doi:10.1038/s41593-026-02348-3)
Supplement: Supplementary file 1 [file 41593_2026_2348_MOESM1_ESM.pdf]

nature portfolio

Corresponding author(s): Rosa Chiara Paolicelli

Last updated by author(s): May 3, 2026

Reporting Summary

Nature Portfolio wishes to improve the reproducibility of the work that we publish. This form provides structure for consistency and transparency in reporting. For further information on Nature Portfolio policies, see our Editorial Policies and the Editorial Policy Checklist.

Statistics

For all statistical analyses, confirm that the following items are present in the figure legend, table legend, main text, or Methods section.

n/a	Confirmed	
☐	☒	The exact sample size (*n*) for each experimental group/condition, given as a discrete number and unit of measurement
☐	☒	A statement on whether measurements were taken from distinct samples or whether the same sample was measured repeatedly
☐	☒	The statistical test(s) used AND whether they are one- or two-sided<br>*Only common tests should be described solely by name; describe more complex techniques in the Methods section.*
☐	☒	A description of all covariates tested
☐	☒	A description of any assumptions or corrections, such as tests of normality and adjustment for multiple comparisons
☐	☒	A full description of the statistical parameters including central tendency (e.g. means) or other basic estimates (e.g. regression coefficient) AND variation (e.g. standard deviation) or associated estimates of uncertainty (e.g. confidence intervals)
☐	☒	For null hypothesis testing, the test statistic (e.g. *F*, *t*, *r*) with confidence intervals, effect sizes, degrees of freedom and *P* value noted<br>*Give P values as exact values whenever suitable.*
☒	☐	For Bayesian analysis, information on the choice of priors and Markov chain Monte Carlo settings
☒	☐	For hierarchical and complex designs, identification of the appropriate level for tests and full reporting of outcomes
☒	☐	Estimates of effect sizes (e.g. Cohen's *d*, Pearson's *r*), indicating how they were calculated

Our web collection on statistics for biologists contains articles on many of the points above.

Software and code

Policy information about availability of computer code

Data collection

Image acquisition: Leica Stellaris 8 running LAS X software
Electron microscopy: Zeiss Crossbeam 350 SEM running ATLAS 5 software
Flow cytometry: Beckman Coulter CytoFLEX S running CytExpert software
FACS: BD Discover S8 1
Sequencing (Bulk RNAseq, Spatial transcriptomics): BGI DNBSEQ™
RT-qPCR: ThermoFisher Scientific ViiA 7 Real-Time PCR System
Western-Blot: Li-Cor Odyssey XF
Proteomic: Orbitrap Exploris 480 mass spectrometer (Thermo Fisher)
Behavior: Any-maze software (version 4.99z)

Data analysis

Flow cytometry: FlowJo (version 10.10.0)
Image analysis: Fiji/ImageJ (version 1.54) , IMARIS bitplane (version 10.2.0)
Spatial transcriptomic: R studio, spaceranger version 1.3.1, R Seurat package version 4.1.1; DESeq2 version-1.32.0
Bulk RNAseq: R studio, STAR version-2.7.3a, DESeq2 version-1.32.0, R tmod package version-0.50.07, StringTie Version 2.2.1, DEXSeq package, version 1.48.0, MAJIQ (3.0.7.dev1+g7ff3f711)
Western-Blot: Image Studio Lite Ver 5.2
Proteomic: MaxQuant 2.6.7.0
Behavioral analysis: Any-maze software (version 4.99z)
GraphPad Prism v10 was used for graph design and statistical analysis.

For manuscripts utilizing custom algorithms or software that are central to the research but not yet described in published literature, software must be made available to editors and reviewers. We strongly encourage code deposition in a community repository (e.g. GitHub). See the Nature Portfolio guidelines for submitting code & software for further information.

Data

Policy information about availability of data

All manuscripts must include a data availability statement. This statement should provide the following information, where applicable:

- Accession codes, unique identifiers, or web links for publicly available datasets
- A description of any restrictions on data availability
- For clinical datasets or third party data, please ensure that the statement adheres to our policy

10x Visium Spatial transcriptomics dataset is publicly available on GEO repository, accession number: GSE309857. BulkRNA-sequencing dataset is publicly available on GEO repository, accession number: GSE309858. Proteomics dataset is available on PRIDE repository, accession number: PXD070157. Source Data are provided with this manuscript.

Research involving human participants, their data, or biological material

Policy information about studies with human participants or human data. See also policy information about sex, gender (identity/presentation), and sexual orientation and race, ethnicity and racism.

Reporting on sex and gender	*Use the terms sex (biological attribute) and gender (shaped by social and cultural circumstances) carefully in order to avoid confusing both terms. Indicate if findings apply to only one sex or gender; describe whether sex and gender were considered in study design; whether sex and/or gender was determined based on self-reporting or assigned and methods used. Provide in the source data disaggregated sex and gender data, where this information has been collected, and if consent has been obtained for sharing of individual-level data; provide overall numbers in this Reporting Summary. Please state if this information has not been collected. Report sex- and gender-based analyses where performed, justify reasons for lack of sex- and gender-based analysis.*
Reporting on race, ethnicity, or other socially relevant groupings	*Please specify the socially constructed or socially relevant categorization variable(s) used in your manuscript and explain why they were used. Please note that such variables should not be used as proxies for other socially constructed/relevant variables (for example, race or ethnicity should not be used as a proxy for socioeconomic status). Provide clear definitions of the relevant terms used, how they were provided (by the participants/respondents, the researchers, or third parties), and the method(s) used to classify people into the different categories (e.g. self-report, census or administrative data, social media data, etc.) Please provide details about how you controlled for confounding variables in your analyses.*
Population characteristics	*Describe the covariate-relevant population characteristics of the human research participants (e.g. age, genotypic information, past and current diagnosis and treatment categories). If you filled out the behavioural & social sciences study design questions and have nothing to add here, write "See above."*
Recruitment	*Describe how participants were recruited. Outline any potential self-selection bias or other biases that may be present and how these are likely to impact results.*
Ethics oversight	*Identify the organization(s) that approved the study protocol.*

Note that full information on the approval of the study protocol must also be provided in the manuscript.

Field-specific reporting

Please select the one below that is the best fit for your research. If you are not sure, read the appropriate sections before making your selection.

☒ Life sciences ☐ Behavioural & social sciences ☐ Ecological, evolutionary & environmental sciences

For a reference copy of the document with all sections, see nature.com/documents/nr-reporting-summary-flat.pdf

Life sciences study design

All studies must disclose on these points even when the disclosure is negative.

Sample size	No statistical methods were used to predetermine sample sizes. Samples sizes were chosen to allow statistical testing and varied depending on the breeding capacity of the mouse lines used (litter sizes).
Data exclusions	All datasets were tested for statistical outliers, with predefined exclusion criteria established prior to data collection (ROUT method, Q=1%). Exclusions were applied only where data quality was unequivocally compromised by technical issues (e.g., imaging artifacts or poor sample integrity). For behavioral experiments, animals were excluded solely when pre-existing conditions impaired valid task performance, defined a priori at the analysis stage as minimal locomotor activity or insufficient object exploration in the novel object recognition (NOR) test. All exclusions were applied consistently across groups.
Replication	To confirm the reproducibility of the experimental results, each experiment was independently replicated at least two times.

Randomization	Mice were randomly allocated to each experimental group for all experiments.
Blinding	All quantification experiments were performed in a blinded manner (experimenters unaware of genotype) by assignment of unidentifiable numbers to acquired images or behavioral videos.

Behavioural & social sciences study design

All studies must disclose on these points even when the disclosure is negative.

Study description	*Briefly describe the study type including whether data are quantitative, qualitative, or mixed-methods (e.g. qualitative cross-sectional, quantitative experimental, mixed-methods case study).*
Research sample	*State the research sample (e.g. Harvard university undergraduates, villagers in rural India) and provide relevant demographic information (e.g. age, sex) and indicate whether the sample is representative. Provide a rationale for the study sample chosen. For studies involving existing datasets, please describe the dataset and source.*
Sampling strategy	*Describe the sampling procedure (e.g. random, snowball, stratified, convenience). Describe the statistical methods that were used to predetermine sample size OR if no sample-size calculation was performed, describe how sample sizes were chosen and provide a rationale for why these sample sizes are sufficient. For qualitative data, please indicate whether data saturation was considered, and what criteria were used to decide that no further sampling was needed.*
Data collection	*Provide details about the data collection procedure, including the instruments or devices used to record the data (e.g. pen and paper, computer, eye tracker, video or audio equipment) whether anyone was present besides the participant(s) and the researcher, and whether the researcher was blind to experimental condition and/or the study hypothesis during data collection.*
Timing	*Indicate the start and stop dates of data collection. If there is a gap between collection periods, state the dates for each sample cohort.*
Data exclusions	*If no data were excluded from the analyses, state so OR if data were excluded, provide the exact number of exclusions and the rationale behind them, indicating whether exclusion criteria were pre-established.*
Non-participation	*State how many participants dropped out/declined participation and the reason(s) given OR provide response rate OR state that no participants dropped out/declined participation.*
Randomization	*If participants were not allocated into experimental groups, state so OR describe how participants were allocated to groups, and if allocation was not random, describe how covariates were controlled.*

Ecological, evolutionary & environmental sciences study design

All studies must disclose on these points even when the disclosure is negative.

Study description	*Briefly describe the study. For quantitative data include treatment factors and interactions, design structure (e.g. factorial, nested, hierarchical), nature and number of experimental units and replicates.*
Research sample	*Describe the research sample (e.g. a group of tagged Passer domesticus, all Stenocereus thurberi within Organ Pipe Cactus National Monument), and provide a rationale for the sample choice. When relevant, describe the organism taxa, source, sex, age range and any manipulations. State what population the sample is meant to represent when applicable. For studies involving existing datasets, describe the data and its source.*
Sampling strategy	*Note the sampling procedure. Describe the statistical methods that were used to predetermine sample size OR if no sample-size calculation was performed, describe how sample sizes were chosen and provide a rationale for why these sample sizes are sufficient.*
Data collection	*Describe the data collection procedure, including who recorded the data and how.*
Timing and spatial scale	*Indicate the start and stop dates of data collection, noting the frequency and periodicity of sampling and providing a rationale for these choices. If there is a gap between collection periods, state the dates for each sample cohort. Specify the spatial scale from which the data are taken*
Data exclusions	*If no data were excluded from the analyses, state so OR if data were excluded, describe the exclusions and the rationale behind them, indicating whether exclusion criteria were pre-established.*
Reproducibility	*Describe the measures taken to verify the reproducibility of experimental findings. For each experiment, note whether any attempts to repeat the experiment failed OR state that all attempts to repeat the experiment were successful.*
Randomization	*Describe how samples/organisms/participants were allocated into groups. If allocation was not random, describe how covariates were controlled. If this is not relevant to your study, explain why.*
Blinding	*Describe the extent of blinding used during data acquisition and analysis. If blinding was not possible, describe why OR explain why*

Blinding | *blinding was not relevant to your study.*

Did the study involve field work? ☐ Yes ☐ No

Field work, collection and transport

Field conditions | *Describe the study conditions for field work, providing relevant parameters (e.g. temperature, rainfall).*

Location | *State the location of the sampling or experiment, providing relevant parameters (e.g. latitude and longitude, elevation, water depth).*

Access & import/export | *Describe the efforts you have made to access habitats and to collect and import/export your samples in a responsible manner and in compliance with local, national and international laws, noting any permits that were obtained (give the name of the issuing authority, the date of issue, and any identifying information).*

Disturbance | *Describe any disturbance caused by the study and how it was minimized.*

Reporting for specific materials, systems and methods

We require information from authors about some types of materials, experimental systems and methods used in many studies. Here, indicate whether each material, system or method listed is relevant to your study. If you are not sure if a list item applies to your research, read the appropriate section before selecting a response.

Materials & experimental systems

n/a	Involved in the study
☐	☒ Antibodies
☐	☒ Eukaryotic cell lines
☒	☐ Palaeontology and archaeology
☐	☒ Animals and other organisms
☒	☐ Clinical data
☒	☐ Dual use research of concern
☒	☐ Plants

Methods

n/a	Involved in the study
☒	☐ ChIP-seq
☐	☒ Flow cytometry
☐	☒ MRI-based neuroimaging

Antibodies

Antibodies used

Immunofluorescence primary antibody: MBP (Proteintech, #10458-1-AP, 1:400); OLIG2 (Merck Millipore, #AB9610, 1:400); CC1/APC (Anti-APC (Ab-7) Mouse mAb (CC-1), Merck Millipore, #OP80, 1:100); PDGFRa (CD140a, Merck Millipore, #CBL1366, 1:400); CD68 (Bio-Rad, #MCA1957, 1:400); TDP-43 (Proteintech, #10782-2-AP, 1:100); NF200 (Bio-Techne Sales Corp.; cat#NB300-217; 1:80); KI67 (Invitrogen; cat#14-5698-82 1:300 and Abcam, cat #ab15580; 1:1000); Stat1(Cell Signaling Technology; cat#14994S, 1:400).

immunofluorescence secondary antibody: all dilution 1:1000, except when specified - Donkey anti-Mouse IgG (H+L), Alexa Fluor Plus 488 (Thermo Fisher Scientific; cat#A32766); Donkey anti-Rabbit IgG (H+L), Alexa Fluor Plus 488 (Thermo Fisher Scientific; cat#A32790); Donkey anti-Rabbit IgG (H+L), Alexa Fluor Plus 647 (Thermo Fisher Scientific; cat#A32795); Goat anti-Mouse IgG (H+L), Alexa Fluor Plus 647 (Thermo Fisher Scientific; cat#A32728); Goat anti-Rat IgG (H+L), Alexa Fluor 350 (Thermo Fisher Scientific; cat#A21093); Goat anti-Rat IgG (H+L), Alexa Fluor 647; Thermo Fisher Scientific; cat#A21247); Donkey anti-Rat IgG (H+L), Alexa Fluor 488 (Thermo Fisher Scientific; cat#A21208); Goat Anti-chicken IgY, Fluoresceinated (Aves Labs; cat#F-1005; 1:200)

Western-Blot primary antibody: OLIG2 (Merck Millipore, #AB9610, 1:3500); MBP (Proteintech, #10458-1-AP, 1:2000); TDP-43 (Proteintech, #10782-2-AP, 1:1000); IBA1(Sigma Aldrich, #ZMS1024, 1:1000); GFAP (Sigma-Aldrich, #G3893, 1:1000); SYK (Cell Signaling, #13198, 1:1000); Phospho-SYK (Cell Signaling, # 2717, 1:1000); STAT1 (Cell Signaling, #14994T, 1:1000); DAP12 (Abcam, #ab283679, 1:1000); Actin (Abcam, #ab8227, 1:5000); GAPDH (Proteintech, #60004-1-Ig, 1:5000); Ubiquitin (Sigma-Aldrich #662099, 1:1000).
Western-Blot secondary antibody: all dultions. 1:15000 - Donkey anti-Mouse IgG, IRDye 680CW (LI-COR Biosciences; cat#926-68072) ; Donkey anti-Mouse IgG, IRDye 800CW (LI-COR Biosciences; cat#926-32212); Donkey anti-Rabbit IgG, IRdye 800CW (LI-COR Biosciences; cat#926-32213)

Validation

All primary antibodies used for immunofluorescence and immunoblotting have been validated for
this application by the supplier as indicated on the websites and datasheets of the coording antibodies.
Companies, category numbers and/or clone names are indicated for all used antibodies.

Eukaryotic cell lines

Policy information about cell lines and Sex and Gender in Research

Cell line source(s)	BV-2 (RRID:CVCL_0182). The BV-2 cell line is an immortalized microglial cell line derived from 1week old female C57BL/6 mice. The cells are immortalized by v-raf/v-myc carrying J2 retrovirus.
Authentication	Not authenticated
Mycoplasma contamination	Negative
Commonly misidentified lines (See ICLAC register)	BV-2 (RRID:CVCL_0182)

Animals and other research organisms

Policy information about studies involving animals; ARRIVE guidelines recommended for reporting animal research, and Sex and Gender in Research

Laboratory animals	Cx3cr1CREERT2;Tardbpflox mice were obtained by crossing the B6.129P2(Cg)-Cx3cr1tm2.1(cre/ERT2)Litt/WganJ mice (Cx3cr1CREERT2; No: 021160, The Jackson Laboratory) with and B6(SJL)-Tardbptm1.1Pcw/J (Tardbpfloxed/floxed, No:017591, The Jackson Laboratory). The Cx3cr1CREERT2;Tardbpflox line was further crossed with B6.Cg-Gt(ROSA)26Sortm14(CAG-tdTomato)Hze/J mice (TdTomatoflox; No: 007914, The Jackson Laboratory).<br>All the experimental subjects were obtained by crossing Cx3cr1creERT2/creERT2;Tardbpfloxed/wt with TdTomatoflox/flox;Tardbpfloxed/wt, in order to produce littermates that were all heterozygous for Cx3cr1creERT2 and tdTomato, and wild-type (WT) or homozygous (cKO) for the Tardbp floxed allele.<br><br>TREM2 knockout (KO) mice were provided by Prof Michela Matteoli and DAP12 KO mice were provided by Dr Sonia Garel.
Wild animals	The study did not involve wild animals.
Reporting on sex	Most of the experiments were done using both sexes. Since no sex differences were observed some analysis were done on one sex for practical or technical reasons only listed below :<br>Electron microscopy and P12 analysis were done on male mice.<br>Bulk RNAsequencing was done on female mice.
Field-collected samples	The study did not involve samples collected from the field.
Ethics oversight	All animal experiments were authorized by the Service de la consommation et des Affaires vétérinaires (SCAV) of the Canton de Vaud in Switzerland, and by the Direction générale de l'agriculture, de la viticulture et des affaires vétérinaires (DGAV) - Affaires vétérinaires Protection des animaux, under the animal license VD3868 and VD3491. Mice were bred and maintained in the animal facility of the University of Lausanne.

Note that full information on the approval of the study protocol must also be provided in the manuscript.

Plants

Seed stocks	*Report on the source of all seed stocks or other plant material used. If applicable, state the seed stock centre and catalogue number. If plant specimens were collected from the field, describe the collection location, date and sampling procedures.*
Novel plant genotypes	*Describe the methods by which all novel plant genotypes were produced. This includes those generated by transgenic approaches, gene editing, chemical/radiation-based mutagenesis and hybridization. For transgenic lines, describe the transformation method, the number of independent lines analyzed and the generation upon which experiments were performed. For gene-edited lines, describe the editor used, the endogenous sequence targeted for editing, the targeting guide RNA sequence (if applicable) and how the editor was applied.*
Authentication	*Describe any authentication procedures for each seed stock used or novel genotype generated. Describe any experiments used to assess the effect of a mutation and, where applicable, how potential secondary effects (e.g. second site T-DNA insertions, mosiacism, off-target gene editing) were examined.*

Flow Cytometry

Plots

Confirm that:

☒ The axis labels state the marker and fluorochrome used (e.g. CD4-FITC).

☒ The axis scales are clearly visible. Include numbers along axes only for bottom left plot of group (a 'group' is an analysis of identical markers).

☒ All plots are contour plots with outliers or pseudocolor plots.

☒ A numerical value for number of cells or percentage (with statistics) is provided.

Methodology

Sample preparation	MACS isolation of CD11b+ cells from P15 control or cKO brains.<br>Detailed in Material and Methods section: "Acute cell isolation (microglia, oligodendrocytes)" and "Myelin engulfment assay by flow cytometry"
Instrument	Beckman Coulter CytoFLEX S
Software	CytExpert software to collect the data and FlowJo (version 10.10.0) to analyse the data
Cell population abundance	Microglia was sorted using CD11b+ beads with MACS (Miltenyi biotech). From one P15 brain we obtained approximately 400000 TdTomato+ cells, which correspond to 90% of gated live cells.<br>At least 15000 cells were gated for both FACS and flow cytometry analysis.
Gating strategy	For flow cytometry, FCS/SSC area gates to select cells, FSC-H/FSC-A to select single cells on cells, SSA-A/DAPI, to select negative population (live cells) on single cells, SSC-A/TdTomato+ for microglia population on live cells.<br>A similar strategy for FACS was used. We added a FACS RedDot™1 Far-Red Nuclear Stain+ on top of DAPI selection to select live cells.

☒ Tick this box to confirm that a figure exemplifying the gating strategy is provided in the Supplementary Information.

Magnetic resonance imaging

Experimental design

Design type	*Indicate task or resting state; event-related or block design.*
Design specifications	*Specify the number of blocks, trials or experimental units per session and/or subject, and specify the length of each trial or block (if trials are blocked) and interval between trials.*
Behavioral performance measures	*State number and/or type of variables recorded (e.g. correct button press, response time) and what statistics were used to establish that the subjects were performing the task as expected (e.g. mean, range, and/or standard deviation across subjects).*

Acquisition

Imaging type(s)	Diffusion Weighted Imaging
Field strength	7T
Sequence & imaging parameters	high-resolution multi-shot spin-echo echo-planar imaging sequence with 4 segments along 90 encoding directions and b-values of 0, 1000, 3000 s/mm2. Other parameters were: TR = 2000 ms, TE = 22 ms, in-plane RES = ~ 0.2 mm2, slice thickness = 0.4 mm, for a total scan time of ≈8 hours.
Area of acquisition	whole-brain ex-vivo scans

Diffusion MRI ☒ Used ☐ Not used

Preprocessing

Preprocessing software	Preprocessing and diffusion data analysis was carried out using standard pre-processing steps in FSL and Matlab scripts (denoising, Gibbs ringing correction, distorition and eddy current correction) followed by diffusion tensor estimation using a weighted linear least-squares algorithm (Veraart et, NeuroImage 2013).
Normalization	The volumes were spatially normalized to a study-specific template using ANTs (Advanced Normalization Tools, hKp://picsl.upenn.edu/sohware/ants/, RRID:SCR_004757). Statistics were performed using FSL (Analysis 1120 Group, FMRIB, Oxford, UK, RRID:SCR_002823).

Normalization template	We used an internally-generated, study-specific standard space template.
Noise and artifact removal	N/A
Volume censoring	N/A

Statistical modeling & inference

Model type and settings	*Specify type (mass univariate, multivariate, RSA, predictive, etc.) and describe essential details of the model at the first and second levels (e.g. fixed, random or mixed effects; drift or auto-correlation).*
Effect(s) tested	*Define precise effect in terms of the task or stimulus conditions instead of psychological concepts and indicate whether ANOVA or factorial designs were used.*

Specify type of analysis: ☒ Whole brain ☐ ROI-based ☐ Both

Statistic type for inference (See Eklund et al. 2016)	*Specify voxel-wise or cluster-wise and report all relevant parameters for cluster-wise methods.*
Correction	*Describe the type of correction and how it is obtained for multiple comparisons (e.g. FWE, FDR, permutation or Monte Carlo).*

Models & analysis

n/a	Involved in the study
☐	☐ Functional and/or effective connectivity
☐	☐ Graph analysis
☐	☐ Multivariate modeling or predictive analysis

Functional and/or effective connectivity	*Report the measures of dependence used and the model details (e.g. Pearson correlation, partial correlation, mutual information).*
Graph analysis	*Report the dependent variable and connectivity measure, specifying weighted graph or binarized graph, subject- or group-level, and the global and/or node summaries used (e.g. clustering coefficient, efficiency, etc.).*
Multivariate modeling and predictive analysis	*Specify independent variables, features extraction and dimension reduction, model, training and evaluation metrics.*
