# Supplementary material for: Microglial TDP-43 mediates myelin refinement and represses Tyrobp cryptic exon inclusion in mice
Source: Nat Neurosci. 2026 Jul 8;29(8):1841–57. doi: 10.1038/s41593-026-02348-3 (PMC13433257; doi:10.1038/s41593-026-02348-3)

Fig1c Blot

Total protein stain

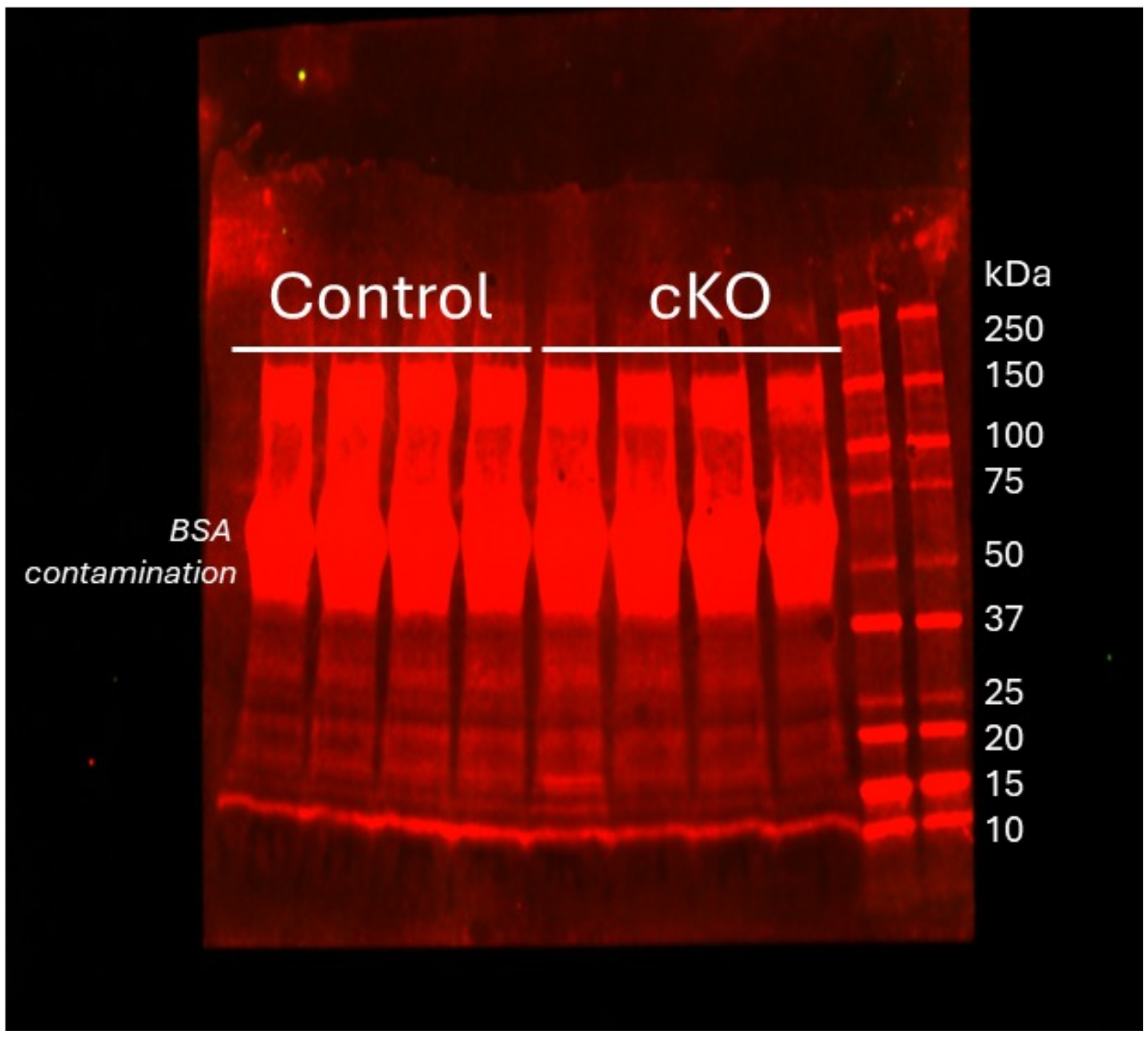

IBA1

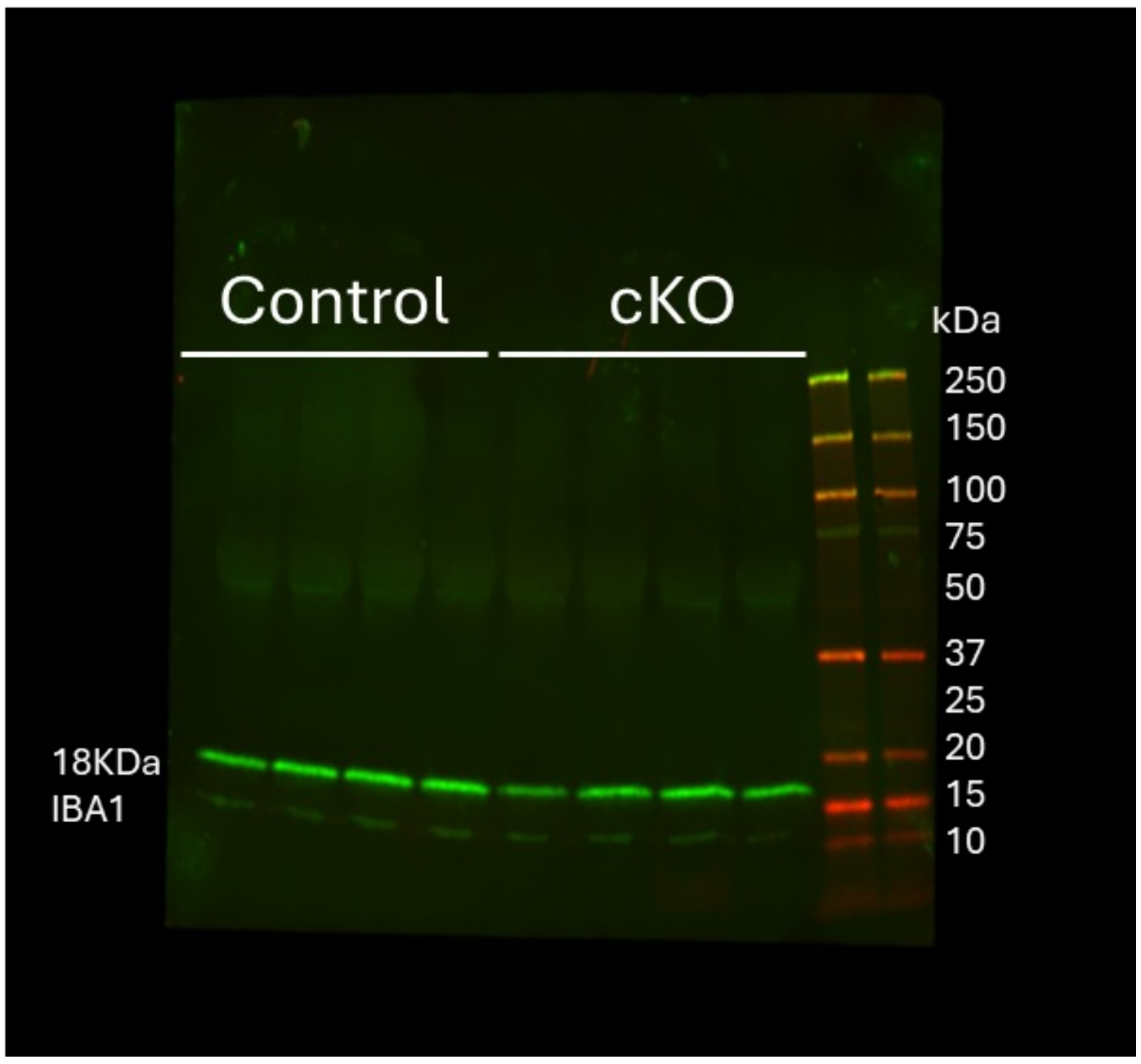

TDP-43

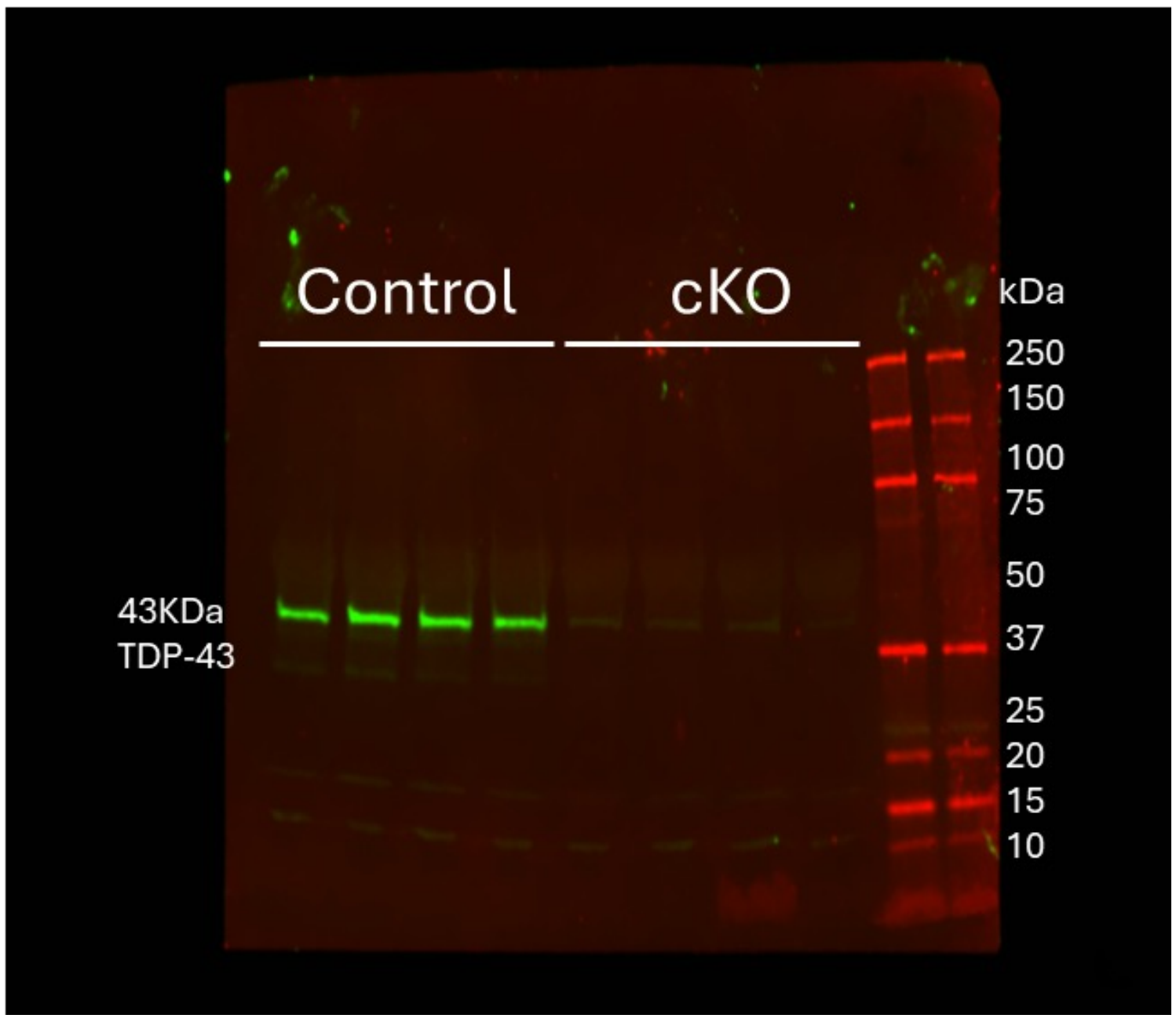

Fig2f Blot

Total protein stain

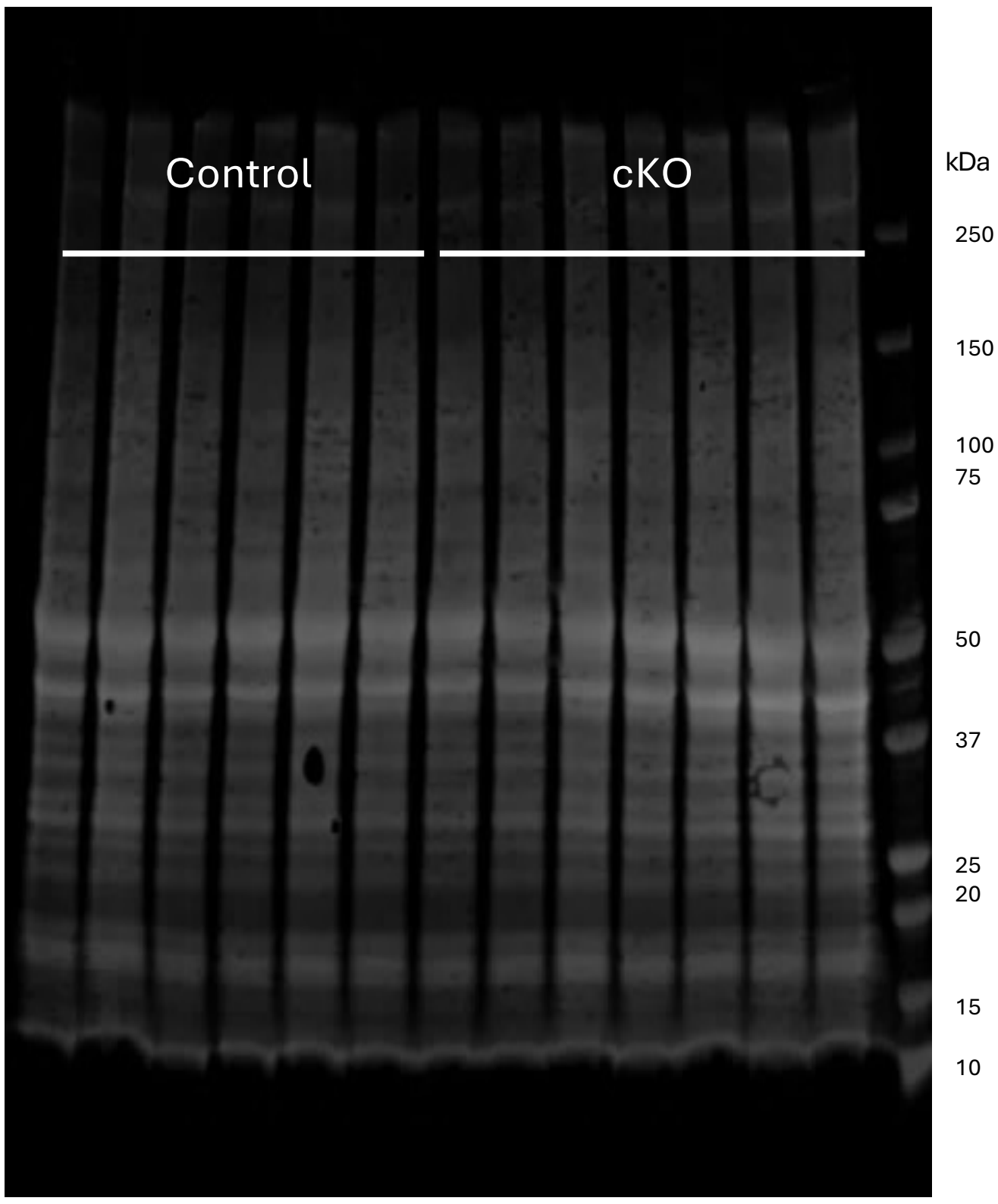

MBP

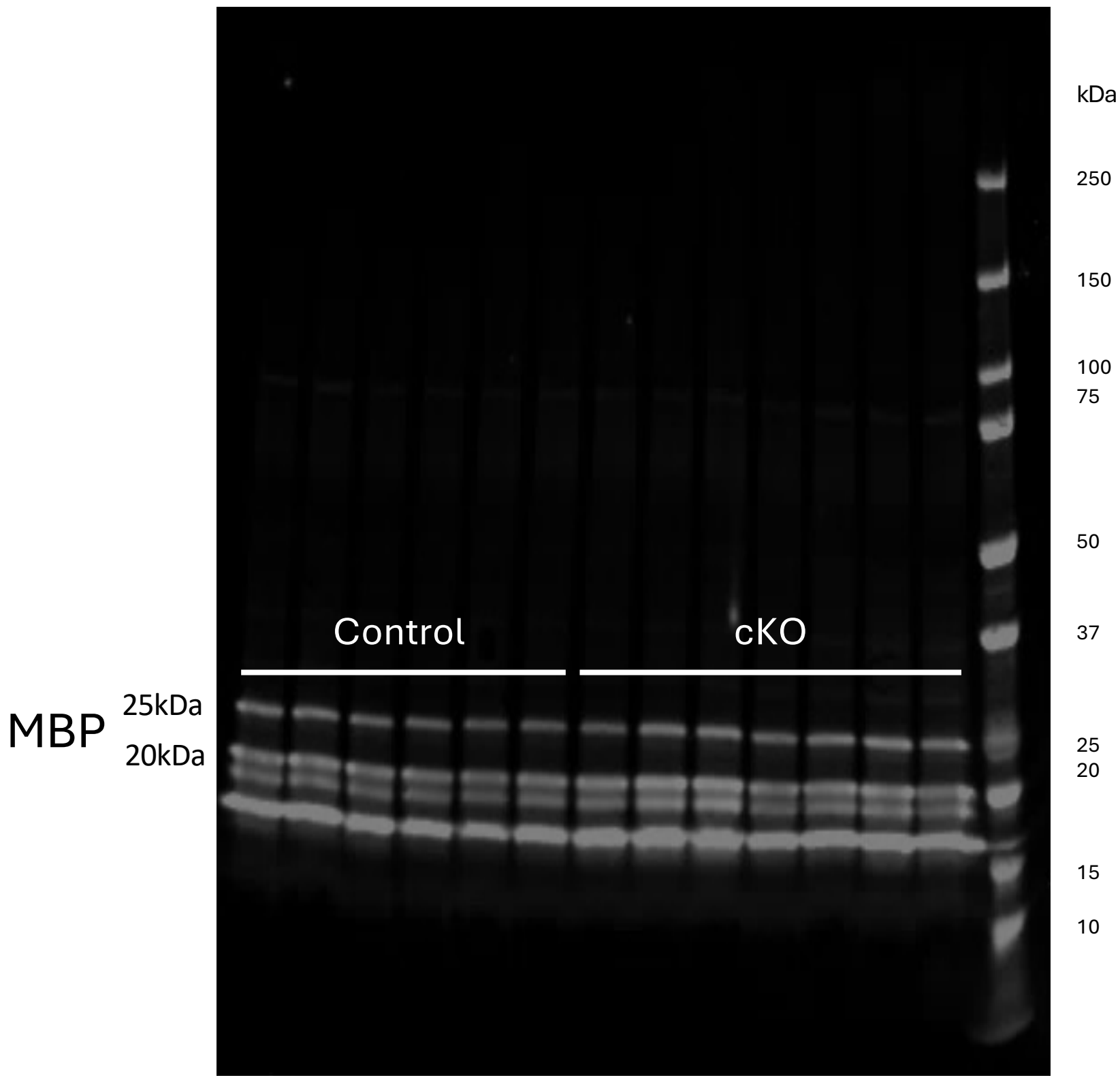

Fig5n Blot

Total protein stain

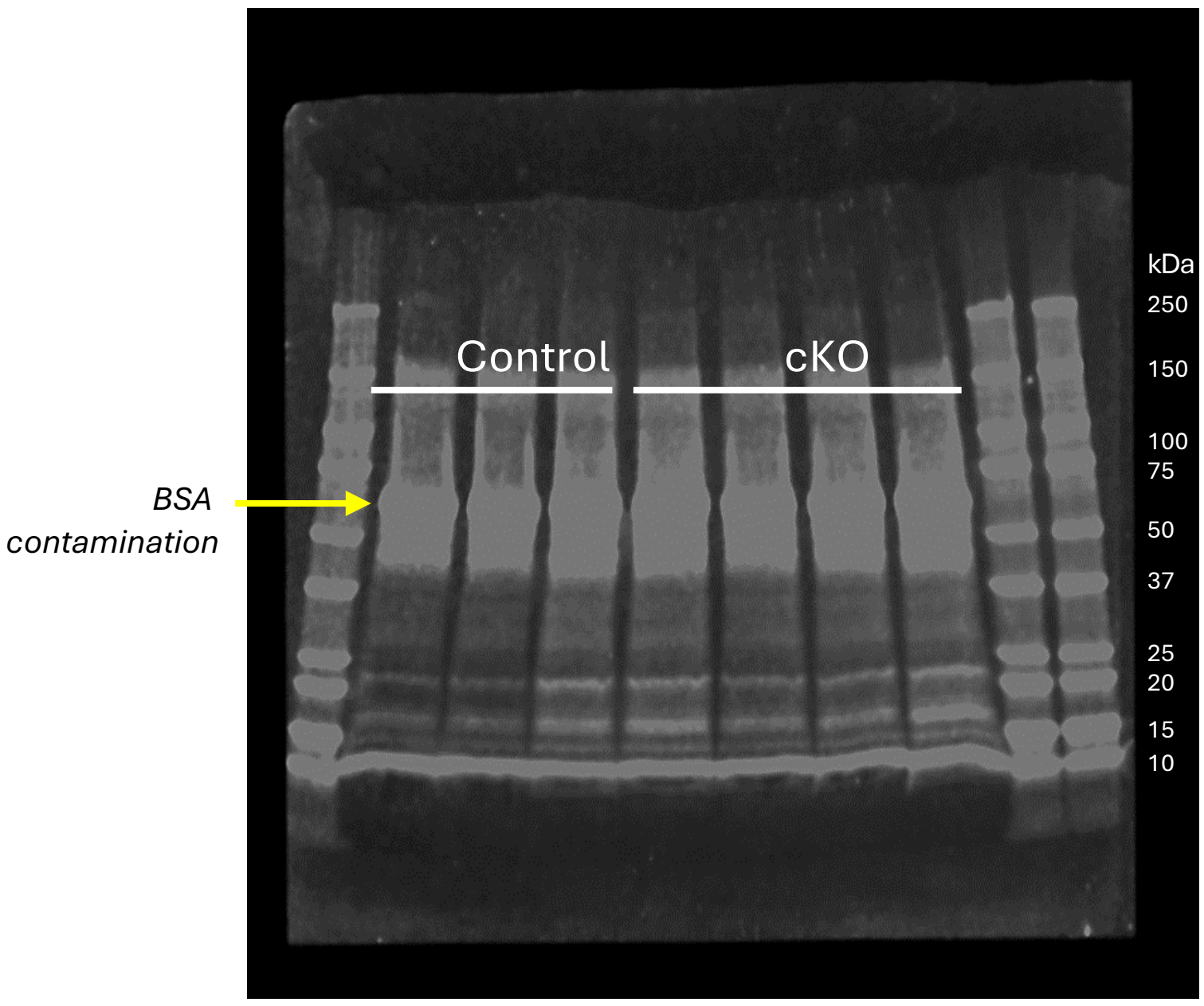

STAT1

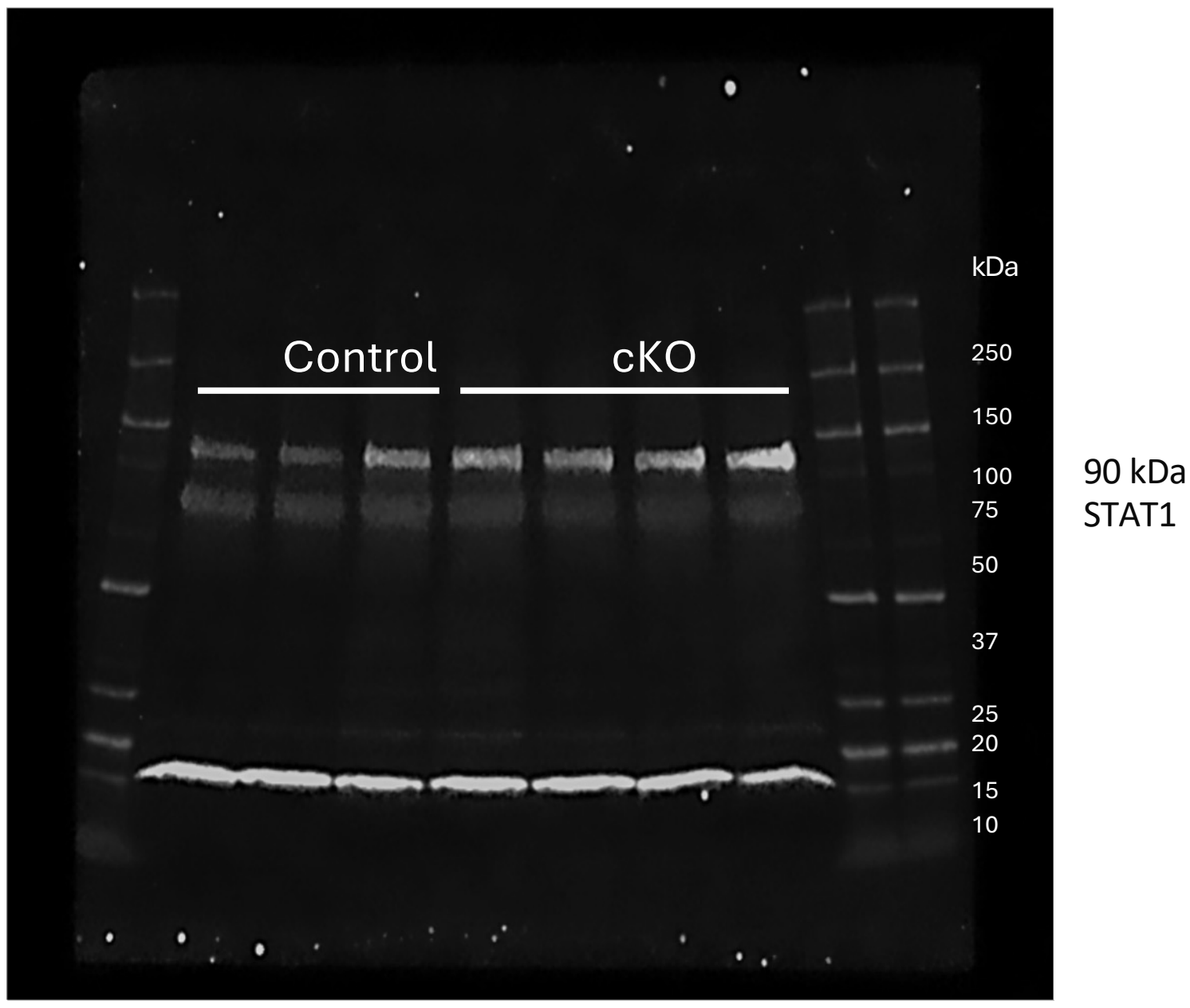

Fig6j Gel

Tyrobp RT-PCR amplification

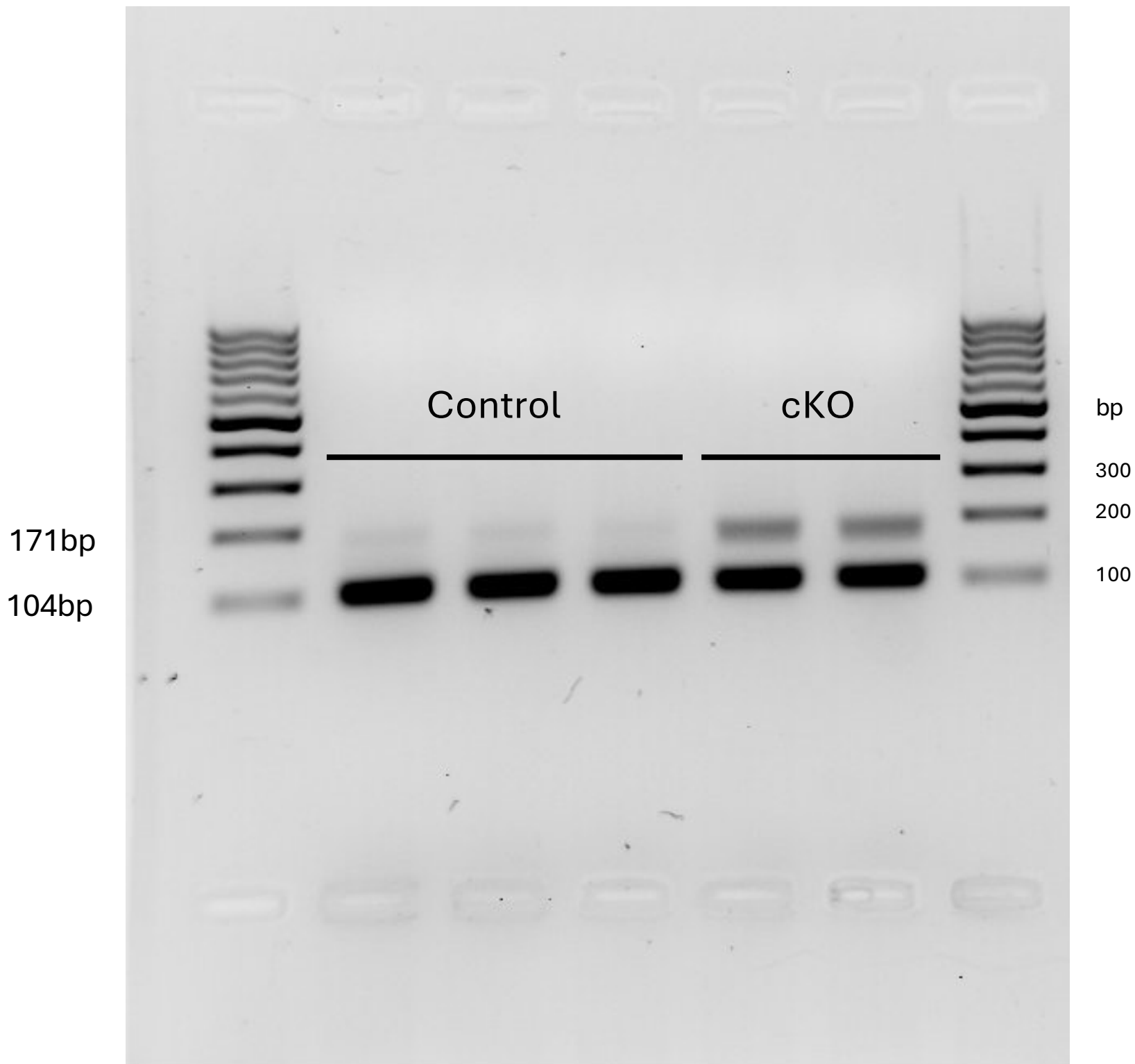

Fig6k Blot

ACTIN - GAPDH

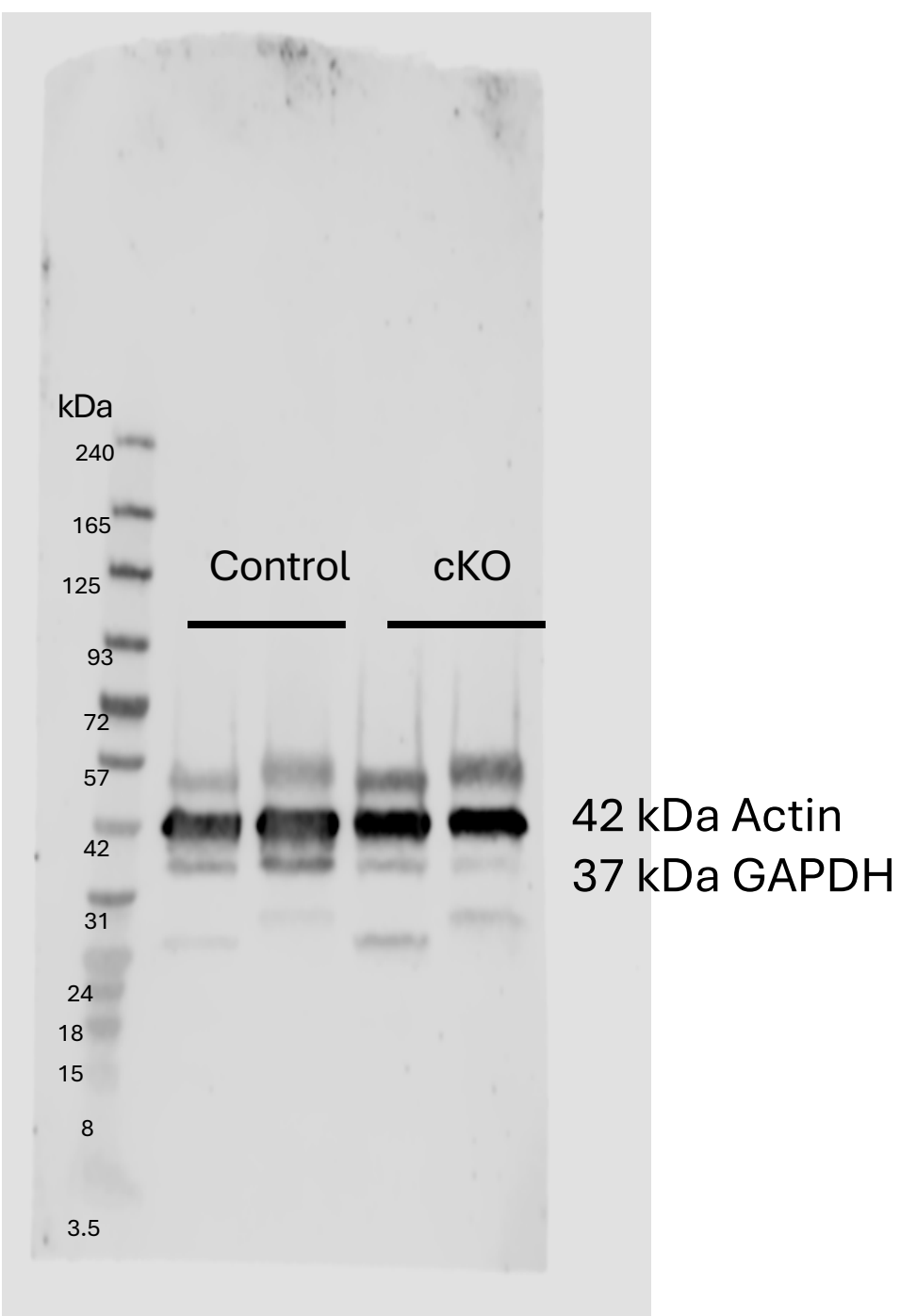

TDP-43

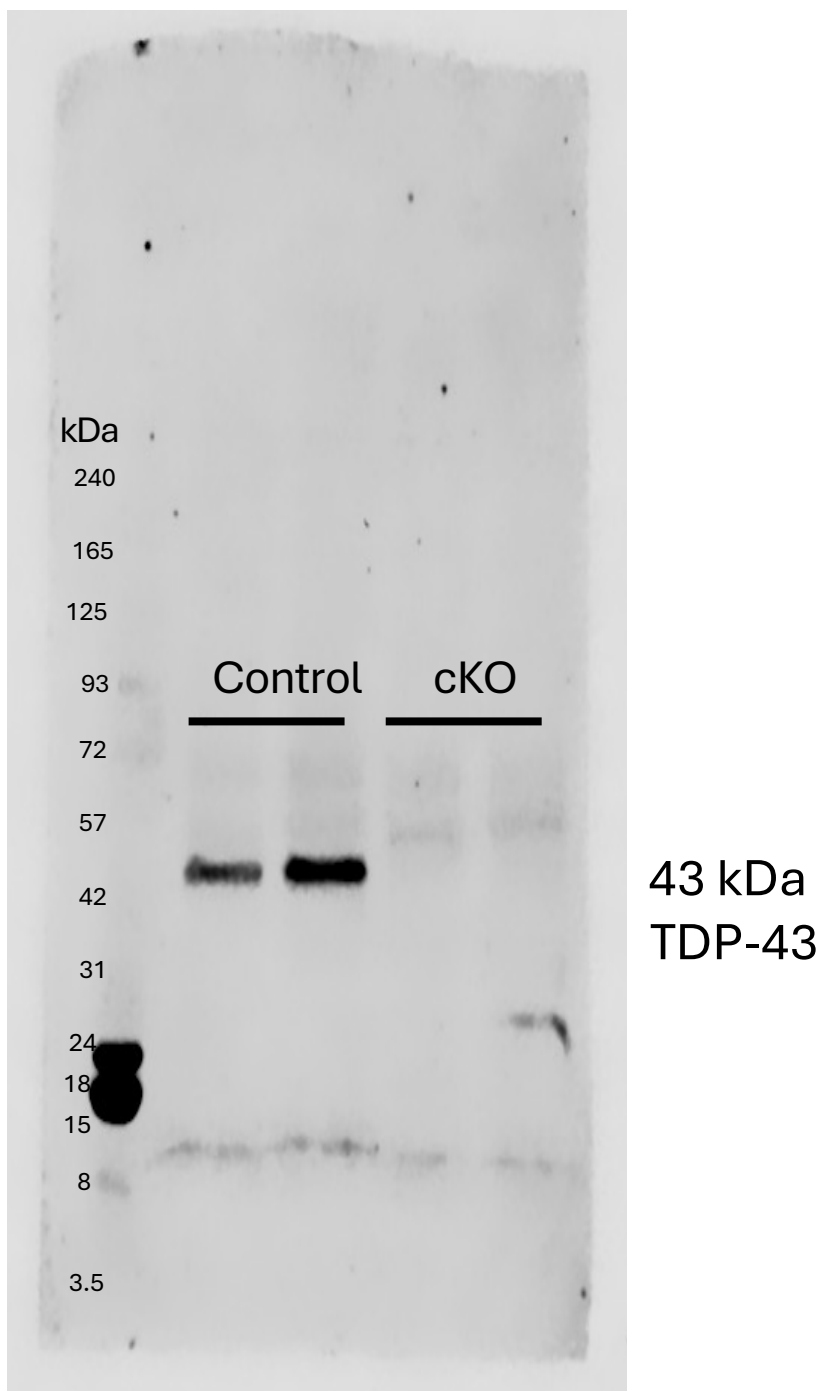

DAP-12

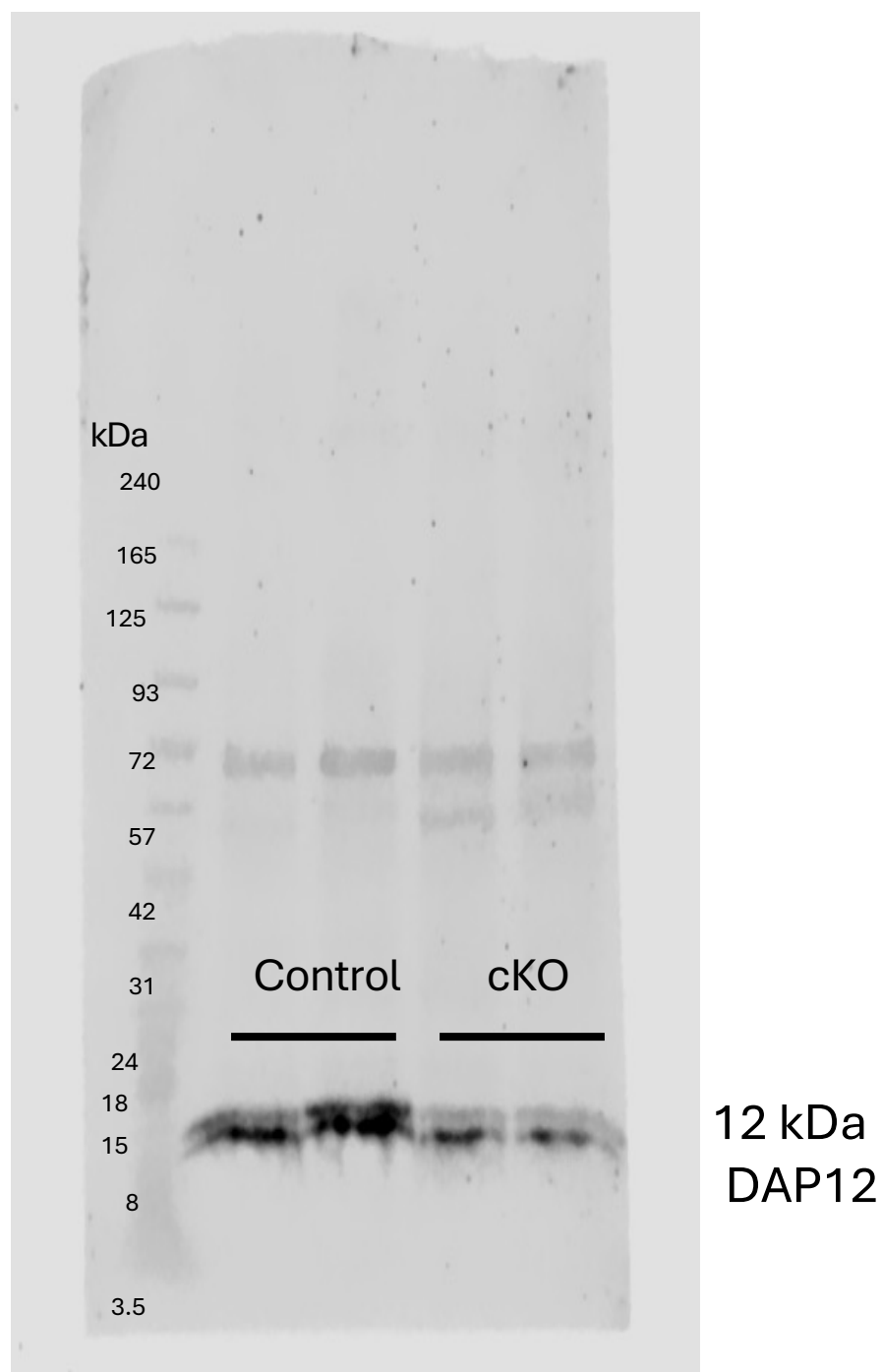

Fig7c Blot

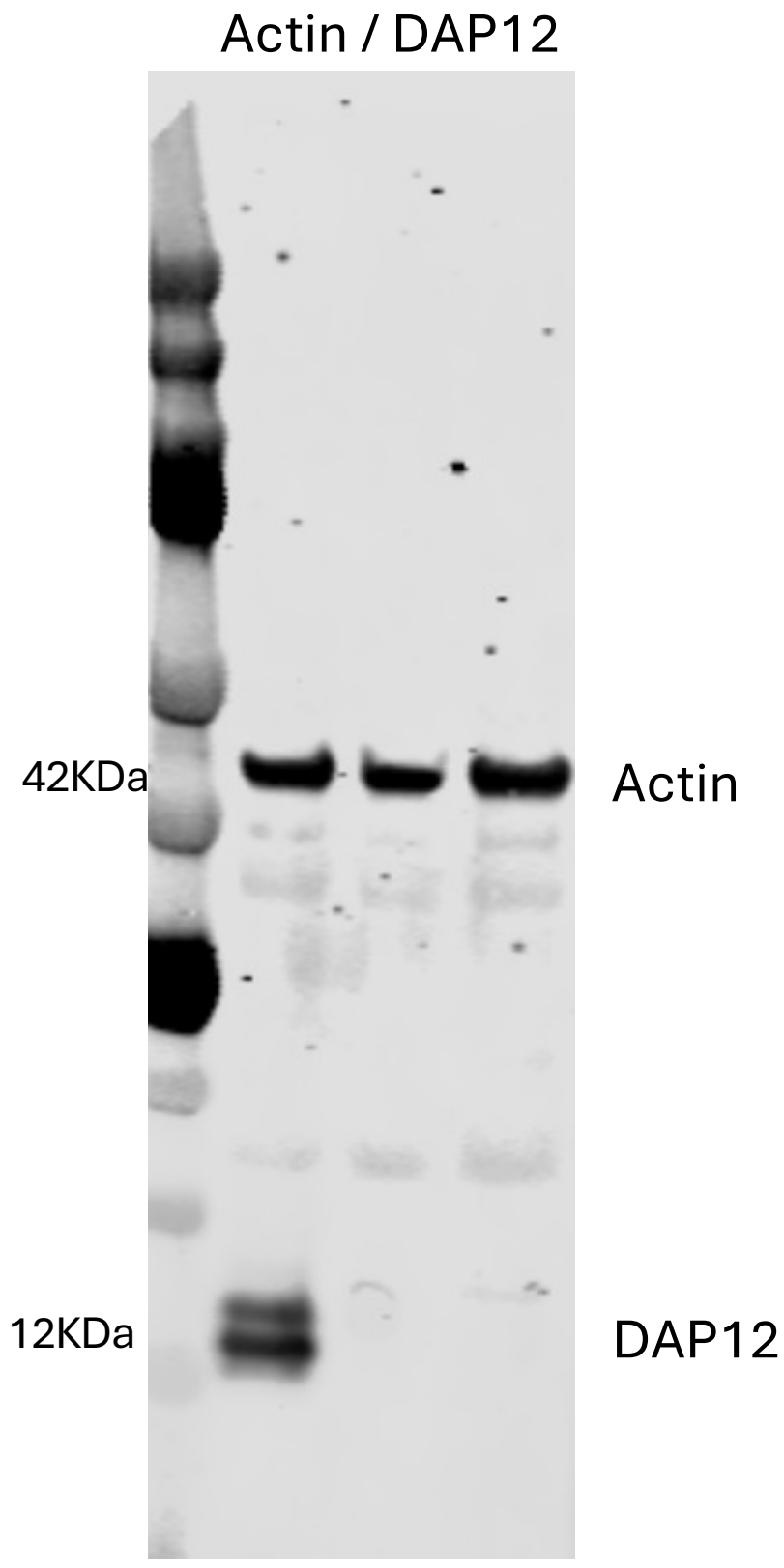
Actin / DAP12
42KDa
Actin
12KDa
DAP12

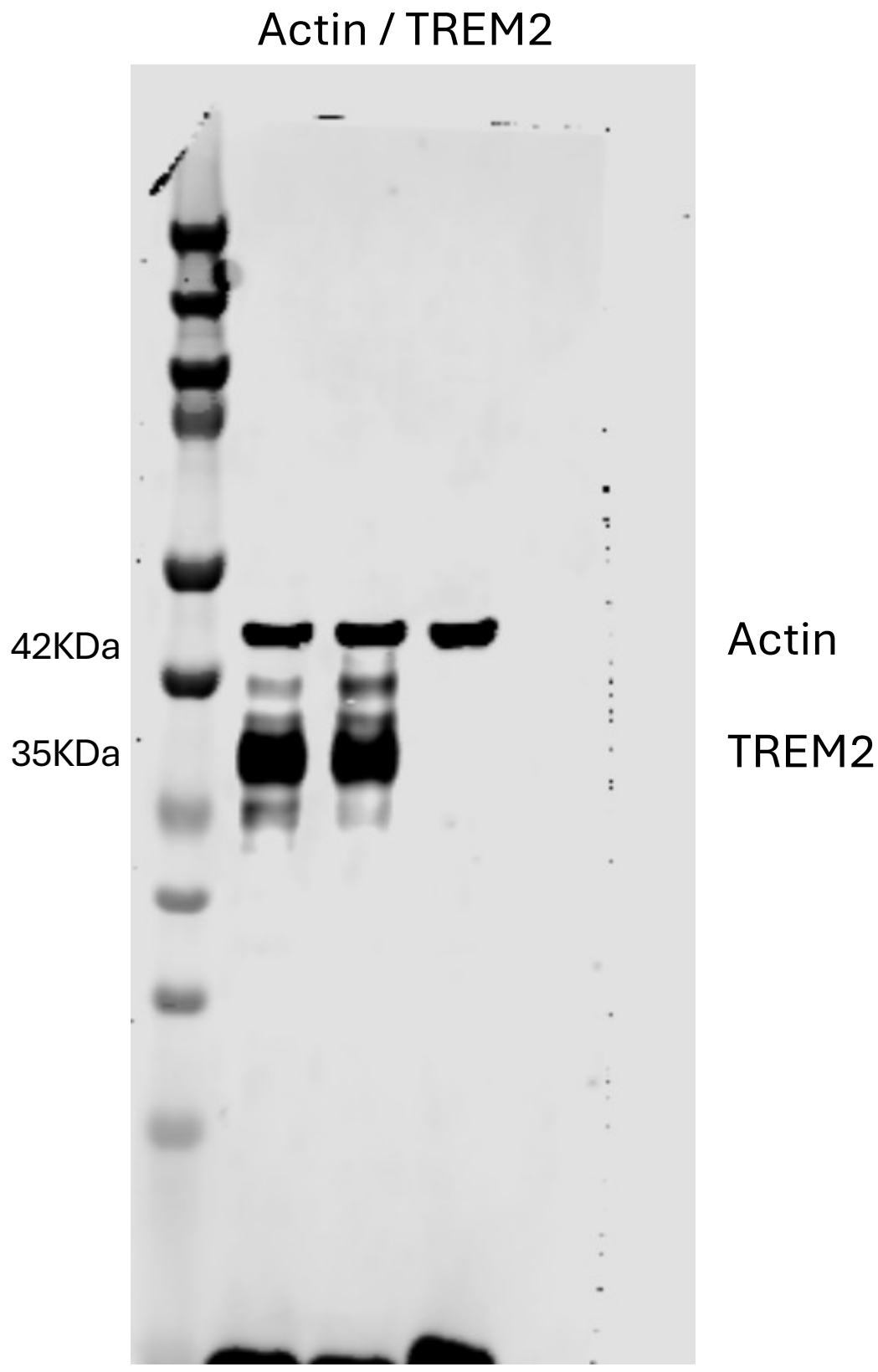
Actin / TREM2
42KDa
Actin
35KDa
TREM2

Fig7j Blot

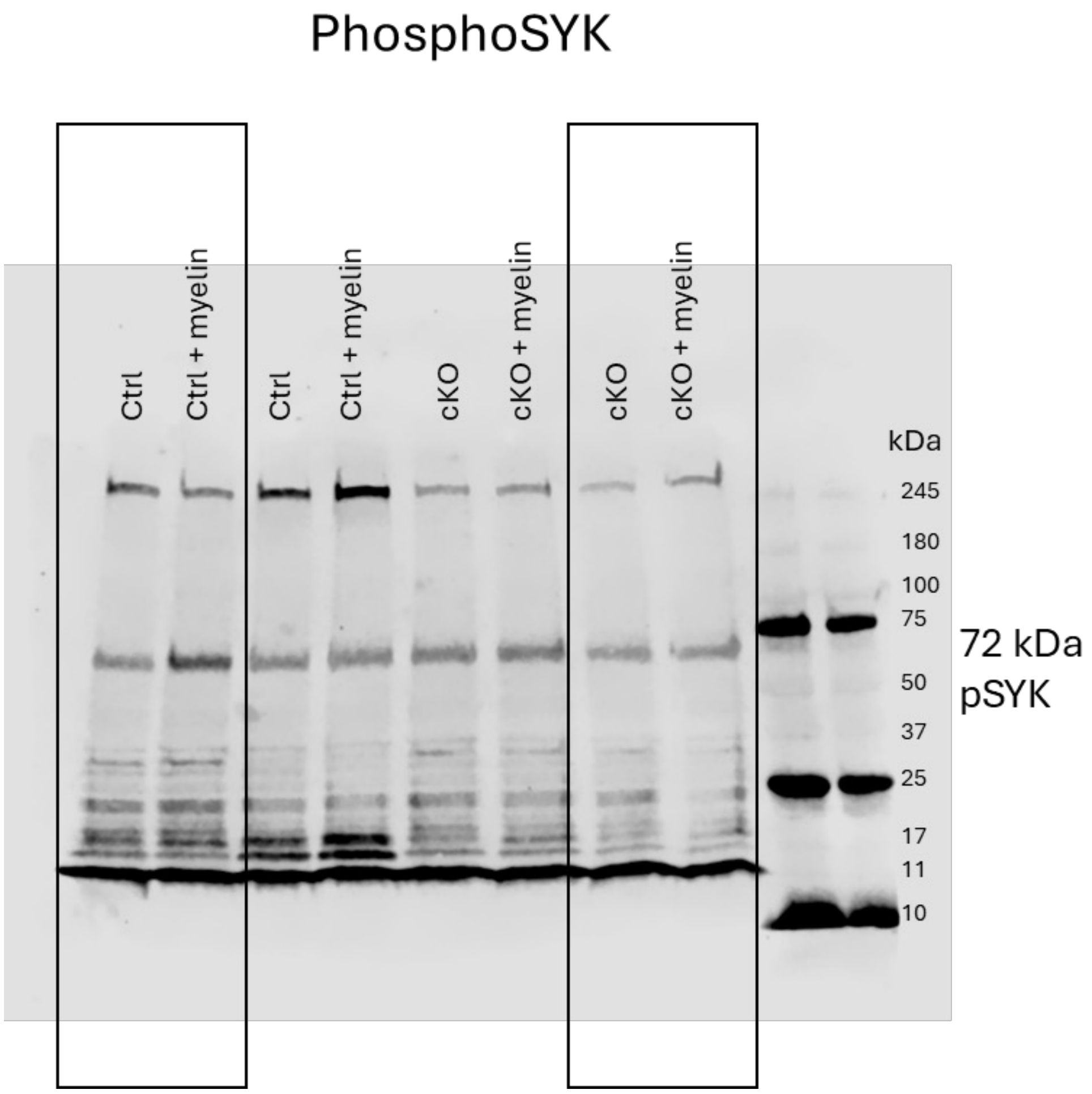

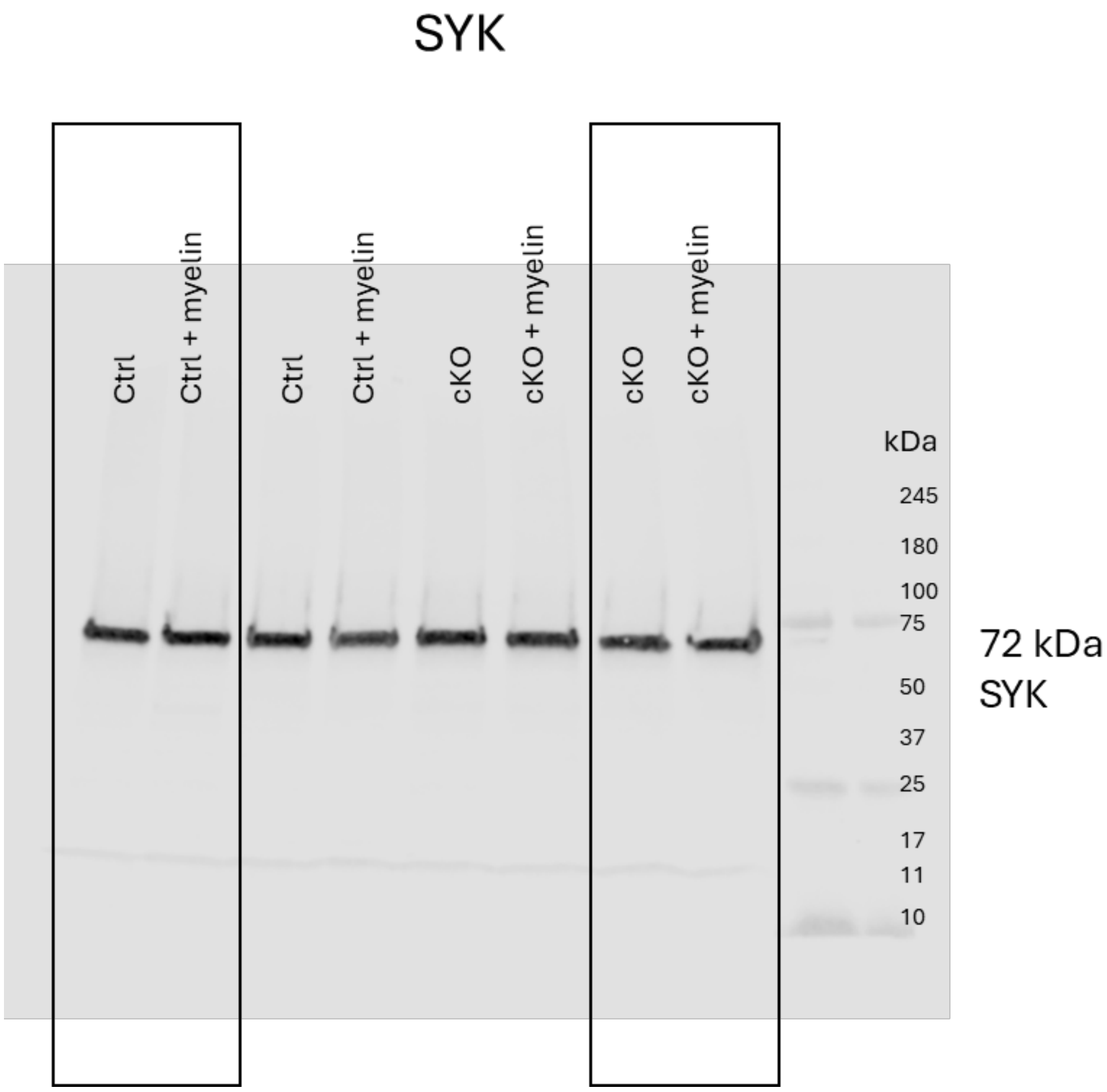

Shown in representative

Fig7m Blot

PhosphoSYK

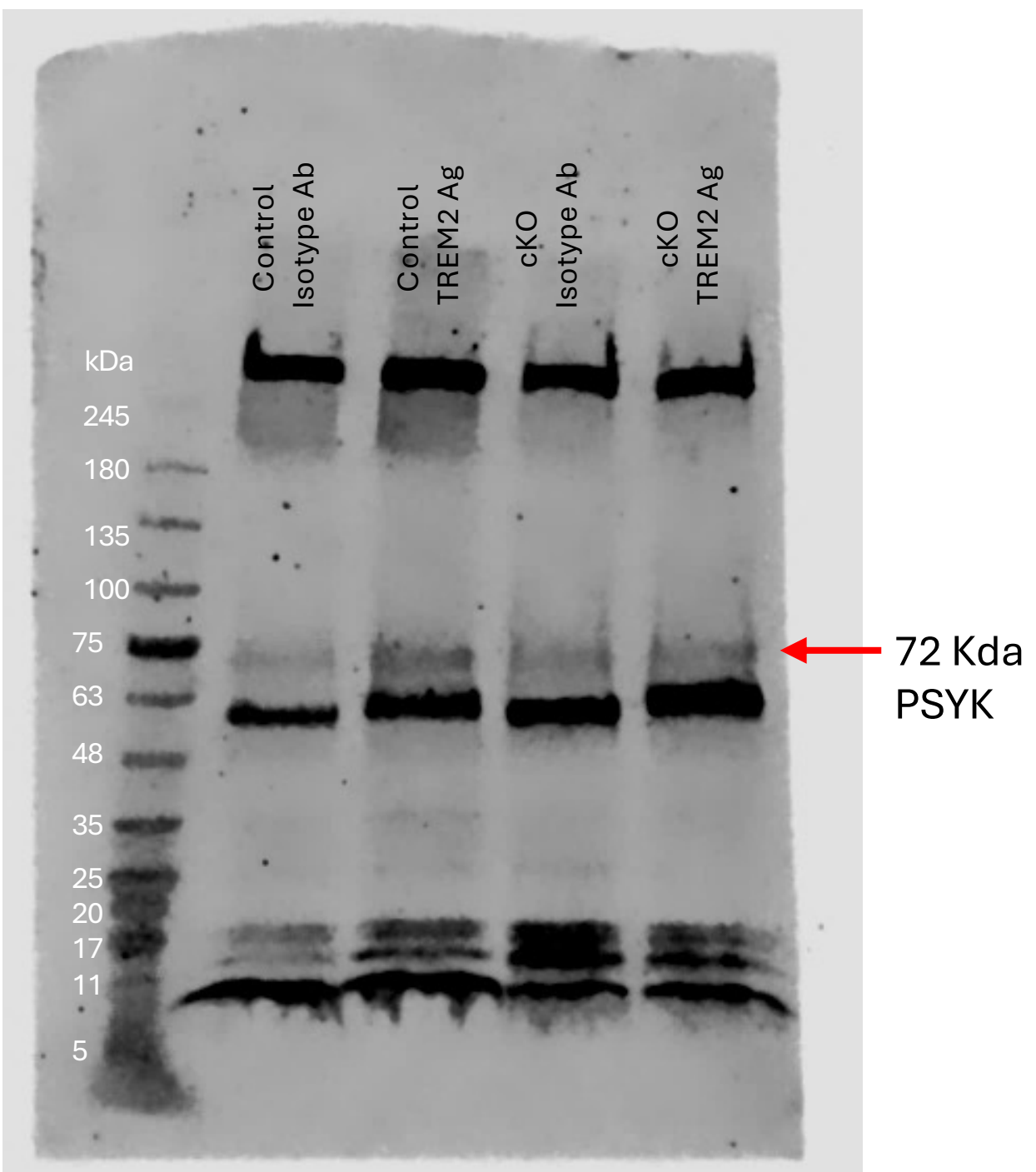

SYK

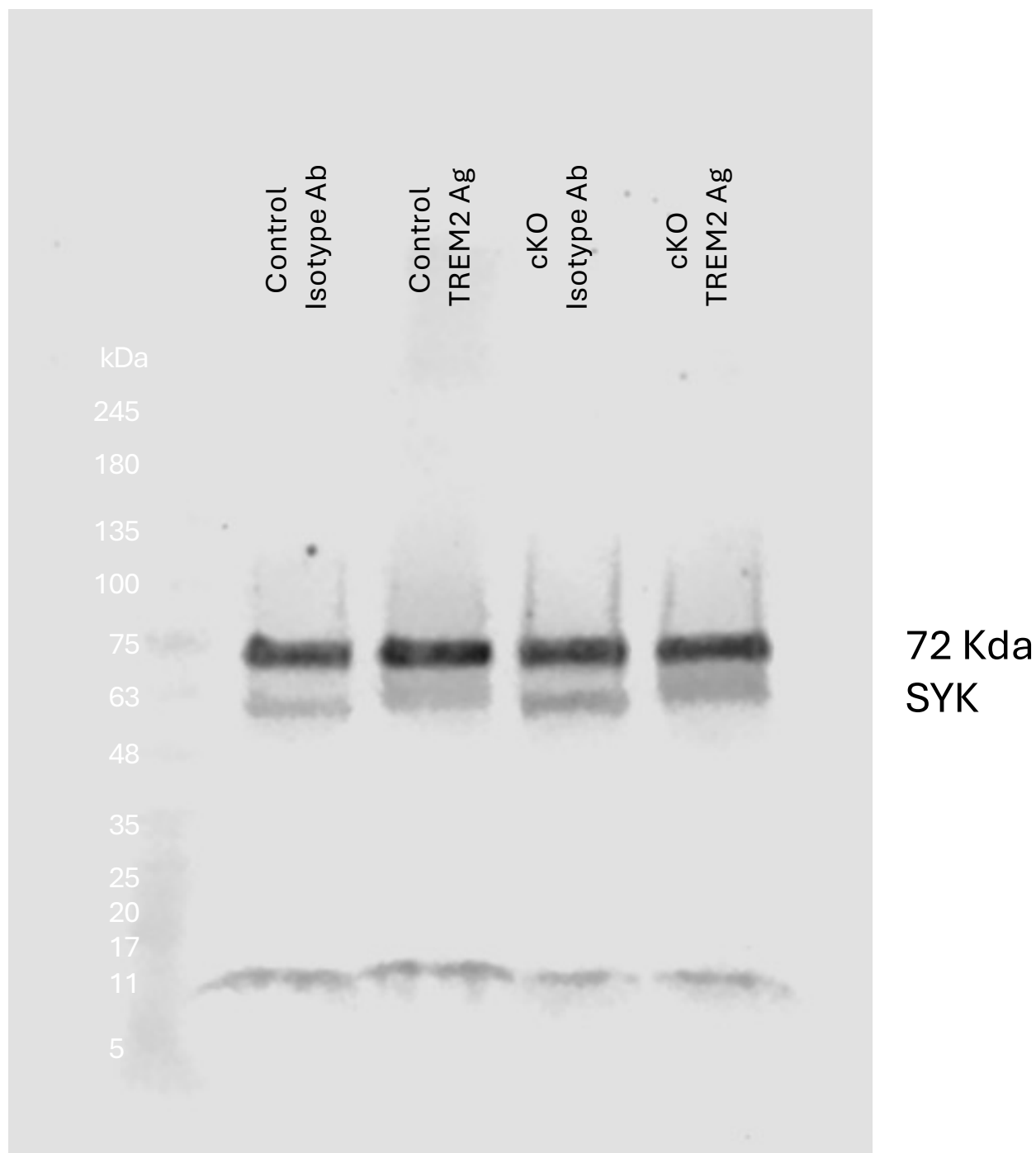

SupFig2b Blot

Total protein

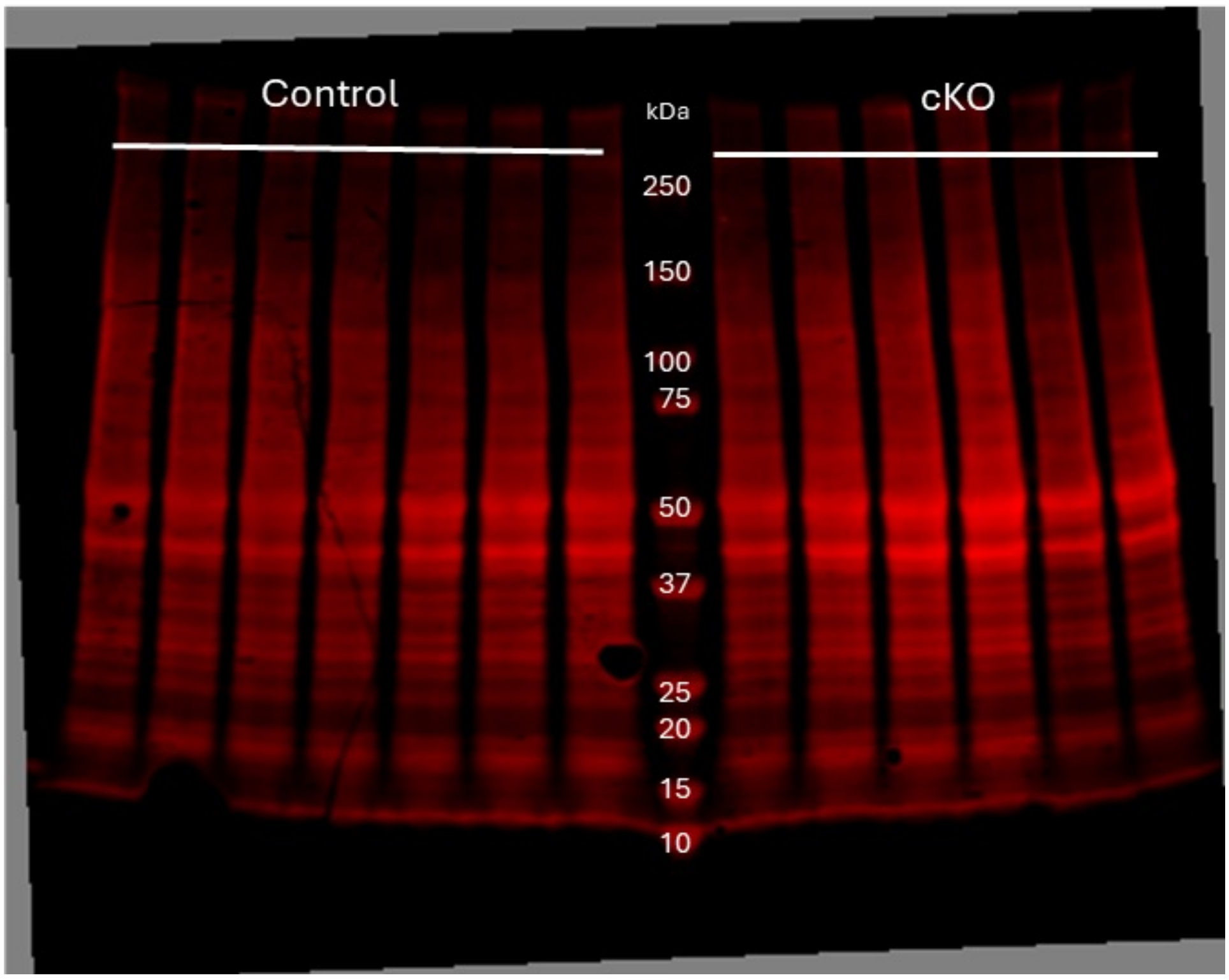

MBP

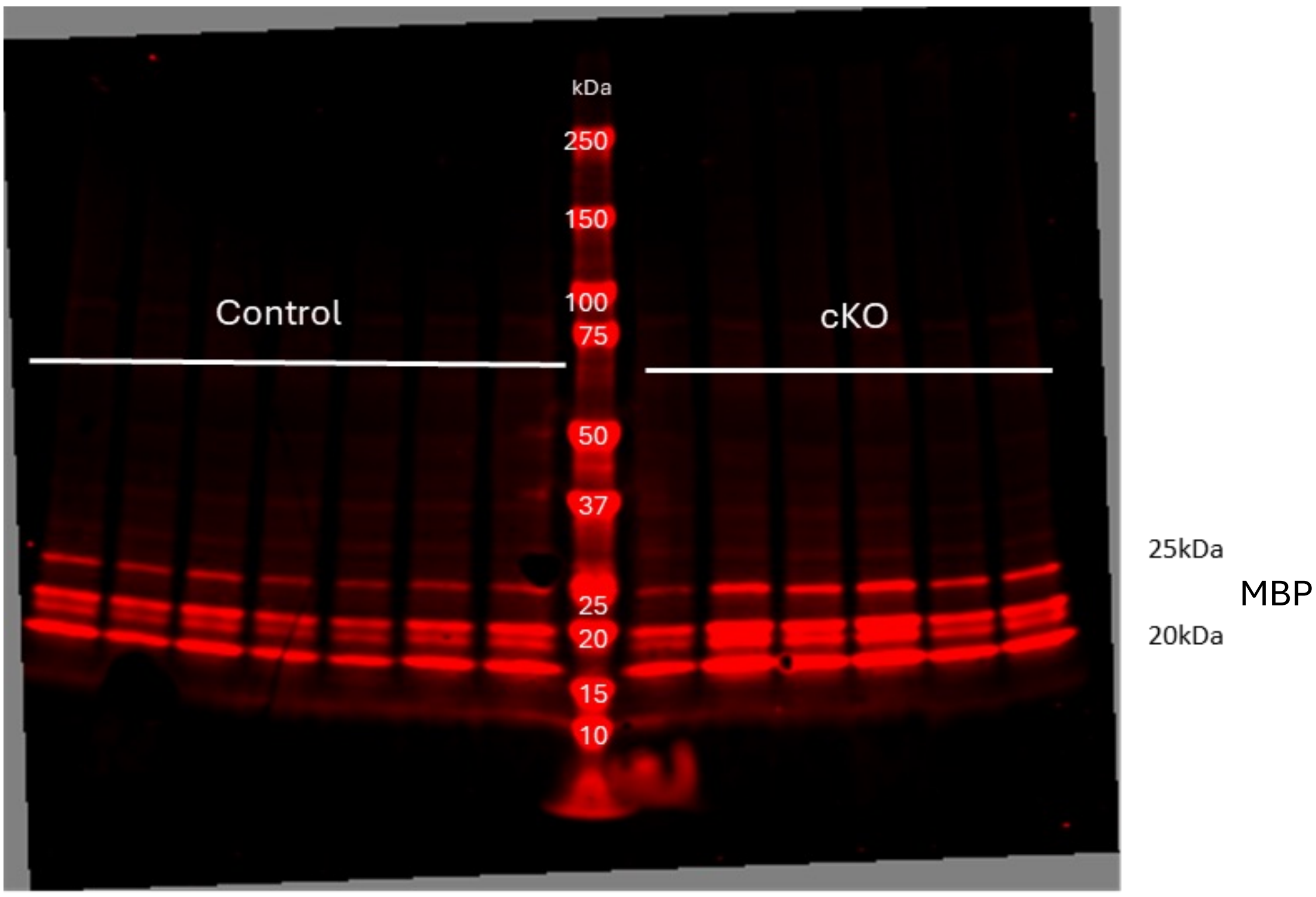

SupFig3c Blot

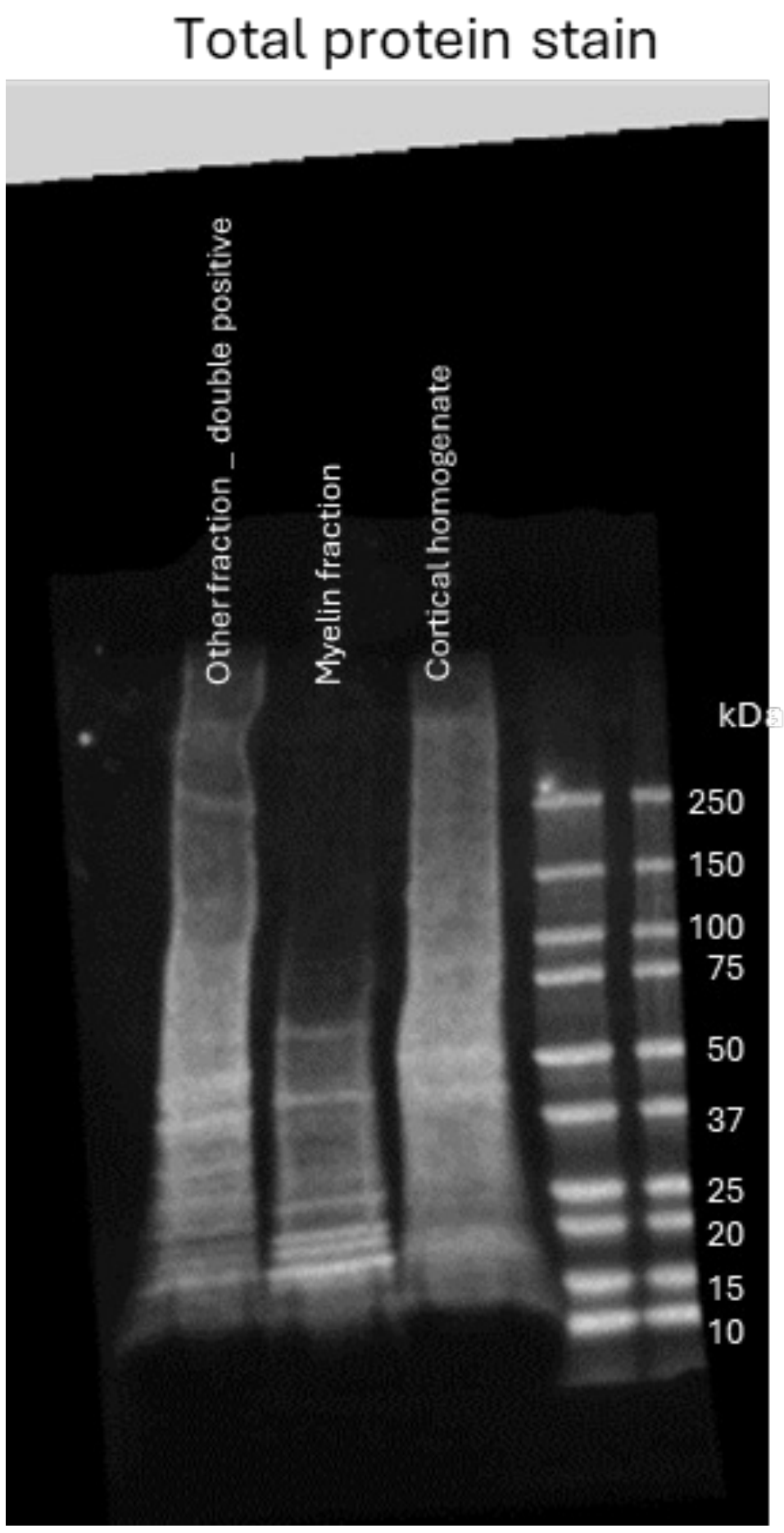

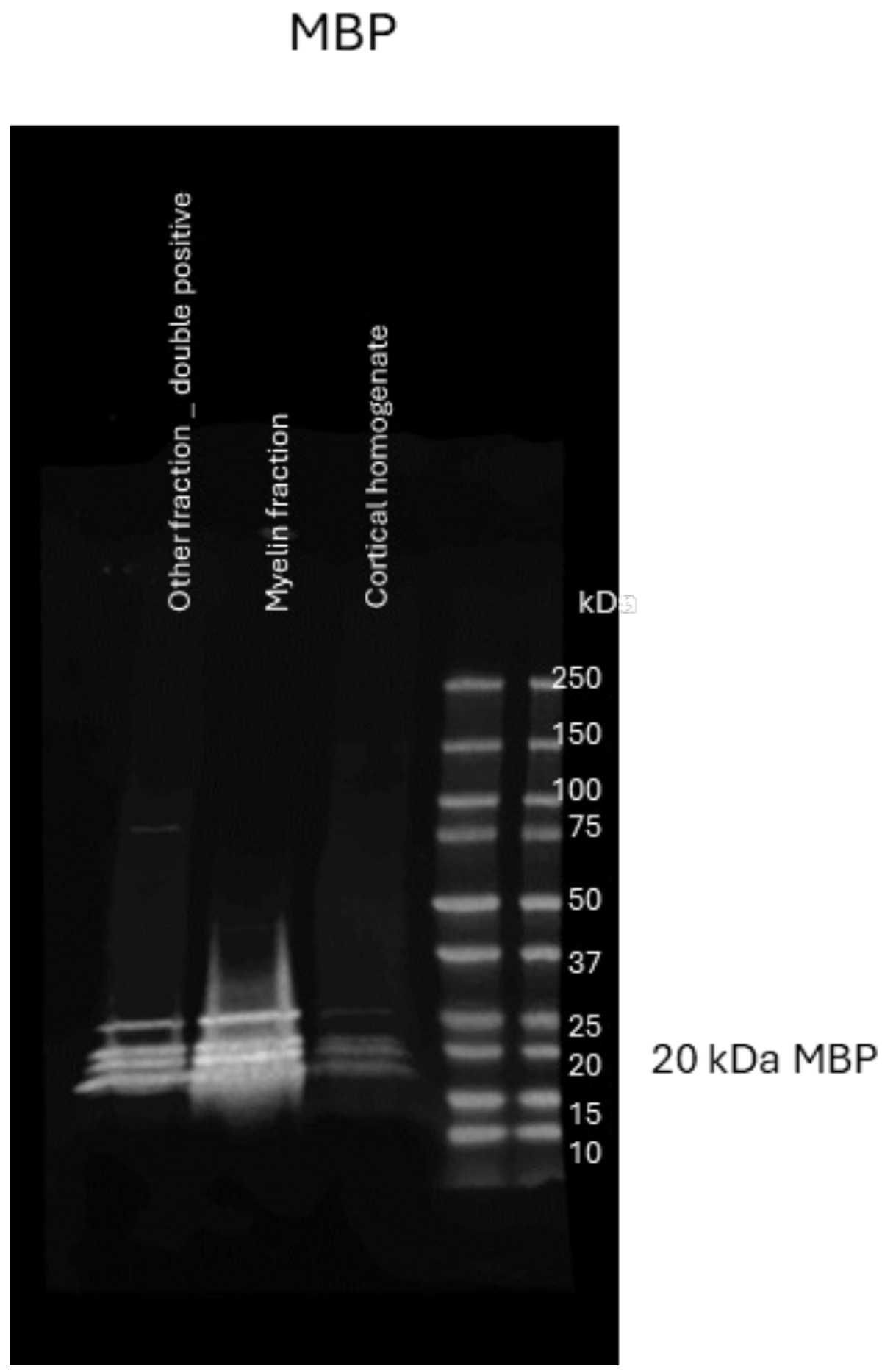

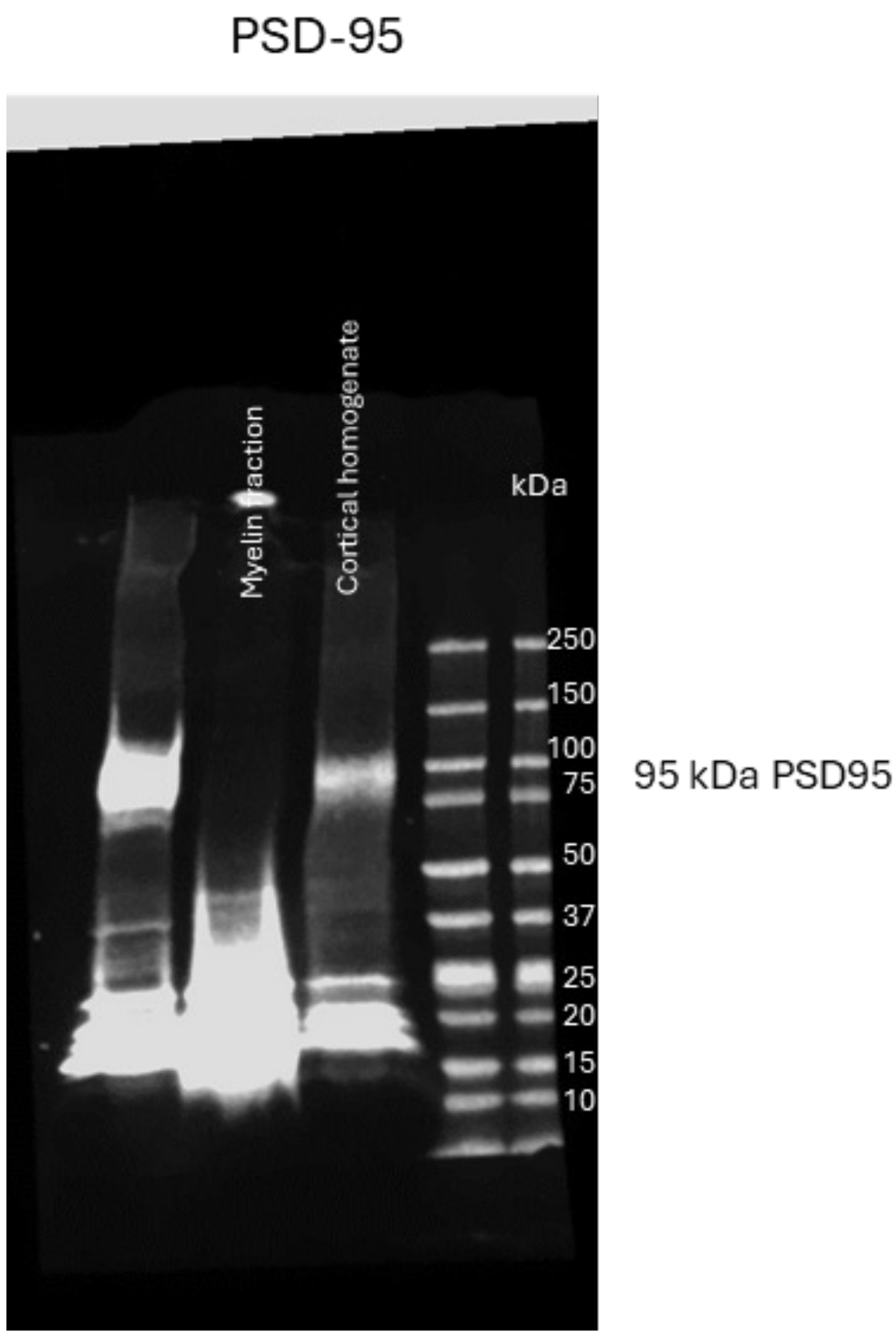

SupFig4 Blot

Actin-GAPDH

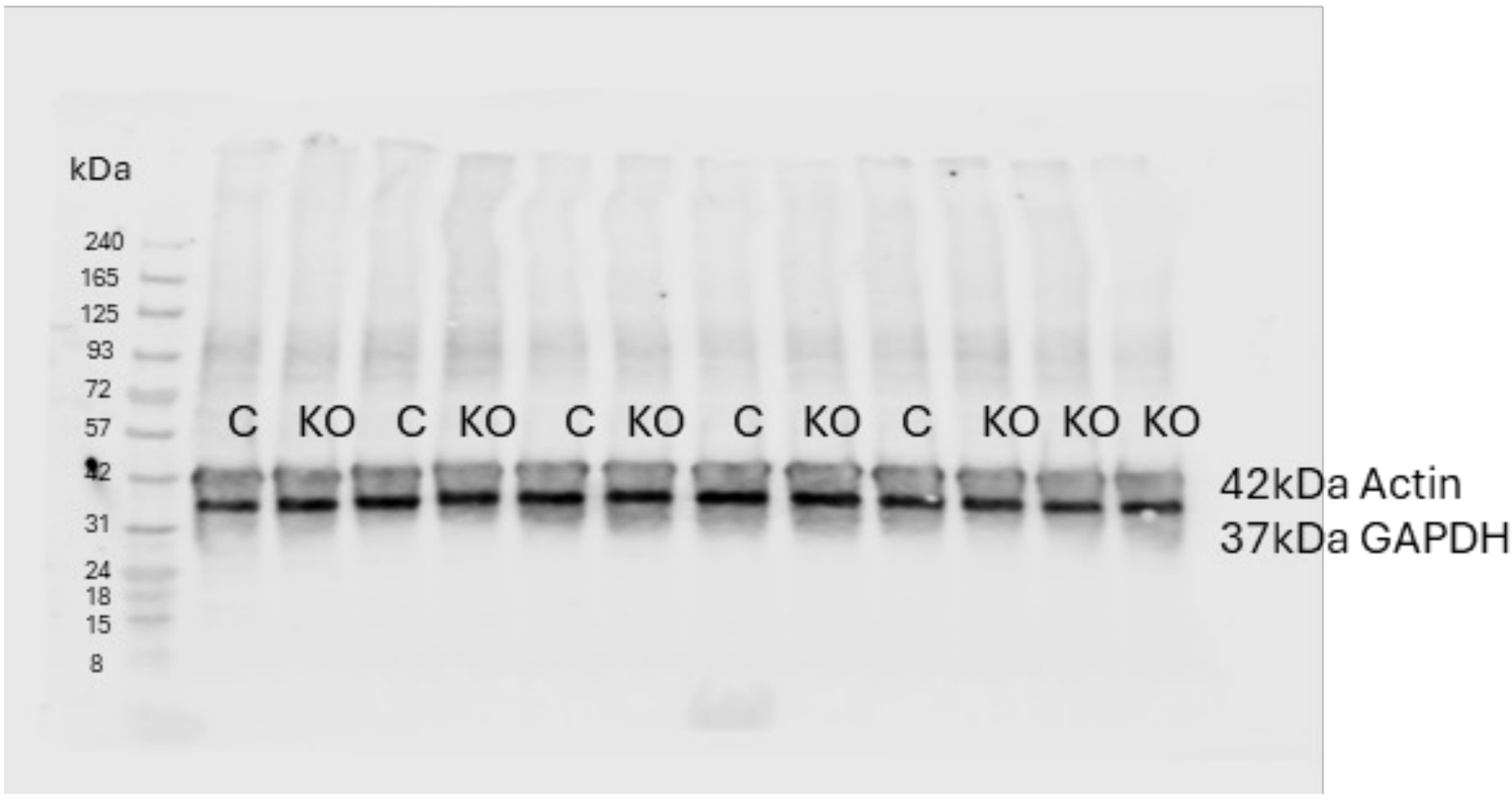

MBP

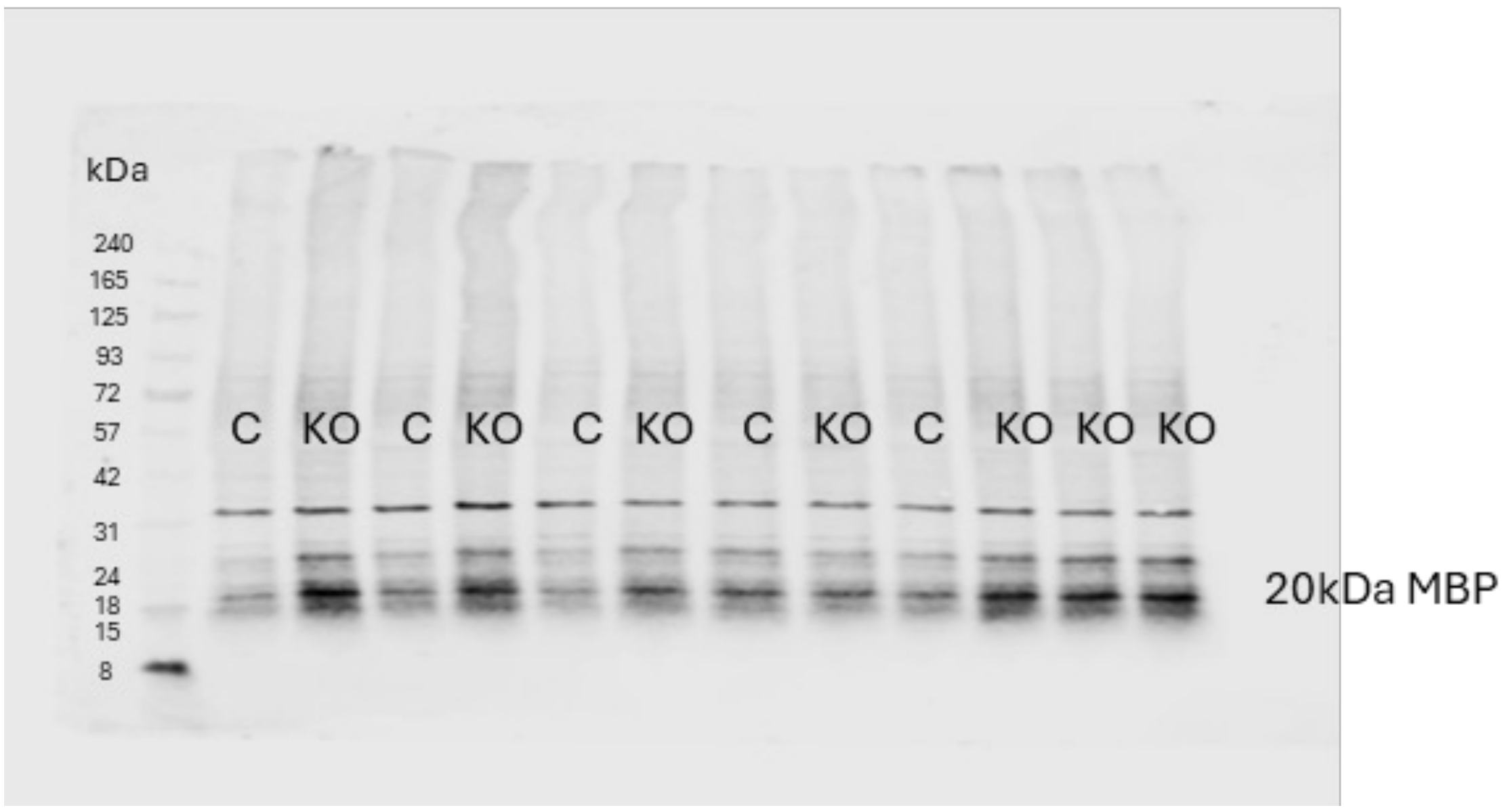

SupFig4w Blot

Actin-GAPDH

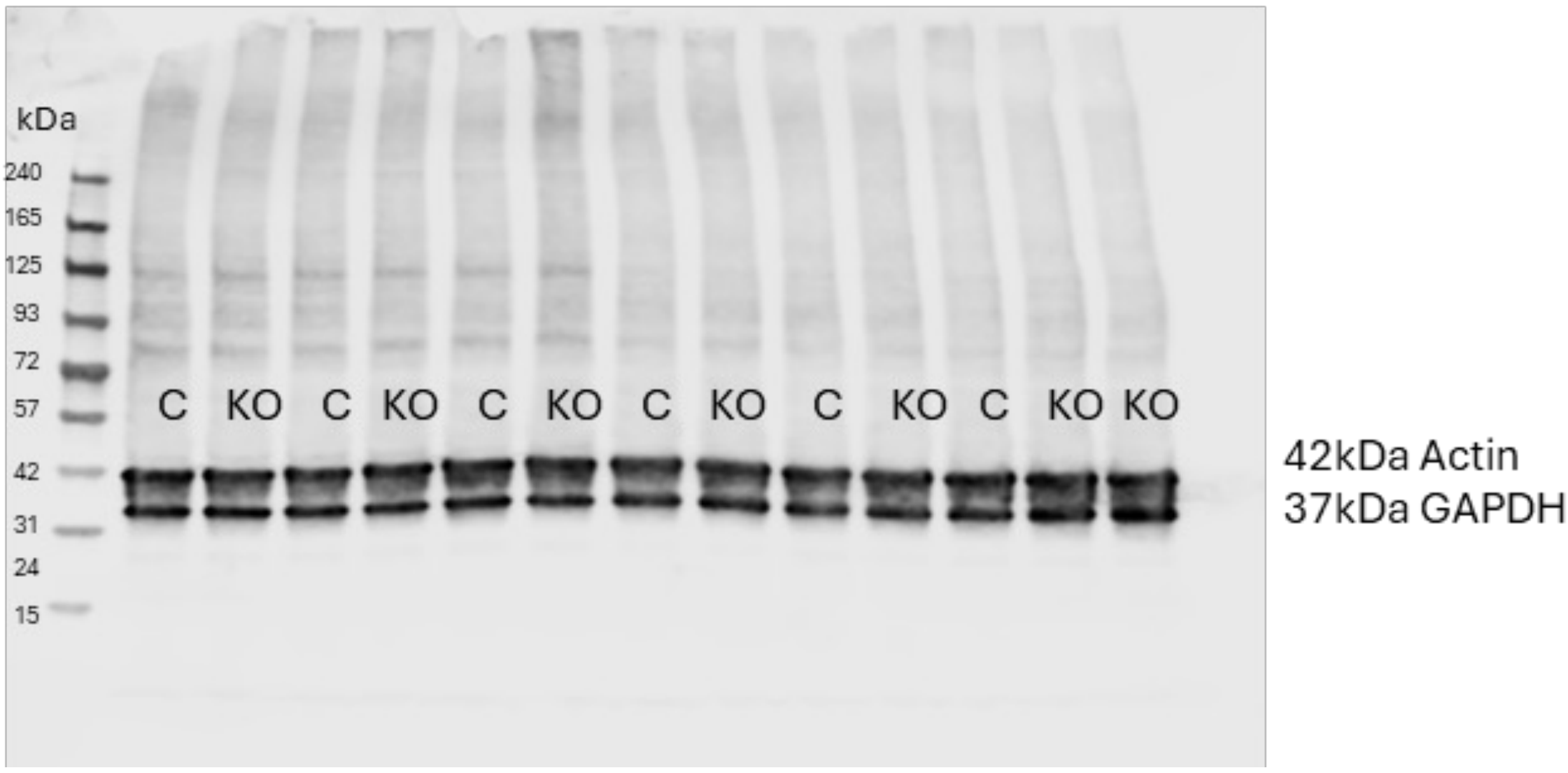

MBP

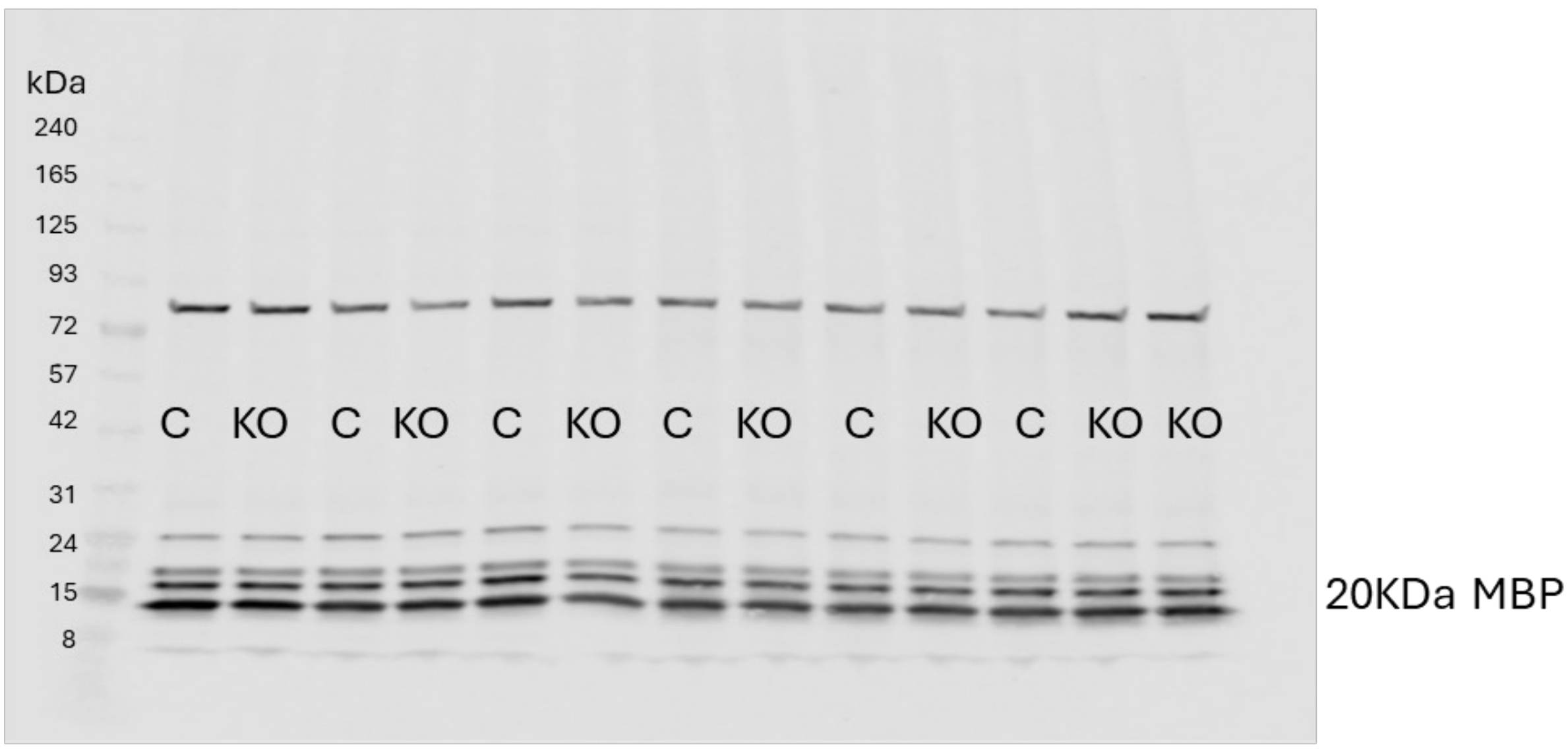

SupFig5j Blot

Total protein stain

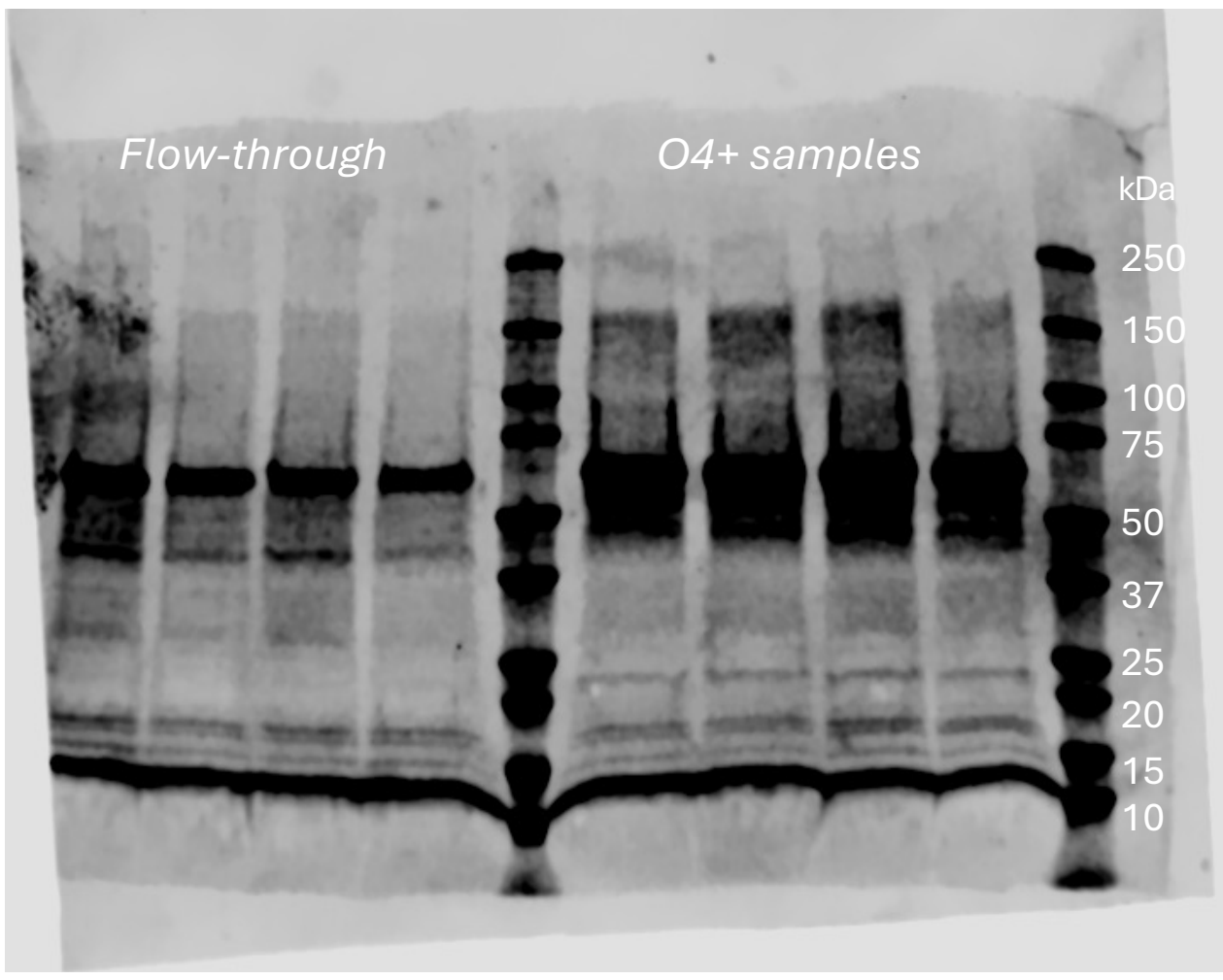

OLIG2

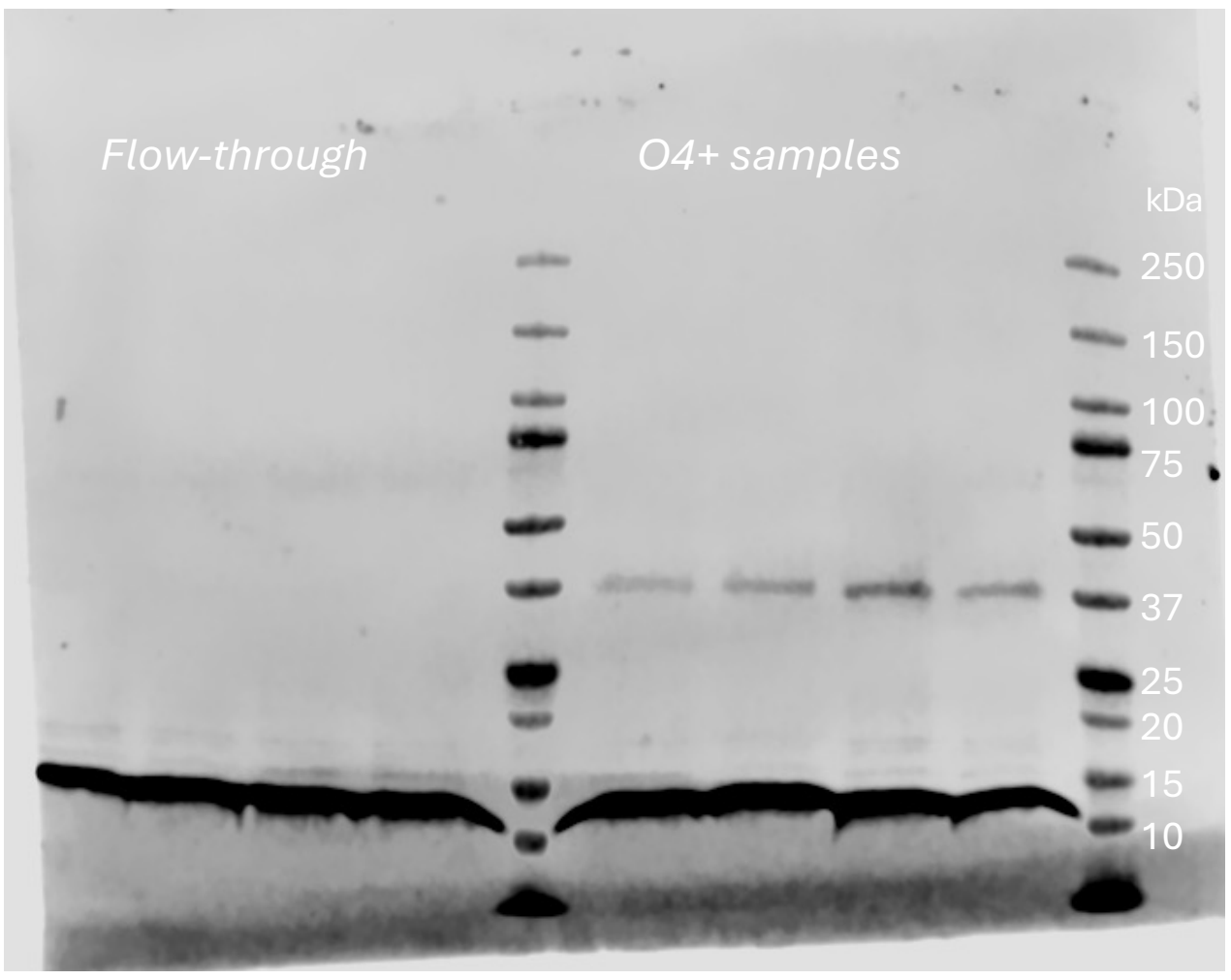

37kDa OLIG2

IBA1

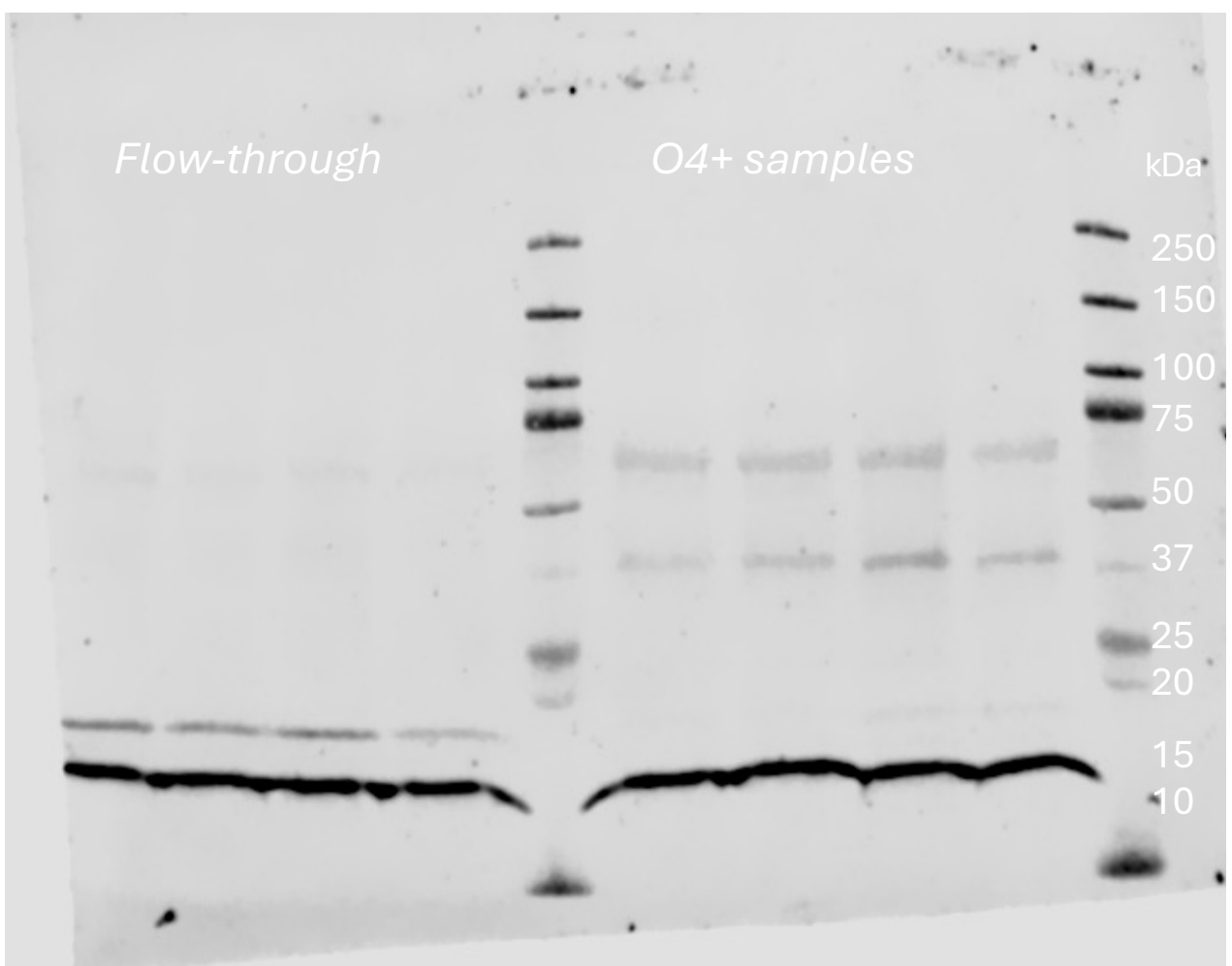

17kDa IBA1

SupFig6i Blot

Total protein stain

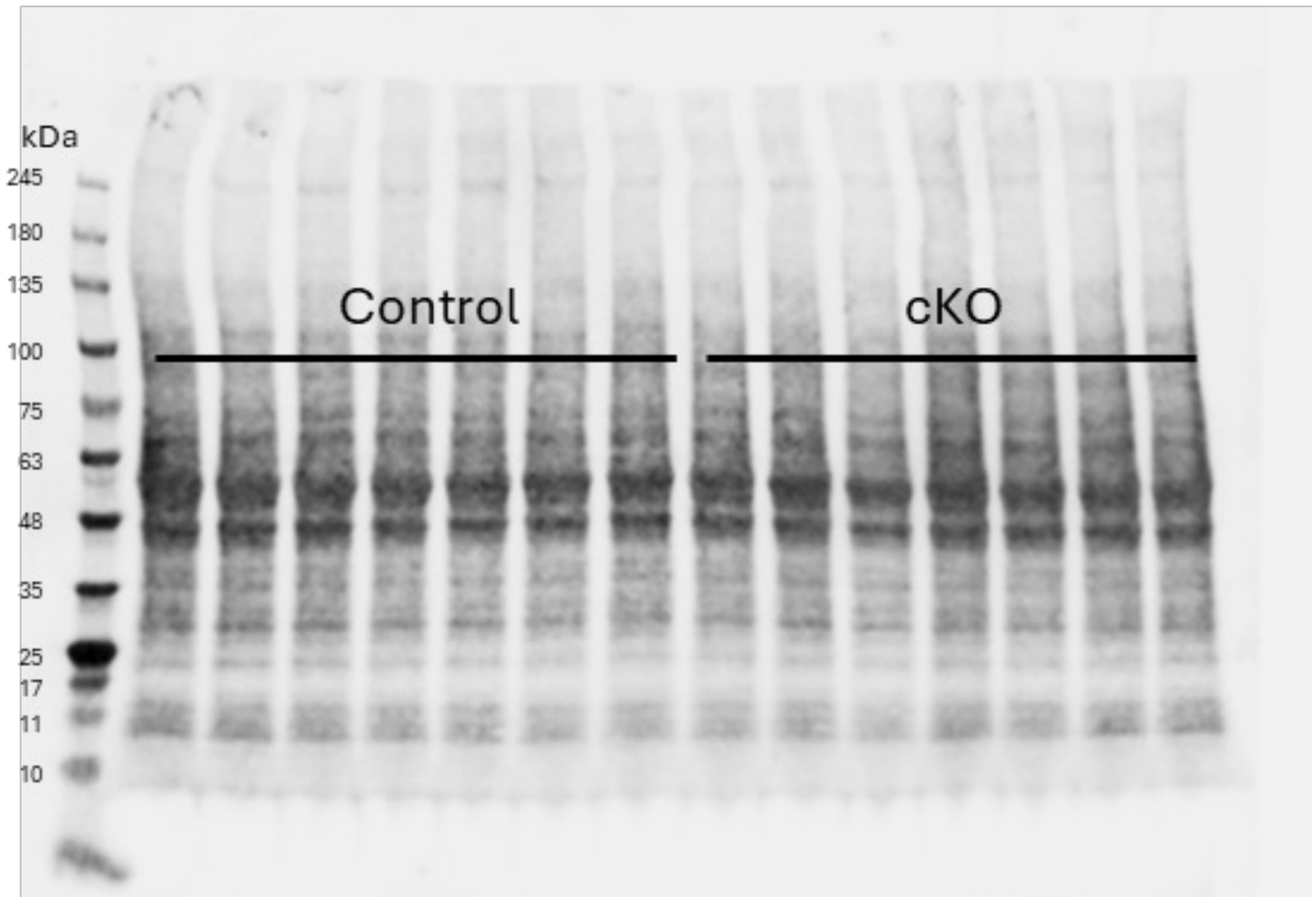

DAP12

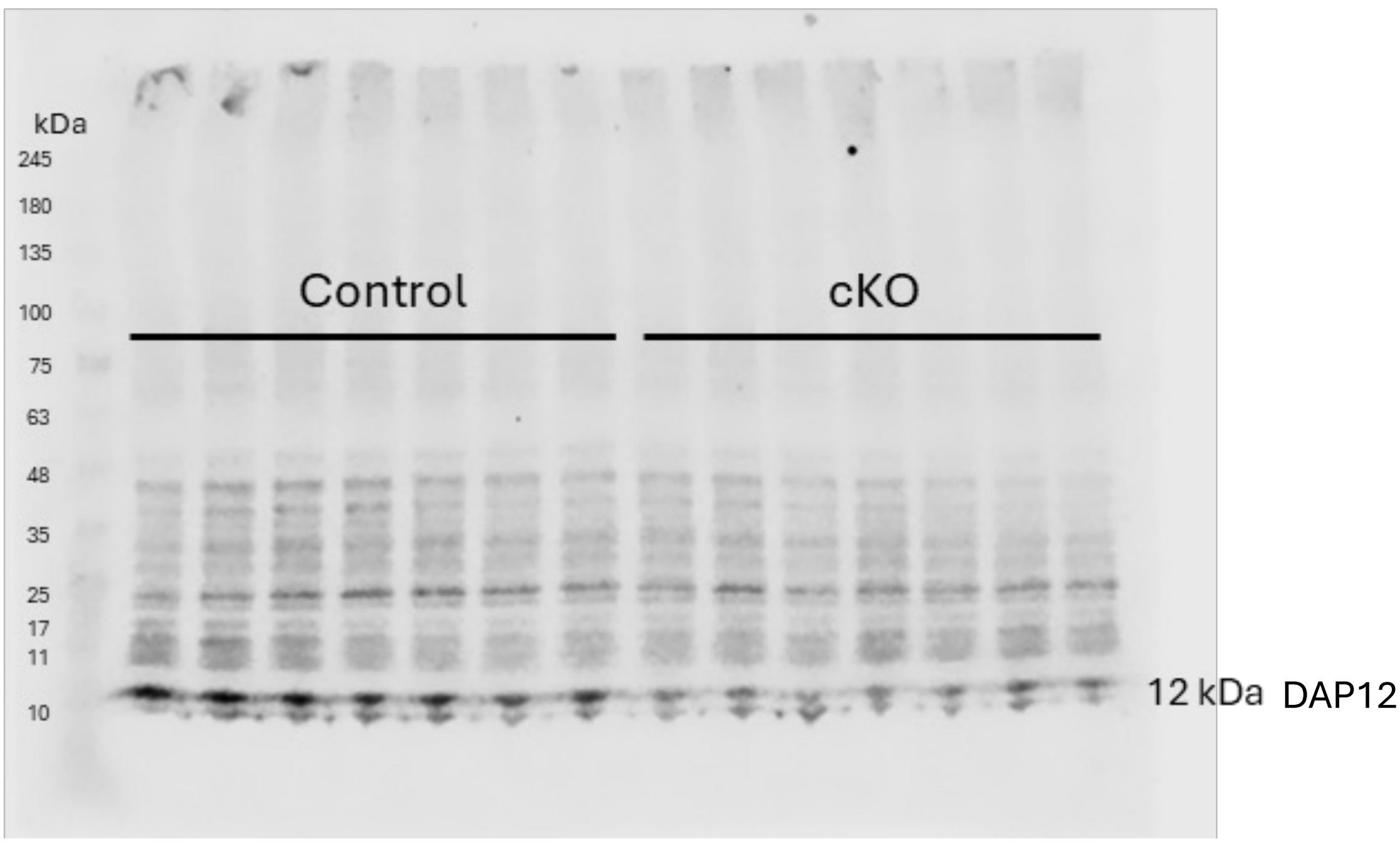

SupFig6k Blot

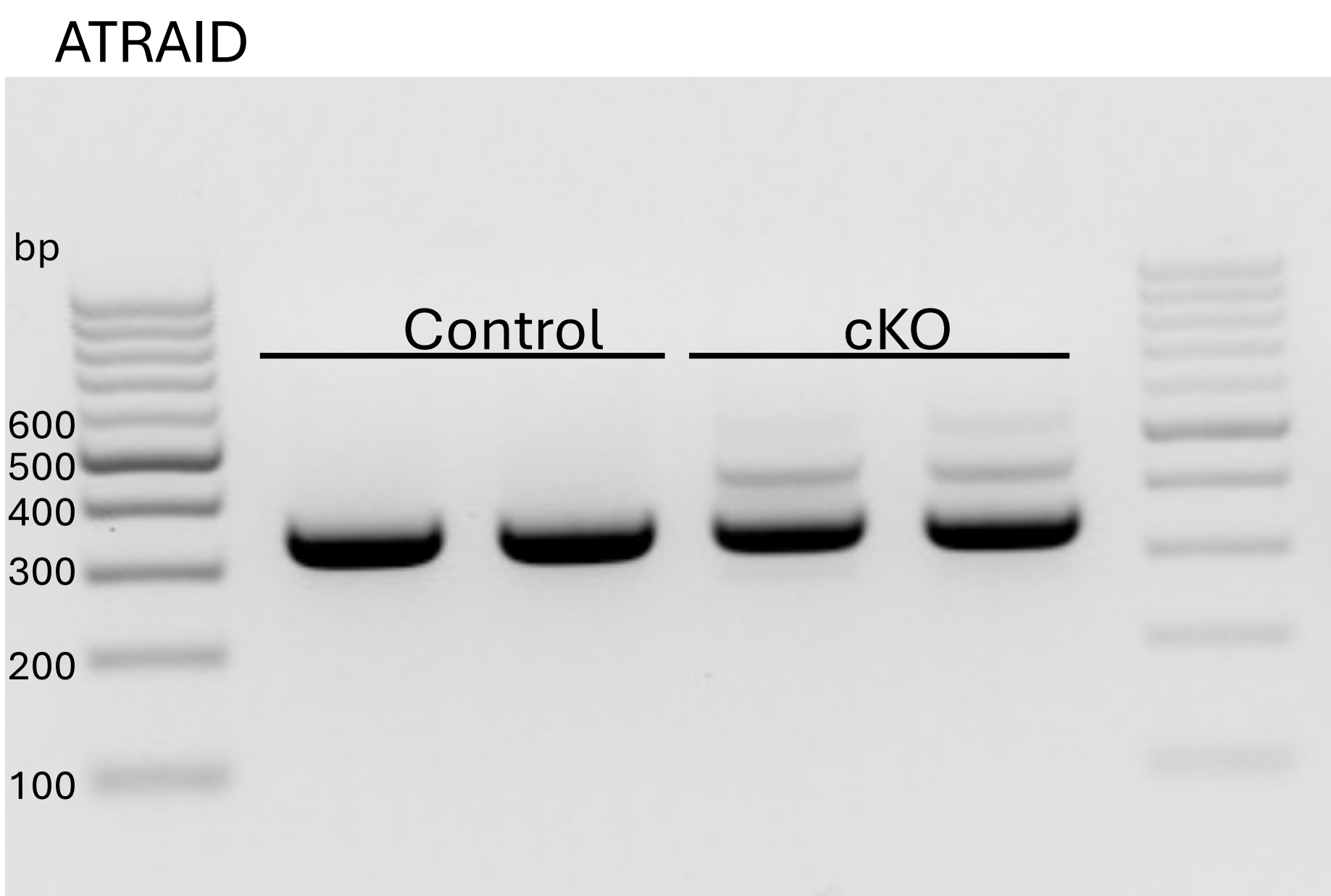

SupFig6n Blot

Total protein

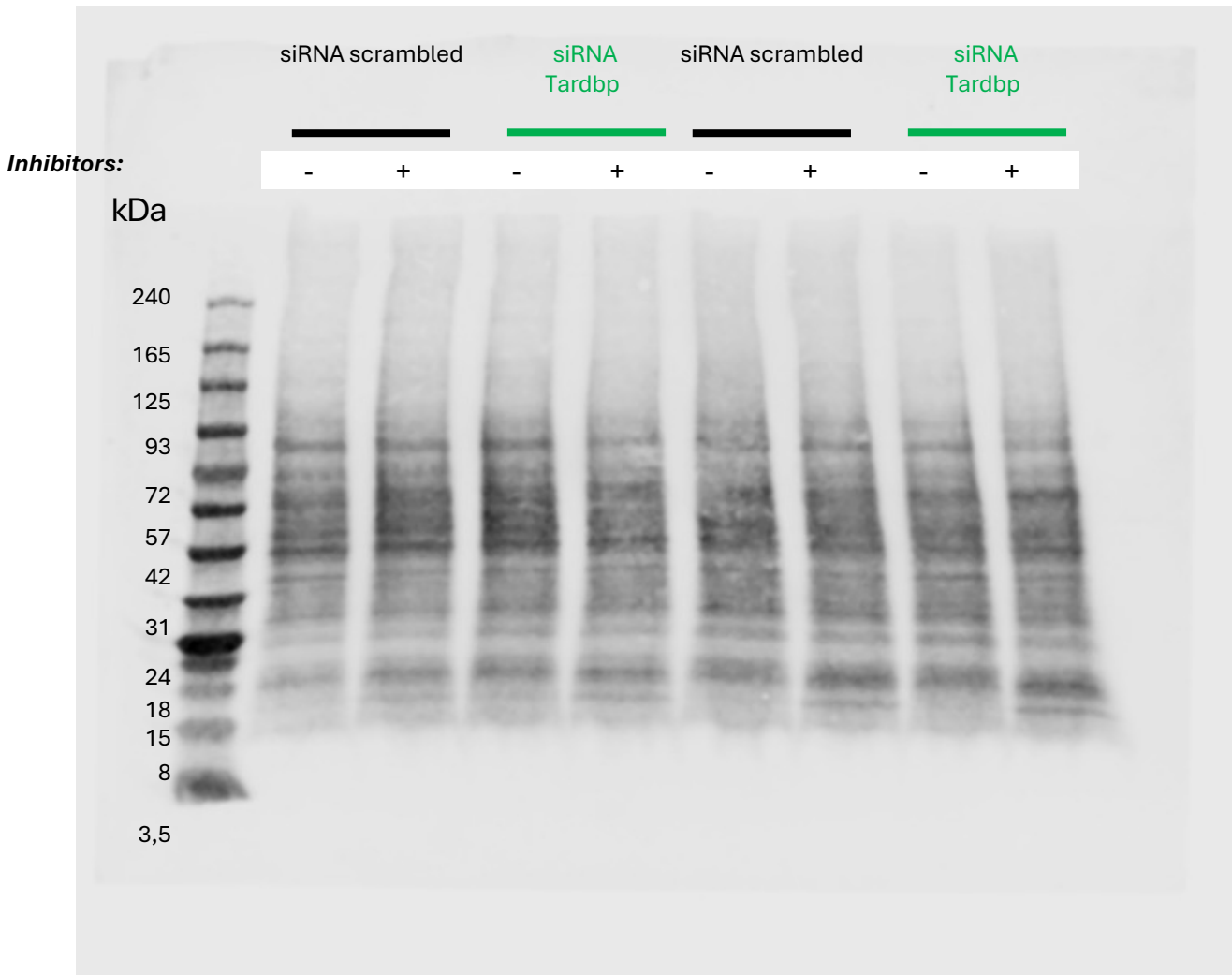

ubiquitin

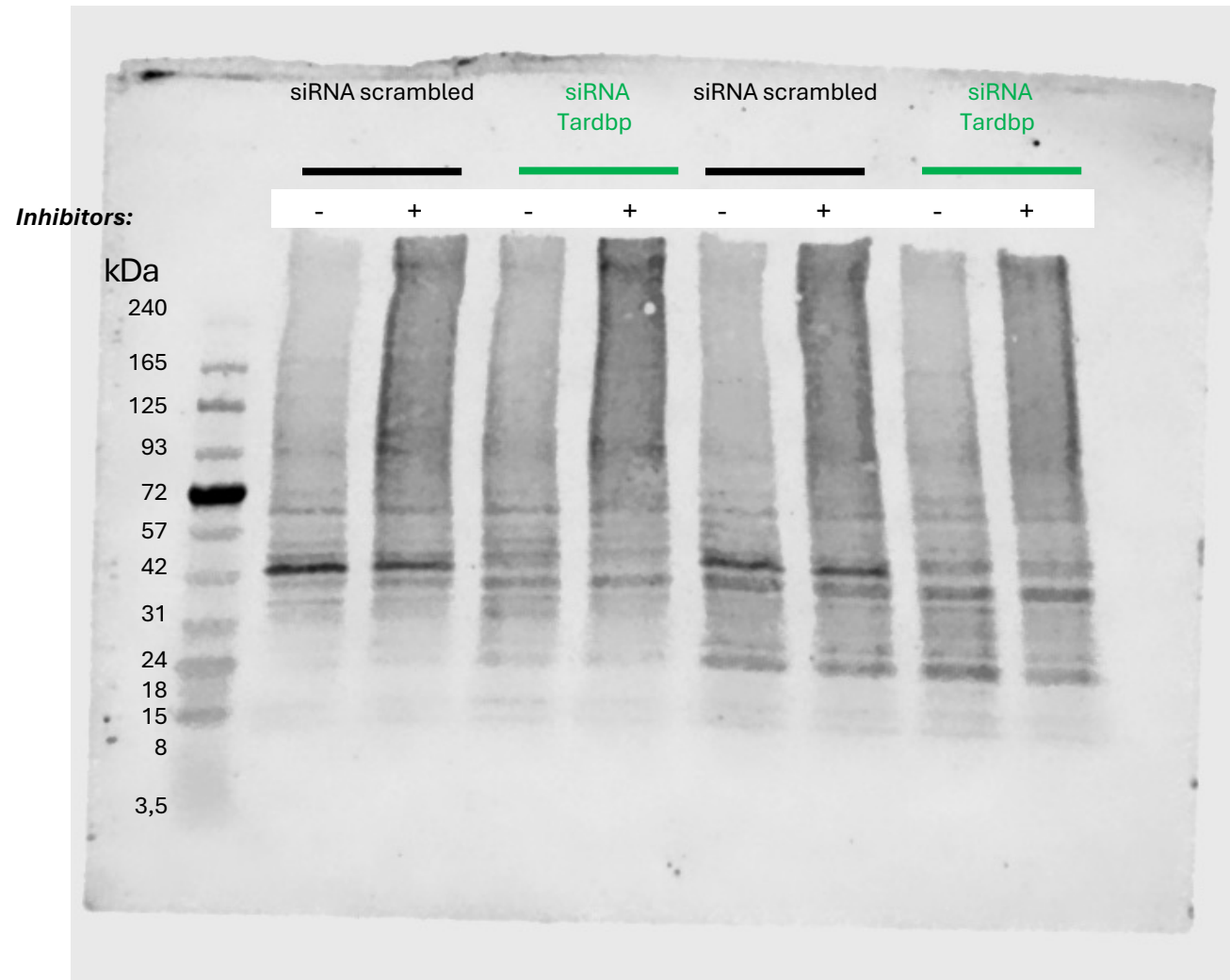

TDP-43

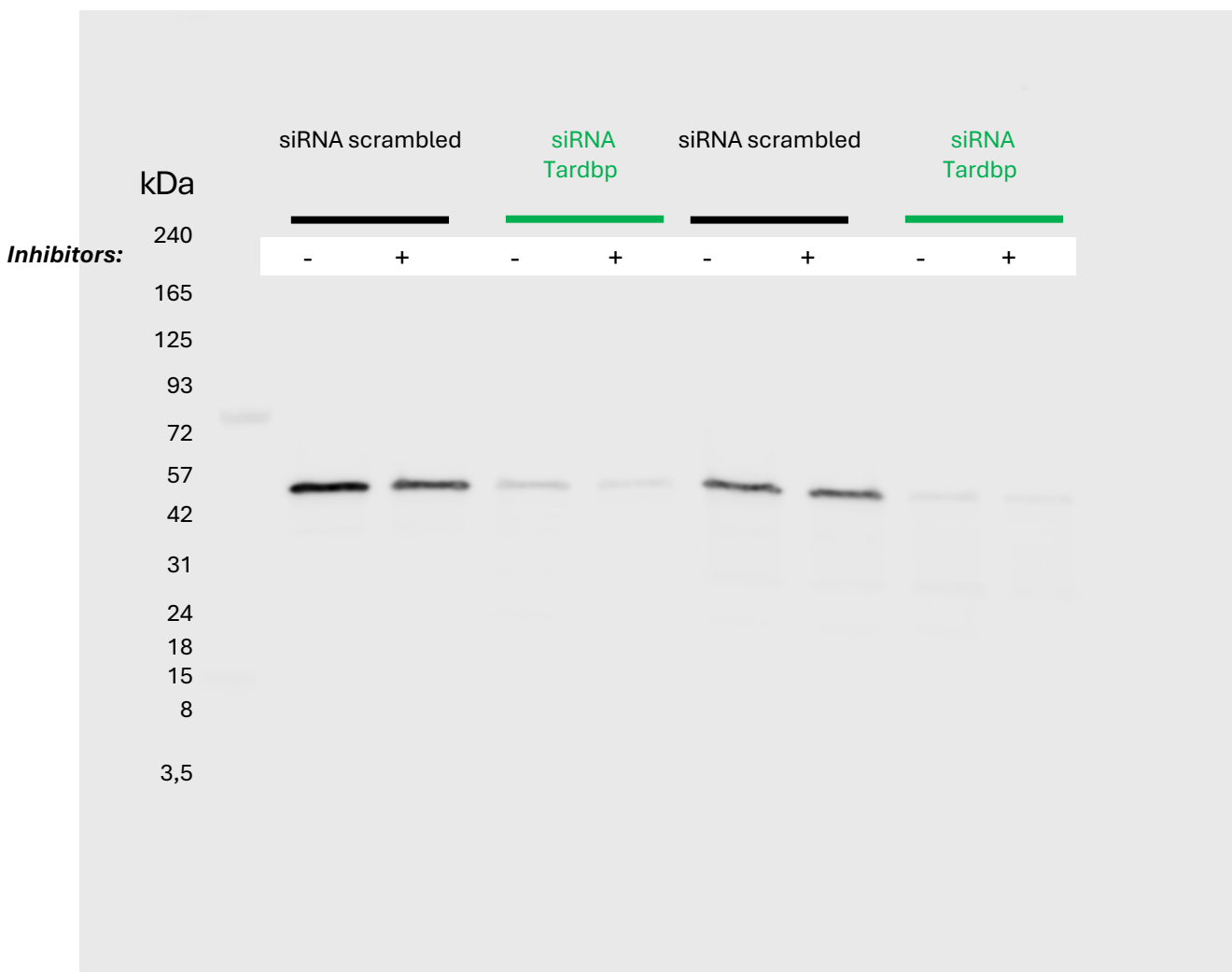

43 kDa
TDP-43

DAP12

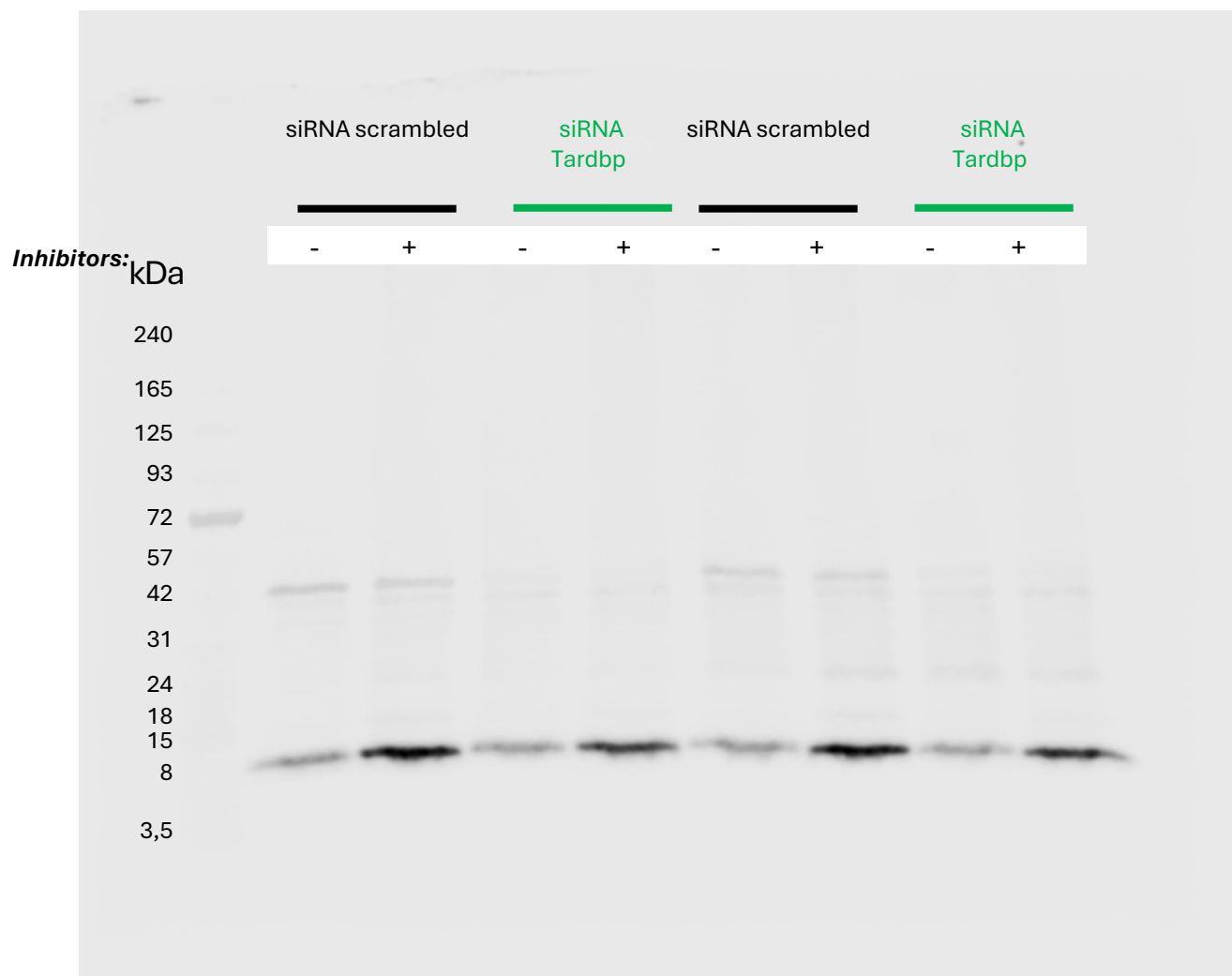

12 kDa
DAP12

SupFig6q Blot

Tyrobp RT-PCR amplification

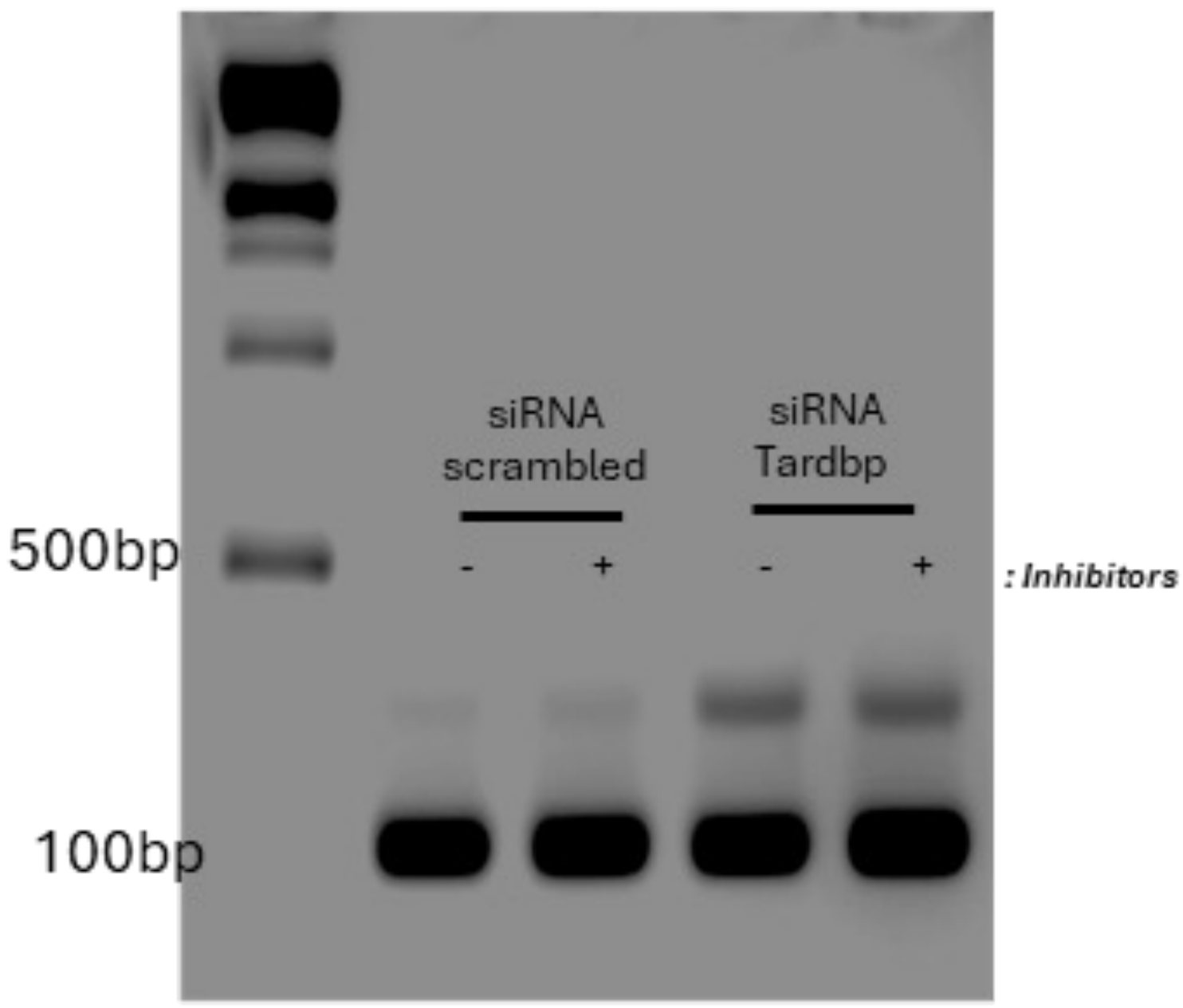

SupFig6b Blot

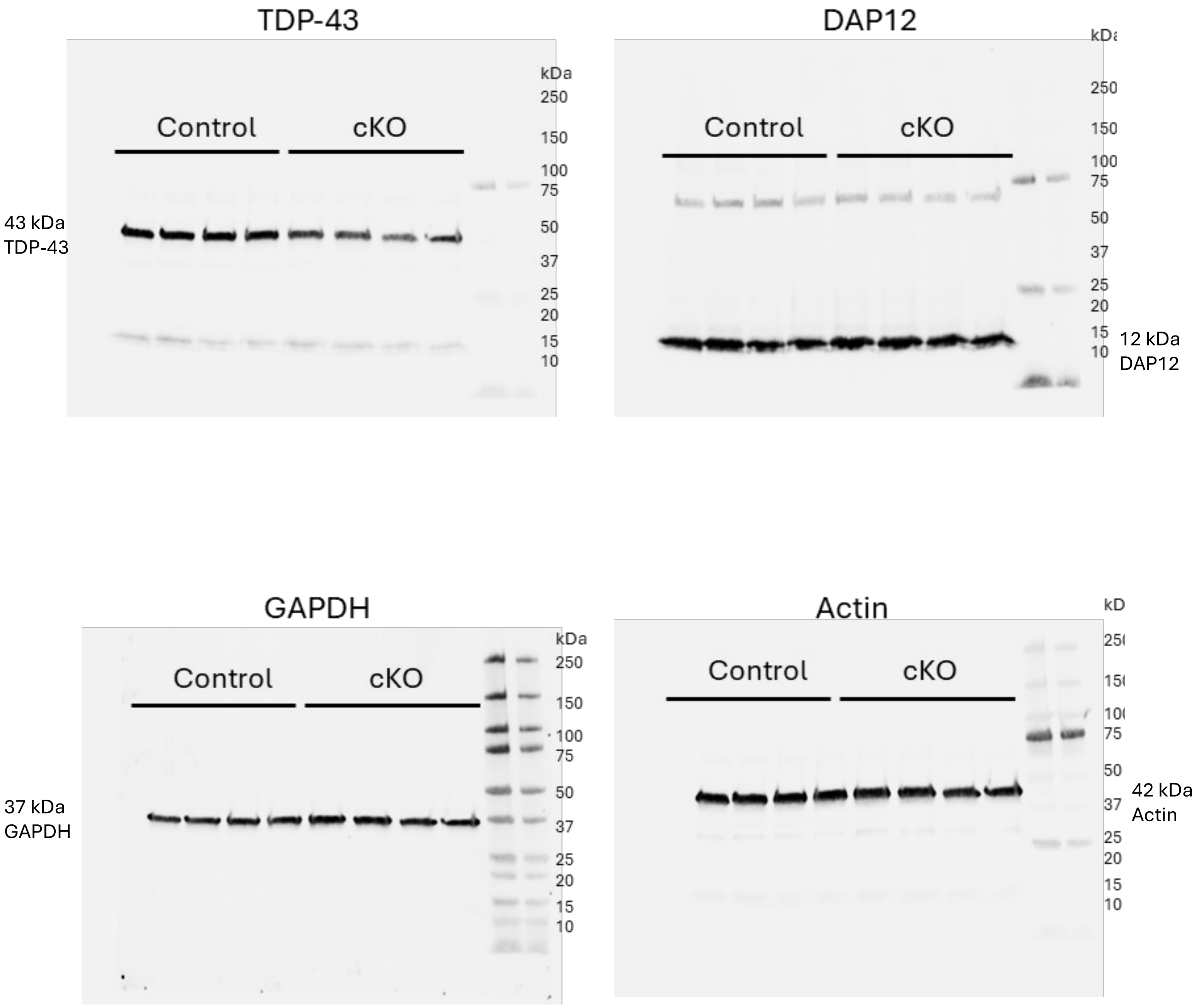

SupFig6f Blot

SYK

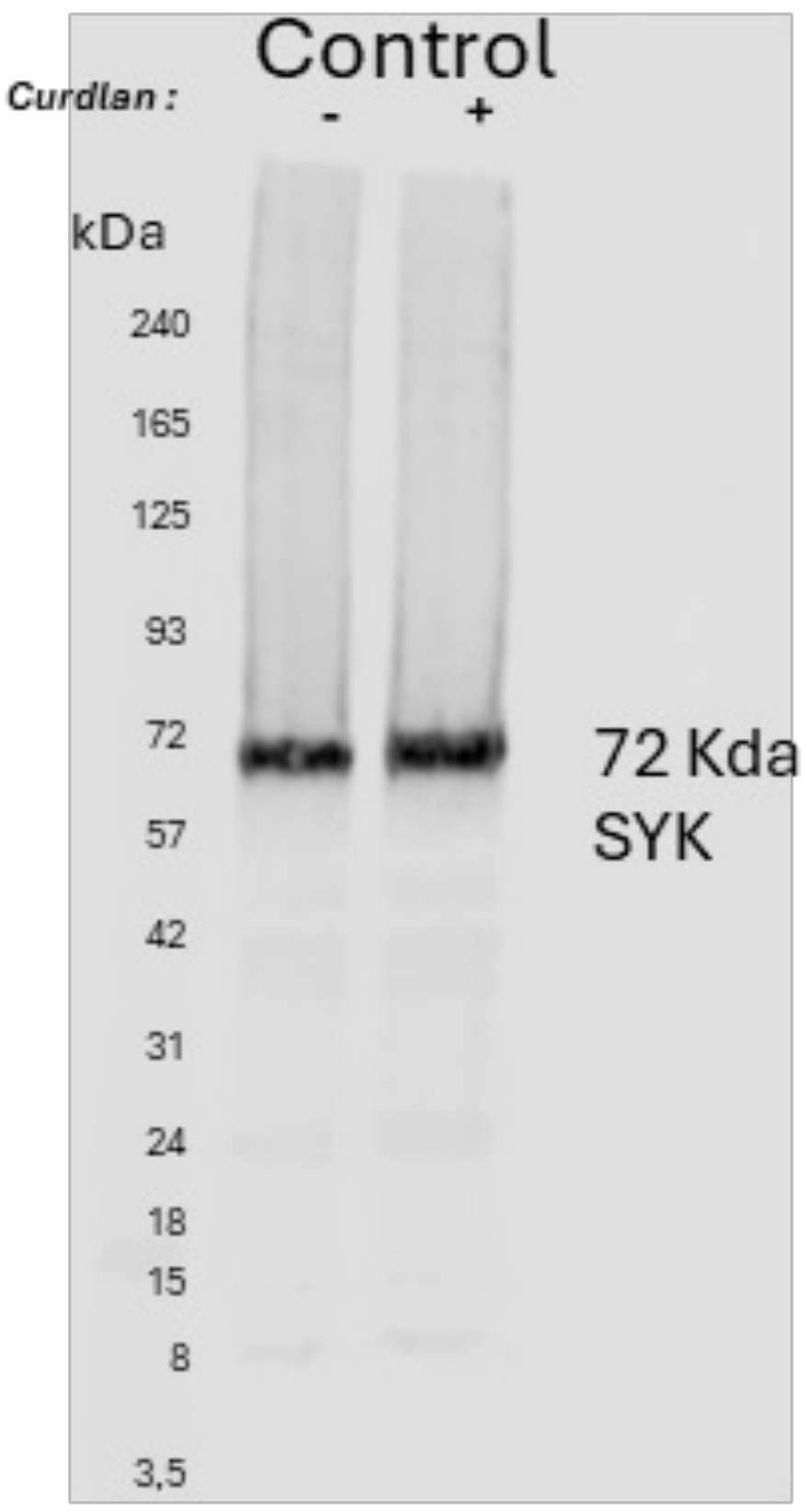

PhosphoSyk

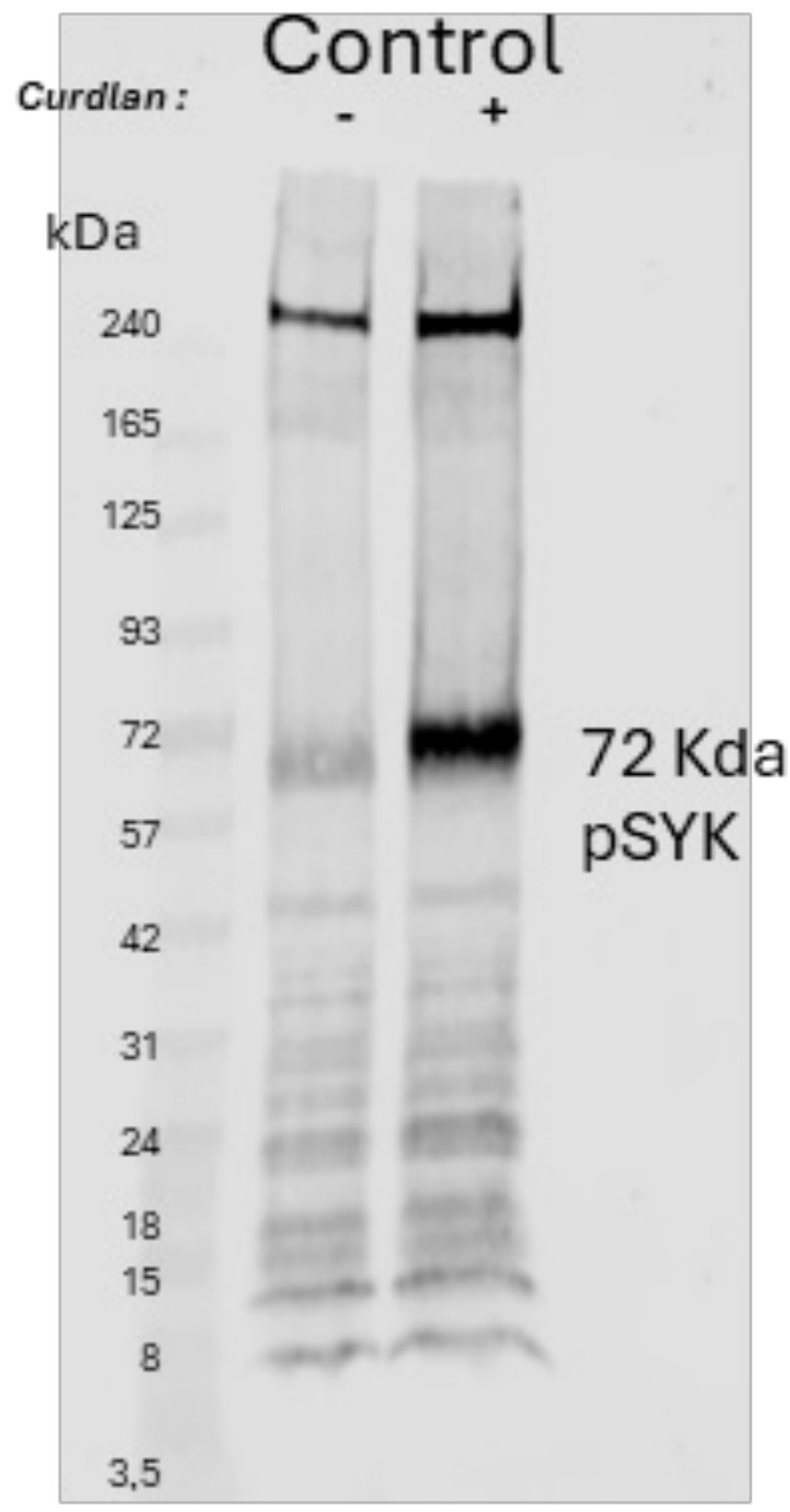

SYK

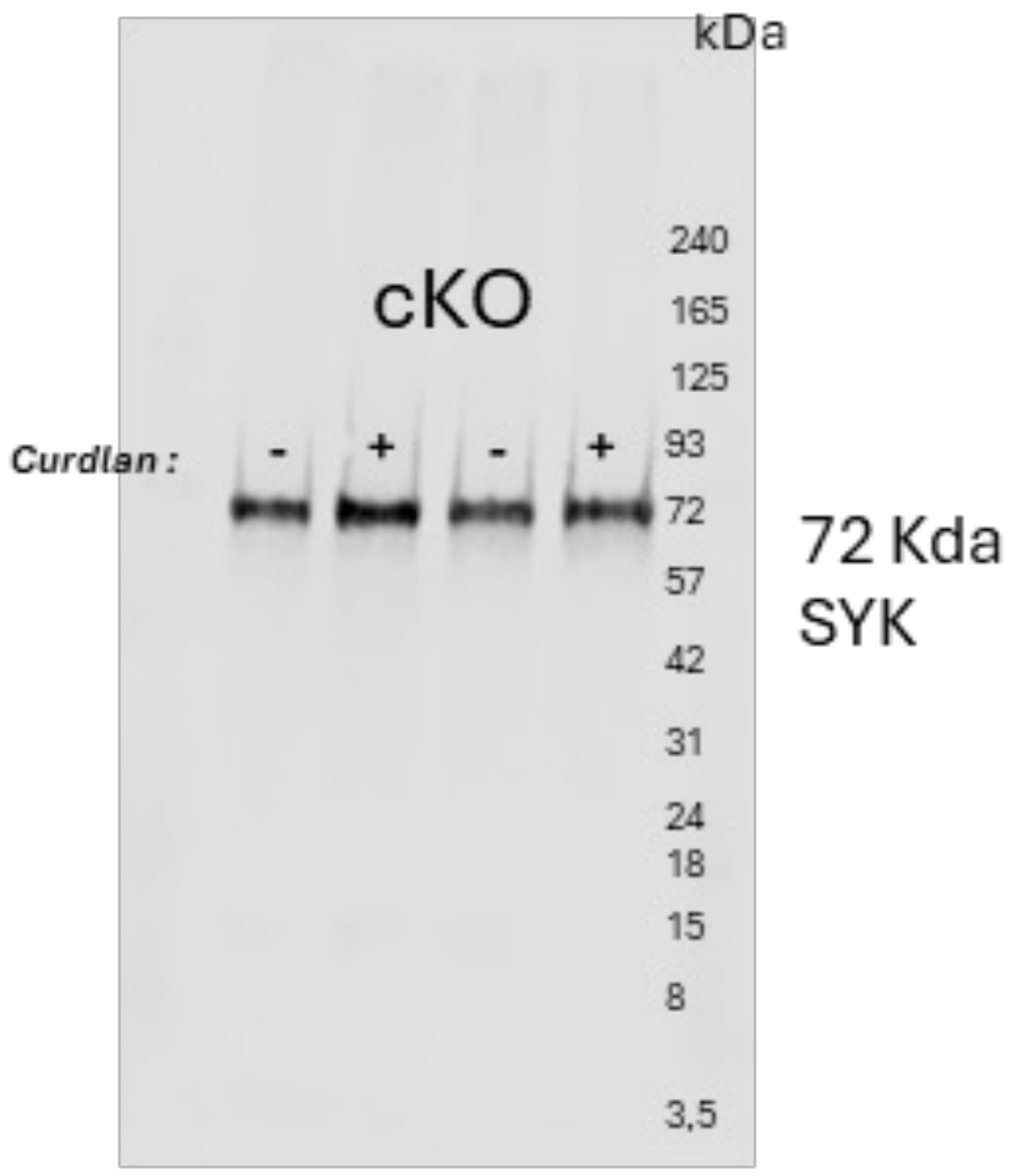

PhosphoSYK

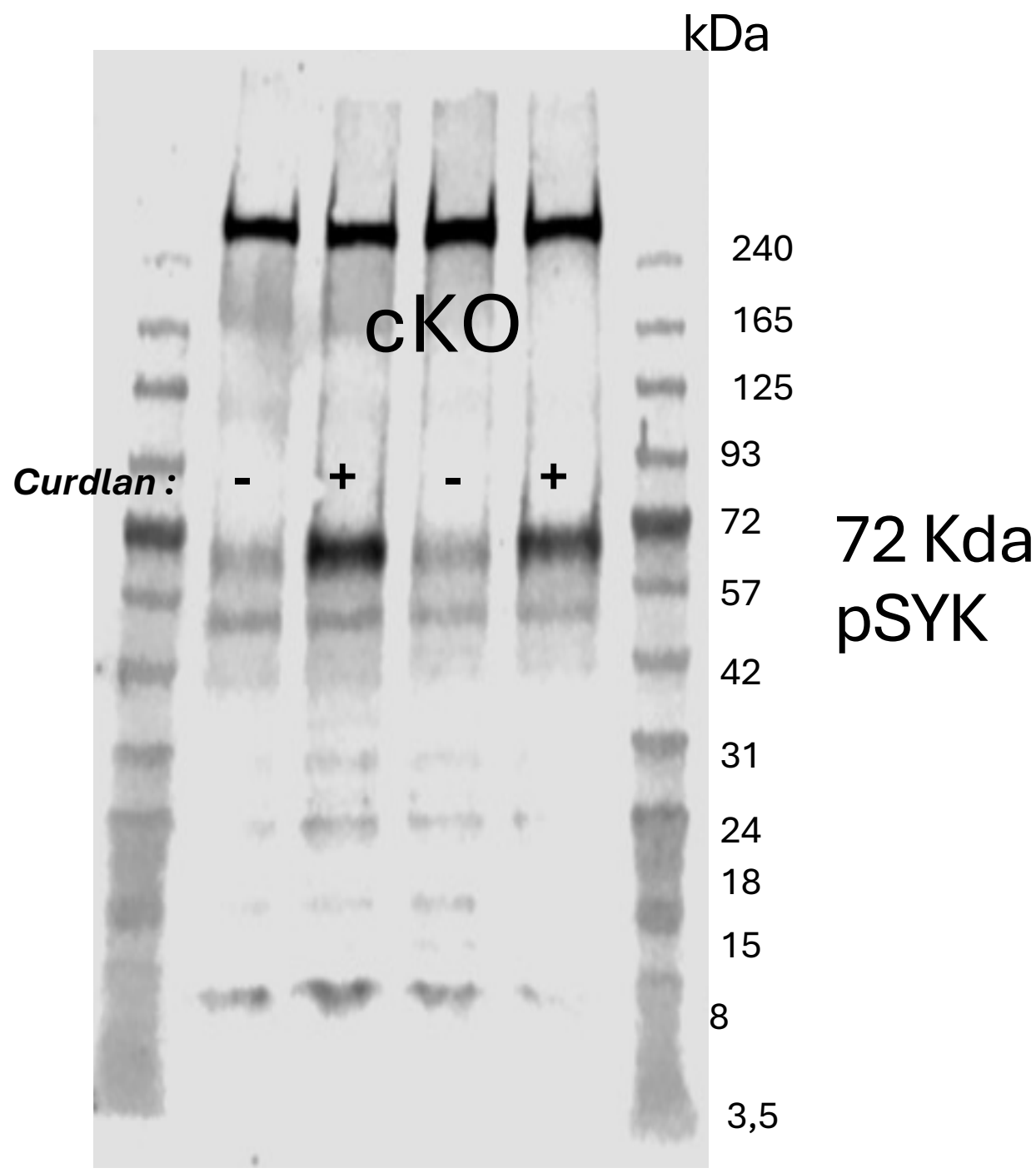

Supplement: Supplementary file 2 [file 41593_2026_2348_MOESM26_ESM.pdf]
